# Targeting FAM111B attenuates mitophagy and increases the sensitivity to lenvatinib treatment by increasing MFN2 stability in hepatocellular carcinoma

**DOI:** 10.1038/s41419-025-07941-1

**Published:** 2025-08-25

**Authors:** Yu-Chuan Yan, Li-Juan Shao, Guang-Xiao Meng, Guo-Qiang Pan, Rui-Zhe Li, Chen Xiong, Shi-jia Liu, Zi-Niu Ding, Xiao-Lu Zhang, Xiao-Feng Dong, Ying Qu, Zhao-Ru Dong, Tao Li

**Affiliations:** 1 Department of General Surgery, Qilu Hospital of Shandong University, Jinan, China; 2 Research Center for Basic Medical Sciences, Qilu Hospital of Shandong University, Jinan, China; 3 The Model Animal Research Center, Shandong University, Jinan, China; 4 Center of Hepato-Pancreato-Biliary Surgery, The First Affiliated Hospital, Sun Yat-sen University, Guangzhou, China; 5 Department of Physiology and Pathophysiology, School of Basic Medical Sciences, Cheeloo College of Medicine, Shandong University, Jinan, China; 6 Department of Hepatobiliary, Pancreas and Spleen Surgery, the People’s Hospital of Guangxi Zhuang Autonomous Region (Guangxi Academy of Medical Sciences), Nanning, China; 7 Department of Pharmacy, The Second Hospital of Shandong University, Cheeloo College of Medicine, Shandong University, Jinan, China

**Keywords:** Liver cancer, Cancer therapeutic resistance, Mitophagy, RNAi therapy, Cancer metabolism

## Abstract

Lenvatinib resistance significantly limits its clinical efficacy and application in the treatment of hepatocellular carcinoma (HCC). Mitofusin 2 (MFN2) is an important GTPase involved in mitochondrial fusion, energy balance and mitophagy. The role and regulatory mechanism of MFN2 in HCC progression and lenvatinib resistance remain unclear. Herein, we demonstrated that the family with sequence similarity 111 member B (FAM111B) regulated the stability of MFN2 and the sensitivity to lenvatinib in HCC. Mechanistically, FAM111B promoted MFN2 ubiquitination by recruiting RAN-binding protein 9 (RANBP9), a core subunit of the C-terminal to LisH (CTLH) E3 ligase complex. Targeting FAM111B generated hyperfused mitochondria, driving a metabolic shift from glycolysis to oxidative phosphorylation (OXPHOS) and antagonising cytoprotective mitophagy. Clinically, FAM111B protein levels were inversely correlated with MFN2 expression in HCC samples, with patients who exhibited high FAM111B levels having a worse prognosis and reduced sensitivity to lenvatinib treatment. More importantly, we developed glypican-3 (GPC3)-targeted lipid nanoparticles for efficient delivery of siFAM111B, which demonstrated strong efficacy in combination with lenvatinib. Together, our findings uncover a novel regulatory mechanism for MFN2 posttranscriptional regulation and highlight the therapeutic potential of targeting FAM111B in HCC treatment.

## Introduction

Hepatocellular carcinoma (HCC) is one of the leading causes of cancer-related death worldwide [1]. The majority of HCC patients are already in the advanced stage when diagnosed, and treatment options are very limited. As a first-line tyrosine kinase inhibitor for patients with advanced HCC, lenvatinib is superior to sorafenib in terms of the higher objective response rate and better tolerability [2]. However, resistance to lenvatinib significantly limits its clinical efficacy and application [3]. Uncovering the mechanisms underlying lenvatinib resistance and screening for novel therapeutic targets are crucial to improve the prognosis and life quality of HCC patients.

Heterogeneous evolution of tumours promotes the development of drug resistance. In the process of acquiring lenvatinib resistance, HCC cells develop adaptive adjustments in the mitochondrial network and metabolic patterns, which are specifically manifested by increased glycolysis (Warburg effect), impaired oxidative phosphorylation (OXPHOS), and decreased reactive oxygen species (ROS) levels [4]. Mitochondria, as highly dynamic and plastic organelles, not only play a pivotal role in maintaining energy homeostasis but also undergo continuous fission and fusion, which leads to the renewal and turnover of intramitochondrial contents [5, 6]. ROS generated from OXPHOS and induced by various stressors disrupt mitochondrial function, which interferes with critical cellular processes, including energy production, cell cycle regulation, calcium homeostasis and apoptosis, thereby leading to an abnormal cellular fate [7]. Mitophagy, a targeted form of selective autophagy, is a key mechanism by which cells eliminate dysfunctional mitochondria. This process helps reduce ROS accumulation, mitigate mitochondrial damage, and preserve mitochondrial quality. Lenvatinib-resistant tumour cells exhibit constant and rapid mitophagy flux, which serves as a protective mechanism to counteract cellular stress induced by targeted therapy [8, 9]. Consequently, mitochondrial dynamics in various cancers undergo adaptive evolution. Mitochondria tend to be fragmented in several malignant tumours, contributing to the clearance of impaired mitochondria or their components through cytoprotective mitophagy [10–13]. Moreover, decreased fusion or increased fission is positively associated with impaired OXPHOS and mtDNA modulation [14, 15]. We still do not know enough about how tumour cells in HCC regulate mitochondrial dynamics and mitophagy to resist lenvatinib treatment.

Owing to the double-membrane structure of mitochondria, mitochondrial fusion includes outer mitochondrial membrane (OMM) fusion and inner mitochondrial membrane (IMM) fusion. The fusion of OMMs initially starts with the activation of GTPase, followed by an irreversible GTP hydrolysis-driven fusion reaction, whereas optic atrophy protein 1 (OPA1) on the IMM is responsible for IMM fusion [16]. As an important GTPase and endoplasmic reticulum (ER)–mitochondria-tethering factor, mitofusin 2 (MFN2) is involved in mitochondrial fusion, metabolite transfer, energy balance and cell apoptosis [17]. As a direct target of p53, MFN2 expression is downregulated in HCC, indicating that MFN2 functions as a tumour suppressor [18]. The upregulation of MFN2 expression not only induces HCC cell apoptosis by increasing mitochondrial Ca^2+^ influx, caspase activation, the Bax/Bcl2 ratio, but also increases cell cycle arrest by regulating p21, p27 and PCNA levels [19–21]. The regulatory mechanism of MFN2 expression in HCC has not been fully elucidated.

Herein, we identified family with sequence similarity 111, member B (FAM111B), as a crucial oncogene associated with HCC progression and targeted therapy resistance. Targeting FAM111B in HCC cells produced hyperfused mitochondria and drove a metabolic switch from glycolysis to OXPHOS, resulting in impaired mitophagy flux. Our findings also revealed a new crucial mechanism by which MFN2 ubiquitination was modulated by FAM111B-recruited CTLH/RANBP9 E3 ligase in HCC. More importantly, blocking FAM111B via a small interfering RNA (siRNA) delivered by glypican-3 (GPC3)-targeted lipid nanoparticles (sGLNPs) successfully inhibited HCC progression and enhanced the lenvatinib effect in HCC treatment.

## Results

### Targeting FAM111B inhibits HCC progression and reverses lenvatinib resistance in vitro and in vivo

**Fig. 1:**
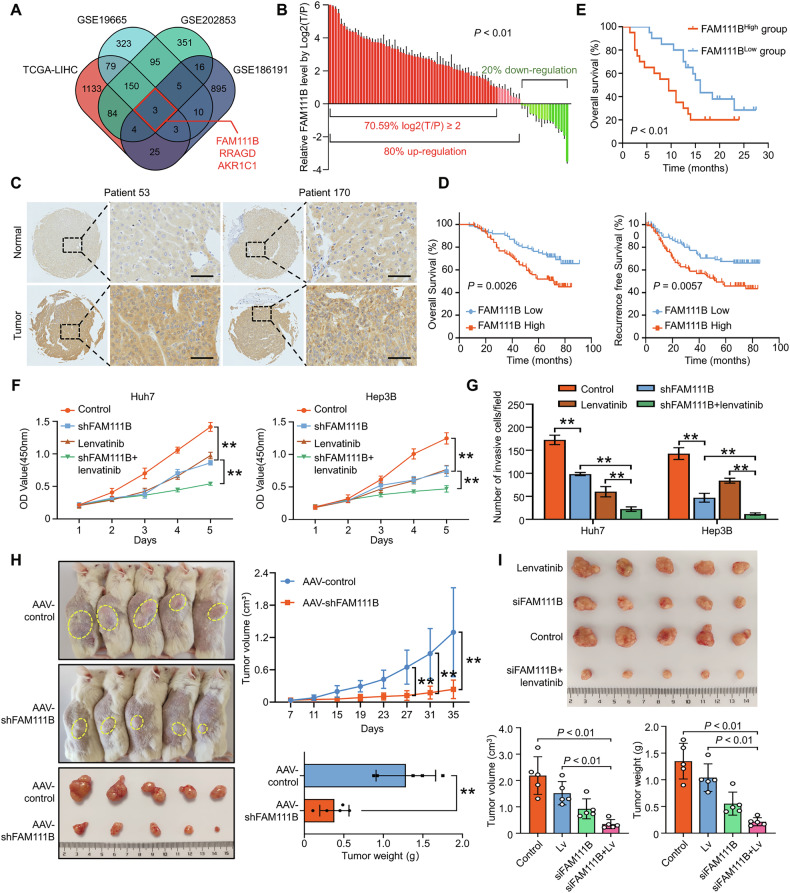
Targeting FAM111B inhibits HCC progression in vivo. **A** Venn plot showed the common DEGs between the TCGA-LIHC database and the presented GEO datasets. **B** RT‒qRCR of HCC samples and IHC staining of HCC TMA (**C**) confirmed that FAM111B was highly expressed in HCC, scale bar: 100 μm. **D** Kaplan–Meier analysis of the TMA data indicated that high FAM111B expression in HCC tissues was correlated with worse OS and RFS. **E** Kaplan‒Meier analysis showing the difference in prognosis between patients with high FAM111B expression and those with low-FAM111B expression under lenvatinib treatment. **F**, **G** CCK-8 and transwell assays verified the effect of shFAM111B on HCC proliferation and invasion abilities and the sensitivity to lenvatinib, **p < 0.01. **H** Endpoint tumour images of the PDX models treated with vehicle or AAV-shFAM111B, as well as the tumour growth curves for the PDX models (*n* = 5 per group, **p < 0.01). **I** Endpoint tumour images of the PDX models treated with lenvatinib, siFAM111B-GLNP, or combination therapy.

Our initial aim was to screen for a promising therapeutic target for HCC treatment and to reverse lenvatinib resistance. Through integrative analysis of the Cancer Genome Atlas (TCGA)-LIHC database, two expression profiles for HCC and normal tissues (GSE19665 and GSE202853) and an expression profile for lenvatinib-resistant HCC cells (GSE186191), we identified three genes, including FAM111B, RRAGD, and AKR1C1, as potential targets for HCC treatment. Among these genes, FAM111B was found to be correlated with the prognosis of HCC patients (Figs. 1A, S1A). These results were confirmed in our HCC samples (Fig. 1B–D). In addition, we retrospectively analysed the clinical data for 40 patients with advanced recurrent HCC who were receiving combined lenvatinib and anti-PD-1 therapy after partial liver resection and transcatheter arterial chemoembolization (TACE) treatment. Kaplan–Meier survival analysis revealed that overall survival of the patients was much lower in the FAM1111B^high^ group than in the FAM1111B^low^ group (Fig. 1E).

Subsequently, lentivirus-mediated transduction was used to knockdown FAM111B in Huh7 and Hep3B cells (Fig. S1B). Silencing FAM111B restrained the proliferation and invasion capabilities and increased the sensitivity of HCC cells to lenvatinib (Figs. 1F, G, S1C). Moreover, subcutaneous xenograft tumours derived from shFAM111B HCC cells were much smaller than those derived from control cells in BALB/c nude mice (Fig. S1D). To further evaluate the clinical efficacy of targeting FAM111B in HCC treatment, we created adeno-associated viruses (AAVs) carrying the shFAM111B sequence. Five patient-derived xenografts (PDXs) were subsequently established in M-NSG mice, and intratumoural shRNA-AAV injection remarkably retarded the growth of subcutaneous PDX tumours (Fig. 1H). More importantly, interfering with FAM111B significantly increased the sensitivity of HCC to lenvatinib treatment, as confirmed in the PDX models (Fig. 1I). These results showed that targeting FAM111B inhibited HCC progression and increased lenvatinib sensitivity in vitro and in vivo.

### Silencing FAM111B reprograms the metabolic pattern of HCC cells by driving the shift from glycolysis to OXPHOS

**Fig. 2:**
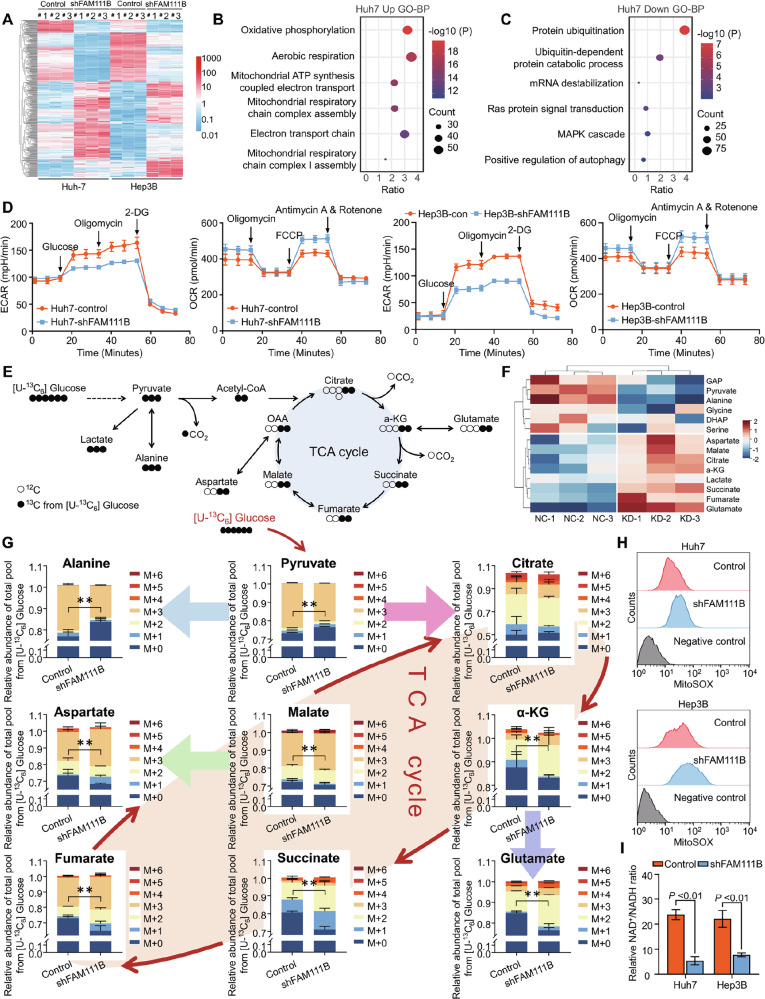
Silencing FAM111B reprograms the metabolic pattern of HCC cells. **A** Heatmap of the DEGs in shFAM111B cells compared with control cells. **B**, **C** GO analysis of upregulated and downregulated DEGs in Huh7-shFAM111B cells. **D** ECAR and OCR assays were used to assess the metabolic rate in Huh7 and Hep3B shFAM111B and control cells. **E** Diagram of the ^13^C-labelling metabolic flux assay with ^13^C_6_-Glu as the tracer. **F** Heatmap of the metabolic flux analysis showing the related incorporation ratios of several metabolites involved in glucose metabolism in Huh7 cells labelled with ^13^C_6_-Glu. **G** Mass isotopomer analysis of the aforementioned metabolites in cells cultured with [U-^13^C] glucose; **p < 0.01. **H** Flow cytometry was used to assess the effect of FAM111B knockdown on mitochondrial ROS levels. **I** The function of the electron transport chain was evaluated by calculating the NAD^+^/NADH ratio.

To elucidate the molecular mechanisms of targeting FAM111B in HCC inhibition, we established shFAM111B HCC cell lines. RNA sequencing was performed to screen differentially expressed genes (DEGs) in HCC cells after FAM111B knockdown. A total of 2602 upregulated and 2496 downregulated DEGs (fold change ≥2 and *P* < 0.05) were identified between the Huh7 control and shFAM111B cells. There were 1110 upregulated and 878 downregulated DEGs between the Hep3B control and shFAM111B cells (Figs. 2A, S2A). Gene Ontology (GO) and Kyoto Encyclopaedia of Genes and Genomes (KEGG) analyses revealed that the upregulated DEGs were enriched in biological processes such as oxidative phosphorylation (OXPHOS), aerobic respiration and the electron transport chain, whereas the downregulated DEGs were enriched in the MAPK signalling cascade, protein ubiquitination and autophagy (Figs. 2B, C, S2B–K). These results suggest that targeting FAM111B may regulate the processes of mitochondrial respiration and OXPHOS to inhibit HCC progression.

To investigate the effect of FAM111B on mitochondrial respiration, Seahorse assays were performed, revealing a decrease in the extracellular acidification rate (ECAR) and an increase in the oxygen consumption rate (OCR) in the shFAM111B groups compared with those in the control group (Figs. 2D, S2L). To determine whether FAM111B perturbs glucose metabolism, ^13^C-labelled metabolic flux analysis using U-^13^C_6_ glucose was carried out (Fig. 2E, F). Metabolic tracking revealed decreased abundances of M3 pyruvate and M3 alanine in the shFAM111B groups compared with the control group and obviously increased abundances of TCA cycle metabolites, such as M2 citrate, M2 α-KG, M2 succinate, M2 fumarate, M3 malate, M2 glutamate and M3 aspartate, in the shFAM111B groups compared with the control group (Figs. 2G, S2M). More importantly, increased ROS, impaired mitochondrial membrane potential, and a reduced NAD^+^/NADH ratio were observed in FAM111B knockdown HCC cells (Figs. 2H, I, S3N, O). These findings establish the major metabolic reprogramming mechanism mediated by silencing FAM111B, evidently through the activation of OXPHOS.

### FAM111B knockdown induces mitophagy to inhibit HCC progression and lenvatinib resistance

**Fig. 3:**
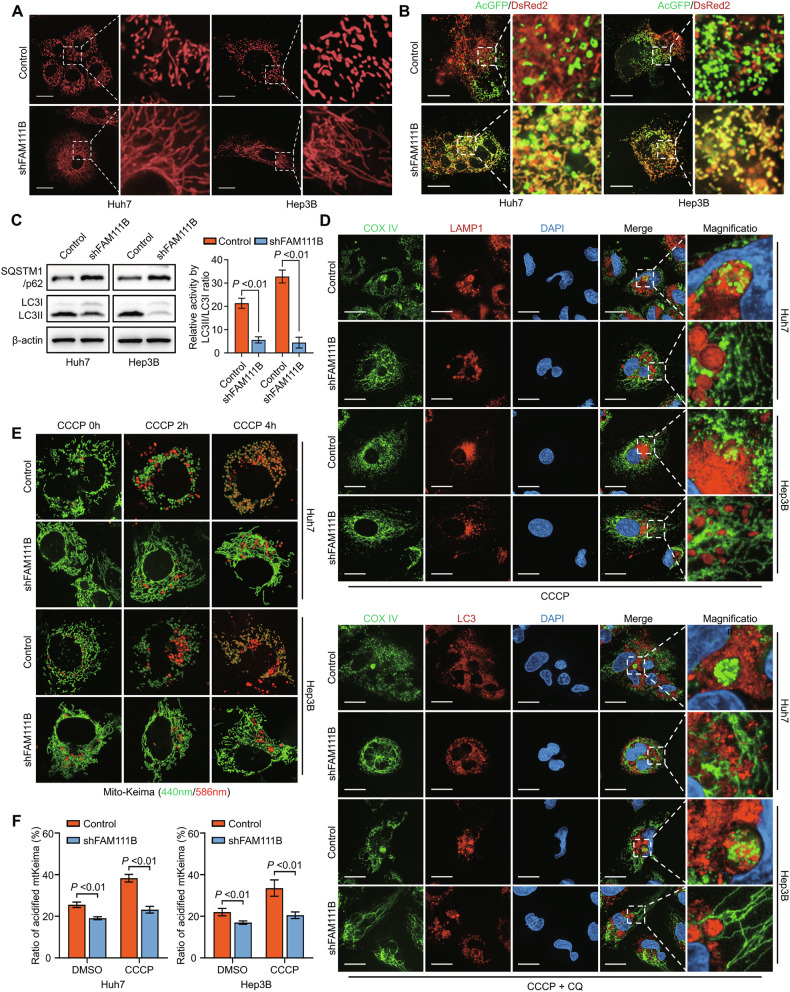
Knockdown of FAM111B induces mitochondrial fragmentation and mitophagy. **A** Representative confocal images showing the effect of FAM111B knockdown on mitochondrial morphology in Huh7 and Hep3B cells. Scale bars: 10 μm. **B** Representative confocal images showing the effect of FAM111B knockdown on mitochondrial fusion in Huh7 and Hep3B cells. Scale bars: 10 μm. **C** Western blot results showing the expression of SQSTM1 and the activation level of LC3. **D** Immunofluorescence assays were used to assess the colocalization of mitochondria and lysosomes in control and shFAM111B HCC cells. Scale bars: 10 μm. **E** Representative confocal images showing acidified (red) and unacidified (green) mitochondria labelled with the mito-Keima protein in control and shFAM111B HCC cells. **F** Statistics analysis of flow cytometry demonstrated the ratio of acidified mitochondria in control and shFAM111B HCC cells.

Mitochondrial fragmentation is associated with a shift in metabolic patterns characterised by impaired OXPHOS and enhanced glycolysis [16, 22]. Therefore, we evaluated the influence of FAM111B knockdown on mitochondrial fragmentation. As expected, FAM111B knockdown dramatically decreased the fragmentation of mitochondria, as determined by confocal microscopy (Fig. 3A). Furthermore, FAM111B knockdown significantly enhanced the fusion activity of mitochondria in HCC cells (Fig. 3B).

Given that mitochondrial fission triggers high-flux mitophagy to eliminate dysfunctional mitochondria and maintain mitochondrial quality [17, 18], we investigated the effect of FAM111B-KD on mitophagy in HCC cells. First, silencing FAM111B significantly impaired the activation levels of LC3II and p62, supporting disrupted mitophagy flux in FAM111B-KD cells (Fig. 3C). Immunofluorescence assays demonstrated reduced colocalization of mitochondria with autophagosomes or lysosomes in FAM111B-KD HCC cells (Fig. 3D). Then, plasmid vectors expressing the mito-Keima sequence were constructed and transfected into HCC cells. Confocal imaging and flow cytometry confirmed that FAM111B knockdown decreased mitophagy flux (Figs. 3E, F, S3A). These data indicate that disrupting FAM111B expression impairs mitophagy to inhibit HCC progression.

As an oral multikinase inhibitor, lenvatinib has been reported to activate mitophagy in HCC cells, and combination therapy with lenvatinib and mitophagy inhibitors may achieve the best efficacy in HCC treatment [8]. We postulated that the knockdown of FAM111B might limit the effects of lenvatinib on mitophagy. To confirm this hypothesis, we assessed changes in mitophagy in FAM111B-KD HCC cells treated with lenvatinib. As expected, FAM111B knockdown clearly inhibited lenvatinib-induced mitophagy activation when the lenvatinib concentration reached 10 µM (Fig. S3B).

### MFN2 is the crucial downstream target of FAM111B in regulating HCC metabolic reprogramming and mitophagy

**Fig. 4:**
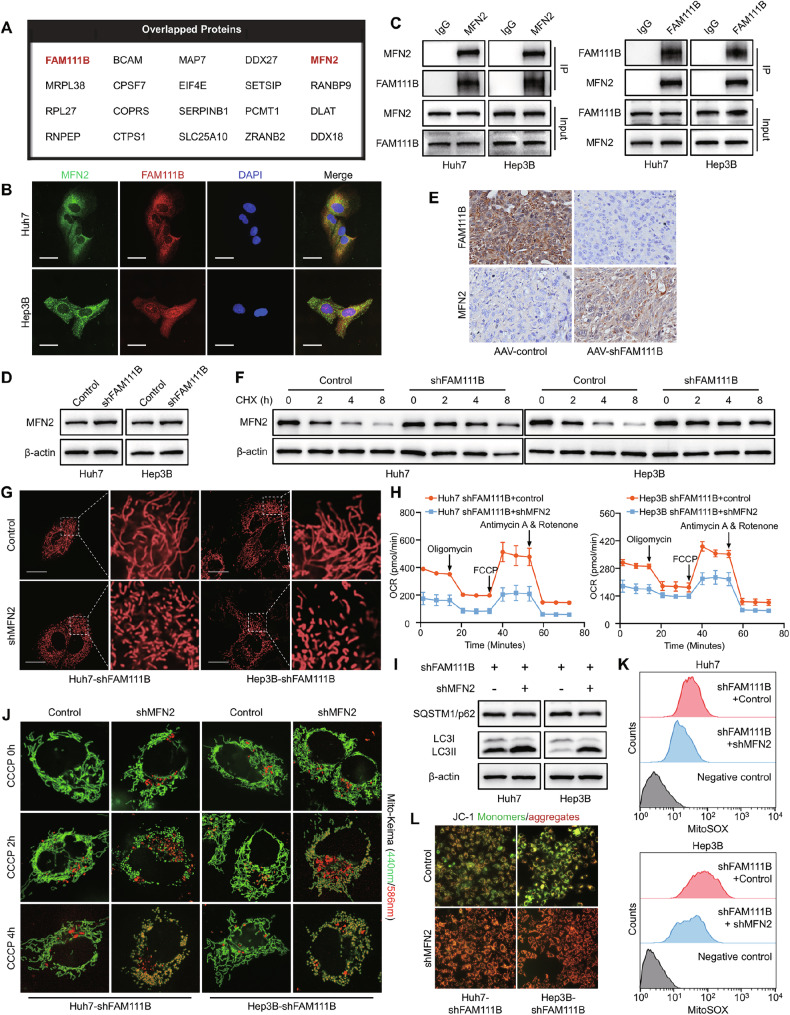
FAM111B regulates glucose metabolism and mitochondrial homeostasis by inhibiting MFN2 expression. **A** Twenty proteins that commonly interact with FAM111B in Huh7 and Hep3B cells were identified via 2D-LS/MS analysis. **B** Immunofluorescence assays revealed the colocalization of FAM111B and MFN2 in HCC cells. Scale bars: 10 μm. **C** Co-IP was used to assess the interaction between FAM111B and MFN2. **D** Western blot and IHC staining (**E**) results showing that FAM111B knockdown increased MFN2 expression in vitro and in vivo. **F** Cycloheximide chase analysis (10 μM) revealed that FAM111B knockdown significantly suppressed MFN2 degradation. **G** Representative confocal images showing the effect of MFN2 knockdown on mitochondrial morphology in shFAM111B and control HCC cells. Scale bars: 10 μm. **H** Seahorse assays revealed the effect of MFN2 knockdown on the mitochondrial respiratory stress of FAM111B-KD cells. **I** Western blot results showing the effects of MFN2 knockdown on SQSTM1 expression and LC3 activation in FAM111B-KD cells. **J** Representative confocal images showing the effect of MFN2 knockdown on mitochondrial acidification in FAM111B-KD cells. **K**, **L** MitoSOX and JC-1 probes were used to evaluate the mitochondrial ROS level and mitochondrial membrane potential in FAM111B-KD + MFN2-KD HCC cells.

To determine how FAM111B influences HCC metabolic reprogramming and mitophagy, a combination of Co-IP and 2D-LS/MS was performed to identify the interactome of FAM111B in HCC cells. Using this approach, 211 and 350 proteins were identified as FAM111B interactomes in Huh7 and Hep3B cells, respectively. Among the two sets of proteins, we identified 20 overlapping proteins, including MFN2, a dynamin-related GTPase that mediates the fusion of OMM in the two HCC cell lines (Figs. 4A, S4A). We subsequently attempted to elucidate the biochemical relationship between the FAM111B and MFN2 proteins in HCC cells. First, immunofluorescence assays revealed colocalization between FAM111B and MFN2 in HCC cells (Fig. 4B). Reciprocal Co-IP assays also confirmed that FAM111B formed a complex with MFN2 in HCC cells (Fig. 4C). We found that the inhibition of FAM111B markedly elevated MFN2 expression in vitro and in vivo, but that MFN2 did not affect the expression of FAM111B (Figs. 4D, E, S4B). Cycloheximide chase analysis revealed that FAM111B knockdown substantially arrested the degradation of MFN2 with or without carbonyl cyanide 3-chlorophenylhydrazone (CCCP), an oxidative phosphorylation (OXPHOS) uncoupler (Figs. 4F, S4C). These results suggest that FAM111B forms a complex with MFN2 to regulate the degradation of the MFN2 protein in HCC cells.

To determine whether FAM111B regulates OXPHOS through MFN2, we knocked down MFN2 in FAM111B-KD HCC cells (Fig. S4D, E). As expected, downregulating MFN2 expression caused severe mitochondrial fragmentation (Fig. 4G). Seahorse analysis revealed that MFN2 knockdown notably restored glycolytic capacity and suppressed OXPHOS, antagonising the effects of FAM111B knockdown on HCC cells (Figs. 4H, S4F, G). Moreover, MFN2 knockdown significantly reversed the proliferation, migration, invasion and apoptosis phenotypes induced by FAM111B knockdown in HCC cells (Fig. S4H–K). Similarly, MFN2 knockdown rescued the impaired activity of LC3II and reduced p62 expression mediated by FAM111B-KD (Fig. 4I). Moreover, interfering with MFN2 expression increased mitophagy flux, increased the mitochondrial membrane potential and decreased the ROS level in FAM111B-KD cells (Figs. 4J–L, S4L). These findings confirm that MFN2 is the downstream molecule of FAM111B that regulates HCC metabolic reprogramming and mitophagy.

### FAM111B recruits the RANBP9/CTLH complex for the ubiquitin-mediated degradation of the MFN2 protein

**Fig. 5:**
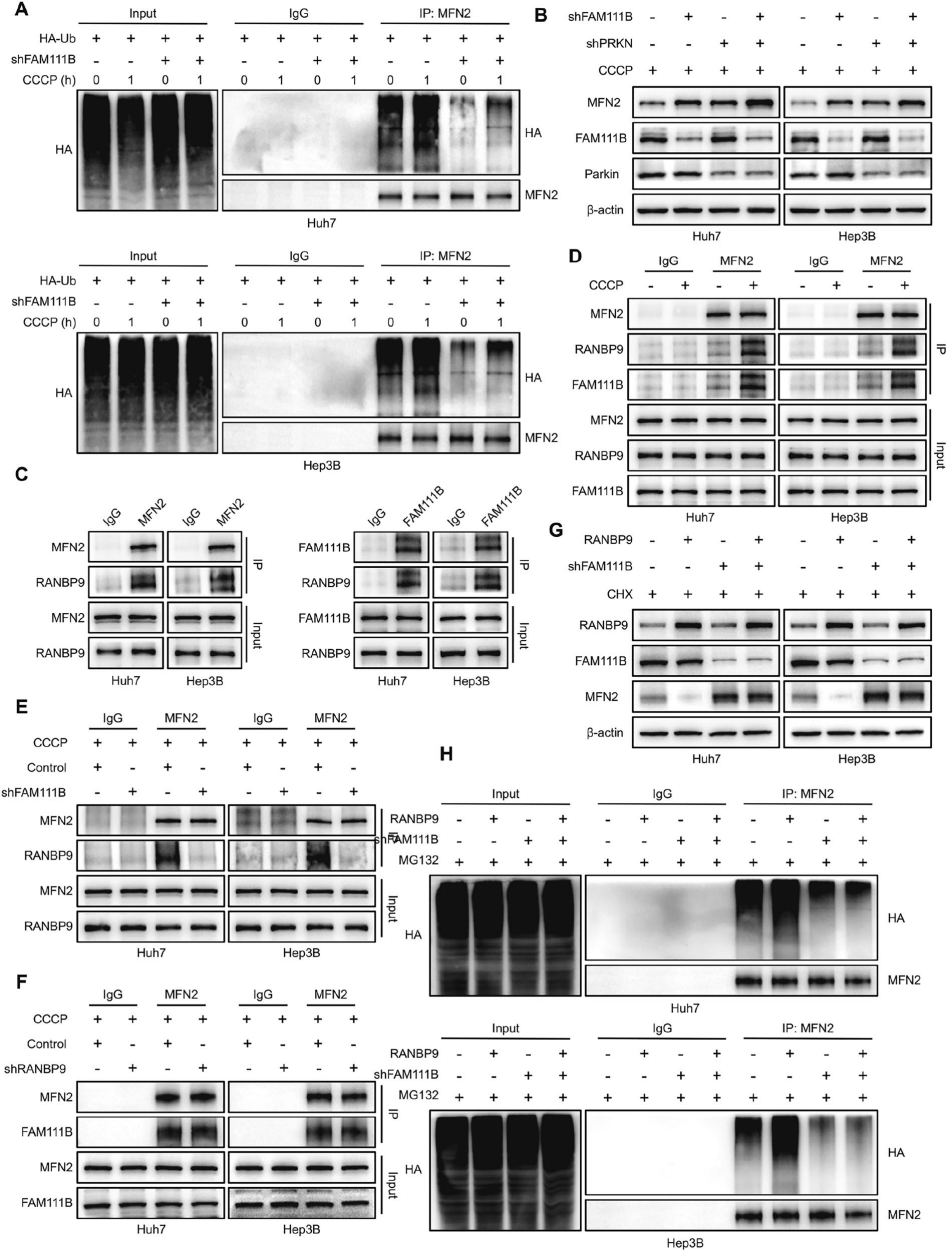
FAM111B recruits E3 ubiquitin ligases for the ubiquitin-mediated degradation of MFN2. **A** Co-IP assays showing the effect of FAM111B knockdown on MFN2 ubiquitination. **B** Western blot results indicated that FAM111B knockdown further elevated MFN2 expression induced by PRKN knockdown. **C** Co-IP was used to assess the interaction among FAM111B, MFN2 and RANBP9. **D** Co-IP results indicated increased RANBP9 and FAM111B interaction with MFN2 under CCCP stimulation. **E** Co-IP results indicated that FAM111B-KD significantly disrupted the interaction between MFN2 and RANBP9, whereas RANBP9 knockdown had no effect on the FAM111B-MFN2 interaction (**F**). **G**, **H** Co-IP and Western blot results revealed that FAM111B plays key roles in the RANBP9-mediated degradation of ubiquitinated MFN2.

In response to the loss of the mitochondrial membrane potential, pathways involving the ubiquitin E3 ligase Parkin play important roles in inducing the ubiquitination and degradation of mitochondrial proteins such as MFN2, thereby promoting the process of mitophagy in mammals [23]. To determine whether FAM111B promotes MFN2 degradation through the ubiquitin‒proteasome system, the degree of ubiquitination of MFN2 was assessed. Indeed, FAM111B knockdown significantly reduced the level of ubiquitin bound to MFN2, despite CCCP stimulation (Fig. 5A). Knocking down Parkin markedly suppressed MFN2 ubiquitination and upregulated MFN2 expression. Unexpectedly, silencing FAM111B in Parkin-KD cells further inhibited ubiquitin-mediated MFN2 degradation, suggesting that a Parkin-independent ubiquitin‒proteasome pathway is involved in the FAM111B-mediated ubiquitination and degradation of MFN2 (Figs. 5B, S5A–C).

By reanalysing the Co-IP and 2D-LS/MS data, we identified RANBP9 as a potential interacting protein of FAM111B. RANBP9 is a critical subunit of the C-terminal to lissencephaly-1 homology motif (CTLH) complex and is involved in CTLH complex stability and the recognition of substrate proteins [24–26]. To determine whether RANBP9 forms a complex with FAM111B and MFN2, we performed Co-IP experiments, which verified the interaction of RANBP9 with FAM111B and MFN2 (Fig. 5C). Moreover, increased FAM111B and RANBP9 protein binding with MFN2 was detected in CCCP-induced mitochondrial damage (Fig. 5D). Additionally, FAM111B knockdown reduced the protein level of RANBP9, whereas RANBP9 knockdown did not affect the expression of FAM111B (Fig. S5D, E). Consistently, RANBP9 significantly regulated the ubiquitin-associated degradation of MFN2 (Fig. S5F–H). To further understand the relationships among FAM111B, MFN2 and RANBP9, we verified that silencing FAM111B significantly impaired the binding affinity between RANBP9 and MFN2 (Fig. 5E). However, the knockdown of either RANBP9 or MFN2 did not influence the interaction between FAM111B-MFN2 or FAM111B-RANBP9 (Figs. 5F, S5I). More importantly, silencing FAM111B in RANBP9-overexpressing cells completely disrupted the RANBP9-related ubiquitin-mediated degradation of MFN2 (Fig. 5G, H). These data indicate that FAM111B serves as an indispensable bridge in RANBP9/CTLH complex-induced MFN2 ubiquitination.

### Different peptides of FAM111B interact with MFN2 and RANBP9 to promote the ubiquitin-mediated degradation of MFN2

**Fig. 6:**
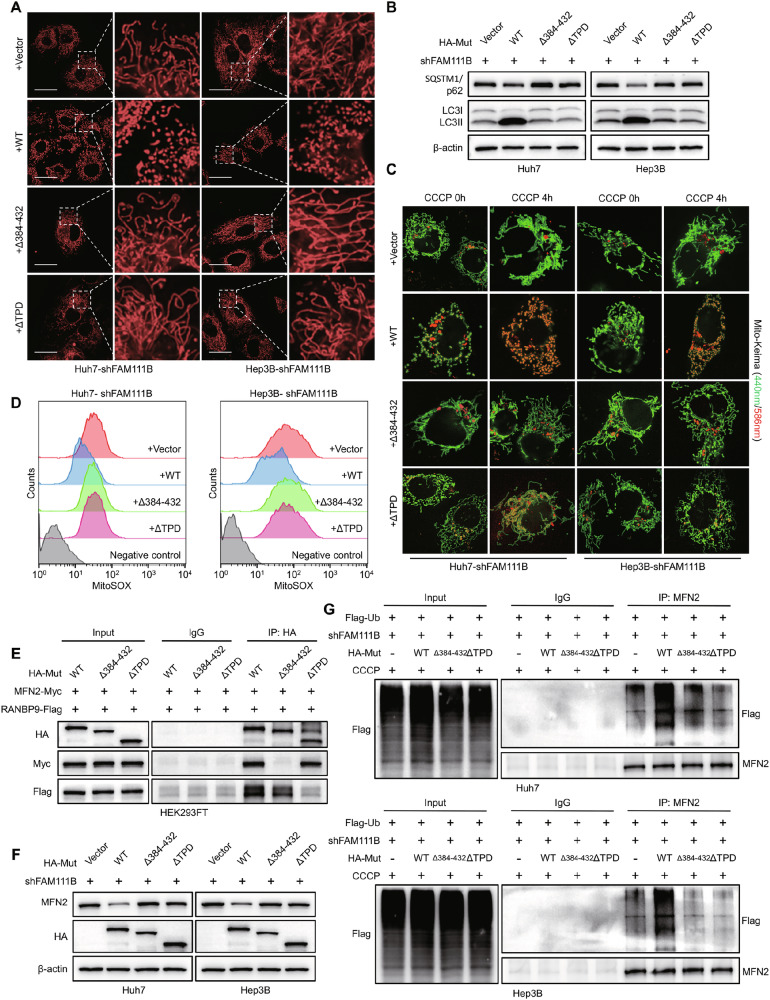
FAM111B exacerbates mitochondrial fragmentation by accelerating MFN2 degradation. **A** Representative confocal images showing the effects of exogenous wild-type or mutant FAM111B on mitochondrial morphology in Huh7 and Hep3B shFAM111B cells. Scale bars: 10 μm. **B** Western blots showing SQSTM1 and LC3 levels and representative confocal images showing mitochondrial acidification (**C**) confirmed the effect of exogenous wild-type or mutant FAM111B on mitophagy in Huh7 and Hep3B shFAM111B cells. **D** The MitoSOX probe was used to evaluate mitochondrial ROS levels in the abovementioned groups. **E** Co-IP results showing the combination patterns of MFN2 and RANBP9 with different FMA111B mutants. **F** Western blot results indicated that only WT FAM111B reversed the effect of FAM111B knockdown on MFN2 expression and ubiquitin-mediated degradation (**G**).

FAM111B contains a putative trypsin-like cysteine/serine peptidase domain (TPD) at the C-terminus, which is involved in protein‒protein interactions and cancer-promoting mechanisms [27, 28]. Given that MFN2 is the critical downstream effector of FAM111B in regulating HCC mitophagy, we further assessed the mechanisms by which FAM111B regulates ubiquitinated MFN2 degradation. The molecular docking results revealed strong binding affinity between FAM111B and MFN2, as evidenced by the hydrophobic interactions of amino acid residues L388, Y391, R413, and A428 in FAM111B with MFN2 (Fig. S6A). Then, we generated plasmid constructs encoding HA-tagged Δ384–432 (a mutant FAM111B lacking amino acids 384–432), HA-tagged ΔTPD (a mutant FAM111B lacking the peptidase domain), and HA-tagged wild-type FAM111B, which were cotransfected with Myc-MFN2 and Flag-RANBP9 vectors into HEK293FT cells. Mitochondrial morphology and mitophagy-related experiments revealed that the FAM111B-KD-induced elongation of mitochondria and impairment of mitophagy flux were rescued by wild-type FAM111B but not the Δ384–432 or ΔTPD FAM111B mutants (Fig. 6A–C). Only wild-type FAM111B, not the Δ384–432 or ΔTPD FAM111B mutants, reversed the cellular respiratory phenotype and decreased the mitochondrial membrane potential, as did excess ROS in FAM111B-KD HCC cells (Figs. 6D, S6B–G). These results indicated that both the peptide of amino acids 384–432 and the TPD domain are indispensable in FAM111B-induced HCC mitophagy.

We further investigated the effects of different regions of FAM111B in interacting with MFN2 and RANBP9. The deletion of the TPD region in FAM111B did not affect its interaction with MFN2, but the deletion of amino acids 384–432 of FAM111B eliminated the interaction with MFN2. Interestingly, both wild-type FAM111B and the Δ384–432 FAM111B mutant, not the ΔTPD FAM111B mutant, interacted with RANBP9 (Fig. 6E). Several truncation mutants were subsequently re-expressed in FAM111B-KD HCC cells to determine the importance of different peptides of FAM111B in regulating ubiquitinated MFN2 degradation. Compared with WT FAM111B, the introduction of either the Δ384–432 or ΔTPD FAM111B mutant failed to reduce MFN2 protein levels (Fig. 6F). Co-IP results revealed that WT FAM111B but not the Δ384–432 or ΔTPD FAM111B mutant rescued the suppressed ubiquitination of MFN2 induced by FAM111B knockdown (Fig. 6G). These results demonstrate that the peptide of amino acids 384–432 and the TPD domain of FAM111B interact with MFN2 and RANBP9, respectively, to mediate the RANBP9-induced ubiquitin-mediated degradation of the MFN2 protein.

### FAM111B overexpression is correlated with MFN2 silencing and poor clinical outcomes in HCC patients

**Fig. 7:**
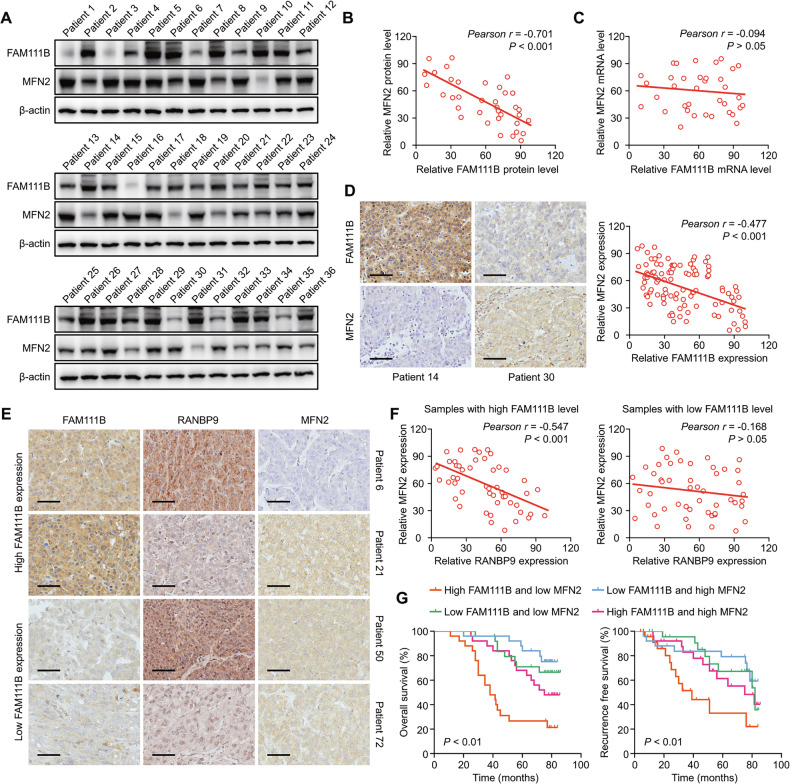
High FAM111B levels are correlated with MFN2 silencing and poor prognosis in HCC patients. **A**, **B** Western blots showing the expression and negative correlation between FAM111B and MFN2 in 36 HCC patient tissues. **C** The qPCR results revealed a nonsignificant correlation between the mRNA levels of FAM111B and MFN2. **D** IHC staining of the HCC tissue microarray was used to verify the significant negative correlation between FAM111B and MFN2 protein levels in 100 HCC patients, scale bar: 100 μm. **E**, **F** IHC staining revealed a notable negative correlation between RANBP9 and MFN2 in the high FAM111B group and a nonsignificant correlation between RANBP9 and MFN2 in the low-FAM111B group, scale bar: 100 μm. **G** Kaplan‒Meier analysis demonstrated that HCC patients with high FAM111B and low MFN2 expression had shorter OS and RFS times than other HCC patients did.

We sought to determine whether our observations could be verified in human HCC patients. To address this aim, we assessed the mRNA and protein levels of FAM111B and MFN2 in 20 HCC tissues. Consistent with our functional studies, there was a negative correlation between FAM111B and MFN2 at the protein level but not the mRNA level in HCC (Fig. 7A–C). Semiquantitative IHC analysis of TMAs from 100 HCC patients was subsequently used to further explore the correlation between FAM111B and MFN2. In this analysis, FAM111B expression was directly inversely related to the expression of MFN2 in HCC patients (Fig. 7D, Tables S1–3). These observations support the notion that FAM111B regulates the expression of MFN2 in human HCC samples.

Finally, we examined whether the interaction between FAM111B and RANBP9 can explain their effects on MFN2 expression in human HCC tissues. We found a clear negative correlation between the expression of RANBP9 and MFN2 in FAM111B^high^ HCC samples. This correlation between RANBP9 and MFN2 expression was not statistically significant in the FAM111B^low^ HCC samples (Fig. 7E, F). More importantly, HCC patients with high FAM111B and low MFN2 expression had the worst prognosis in terms of OS and cumulative recurrence rate (Fig. 7G). These results reveal that FAM111B functions as a crucial and unexpected switch for HCC progression by regulating the interaction of RANBP9 and MFN2.

### A targeted RNA delivery system carrying siFAM111B precisely disrupts HCC progression

**Fig. 8:**
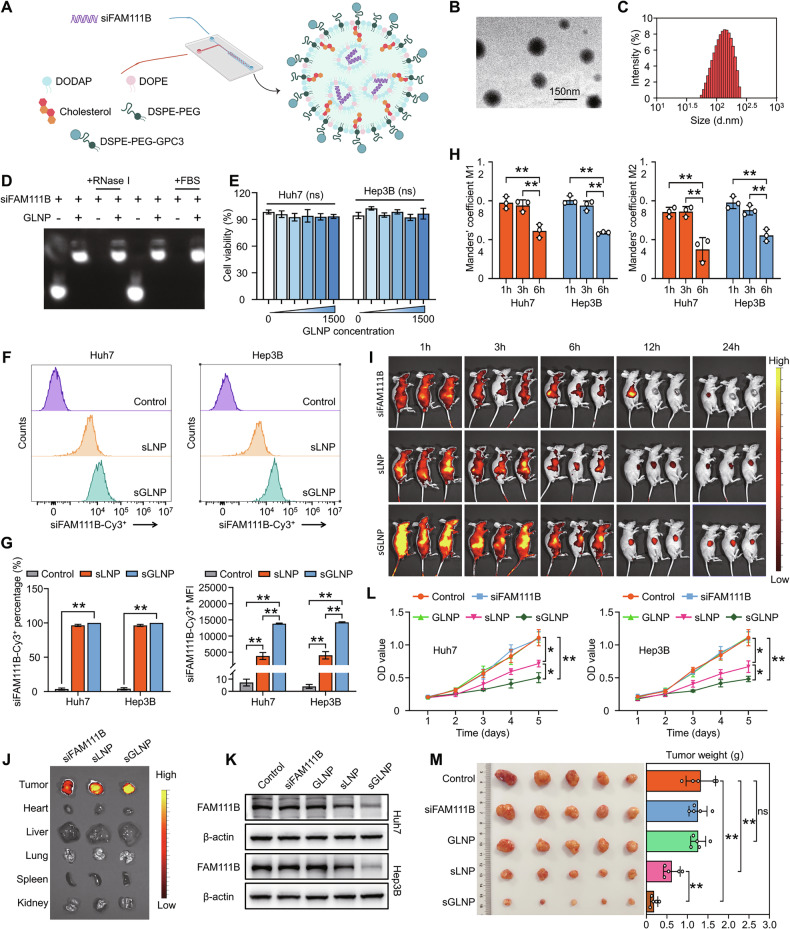
GPC3-targeted LNP carrying the siFAM111B sequence precisely blocked HCC progression. **A** Diagram (created with BioRender.com) showing the fabrication of sGLNP. **B** Representative TEM image showing the spherical morphology of sGLNP. **C** Dynamic light scattering analysis of sGLNP particle size. **D** RNase degradation assays confirmed the effective stability of sGLNP. **E** Cell viability assessment following sGLNP treatment in HCC cells. **F**, **G** Cellular uptake of the control, sLNP or sGLNP in Huh7 and Hep3B cells, as measured via flow cytometry analysis, **p < 0.01. **H** Quantification of colocalization between sGLNP labelled with Cy3 and endo/lysosomes labelled with LysoTracker Green; **p < 0.01. **I**, **J** In vivo fluorescence imaging showing distribution of Cy5.5-tagged free siRNA, sLNP and sGLNP in xenograft models after i.v. injection. **K** Western blotting analysis of FAM111B knockdown efficiency in HCC cells treated with sGLNP and sLNP. **L** CCK-8 assays evaluating the antiproliferative effects of sLNP or sGLNP on HCC cells; *p < 0.05, **p < 0.01. **M** Tumour images of the PDX models treated with sLNP or sGLNP, as well as statistical analysis of the tumour volume, **p < 0.01.

Lipid nanoparticles (LNPs) have emerged as one of the most effective RNA delivery systems, addressing the intrinsic instability and poor membrane penetration of RNA molecules [29, 30]. Glypican-3 (GPC3), a heparan sulfate proteoglycan anchored to the cell membrane via a glycosylphosphatidylinositol anchor moiety, is a well-established molecular target in HCC [31]. Here, we engineered a GPC3-targeted lipid nanoparticle (sGLNP) for the efficient targeted delivery of siFAM111B for HCC therapy. The optimised formulation, comprising 1,2-dioleoyl-3-dimethylammonium-propane (DODAP), 1,2-dioleoyl-sn-glycero-3-phosphoethanolamine (DOPE), cholesterol, polyethylene glycol-modified 1,2-distearoyl-sn-glycero-3-phosphate-ethanolamine (DSPE-PEG) and GPC3-PEG-modified DSPE (DSPE-PEG-GPC3), was established at a mass ratio of 10:7.65:9.94:2.7:2.7 (Figs. 8A, S7A). Transmission electron microscopy (TEM) revealed the characteristic spheroid morphology of sGLNP, with an average particle size of 128.22 ± 2.04 nm and an average zeta potential of −3.81 ± 0.29 mV (Fig. 8B, C, S7B). Encapsulation studies indicated complete siRNA loading when the DODAP/siFAM111B mass ratio exceeded 20 (Fig. S7C). The sGLNP conferred strong RNase resistance, exhibited minimal cytotoxicity, and induced no hemolysis, indicating excellent biocompatibility and siRNA protection (Figs. 8D, E, S7D, E).

To assess intracellular delivery, Cy3-labelled siFAM111B was encapsulated in either GLNP (sGLNP) or LNP (sLNP). Flow cytometry and confocal microscopy confirmed rapid cellular uptake by HCC cells, with sGLNP showing a ~3.5-fold higher mean fluorescence intensity (MFI) than sLNP (Figs. 8F, G, S7F). Time-lapse imaging revealed a progressive decline, indicating effective escape of sGLNP from endo/lysosomes (Figs. 8H, S7G). To assess the biodistribution in vivo, sGLNP or sLNP were administered via tail vein injection to both subcutaneous xenograft models and orthotopic models. In vivo biodistribution studies in both subcutaneous and orthotopic HCC models showed markedly enhanced Cy5.5 signal retention in the sGLNP group compared to sLNP or naked siRNA, with preferential tumour accumulation and prolonged circulation (Figs. 8I, J, S7H–L). Notably, sGLNP efficiently silenced the expression of FAM111B and suppressed tumour proliferation in vivo (Figs. 8K, L, S7M). In PDX models, siFAM111B-GLNP significantly inhibited HCC progression and counteracted lenvatinib resistance in vivo (Figs. 8M, 1I). Collectively, these results demonstrate the potent and tumour-specific therapeutic efficacy of sGLNP-mediated FAM111B silencing, underscoring its translational promise for precision HCC therapy.

## Discussion

**Fig. 9:**
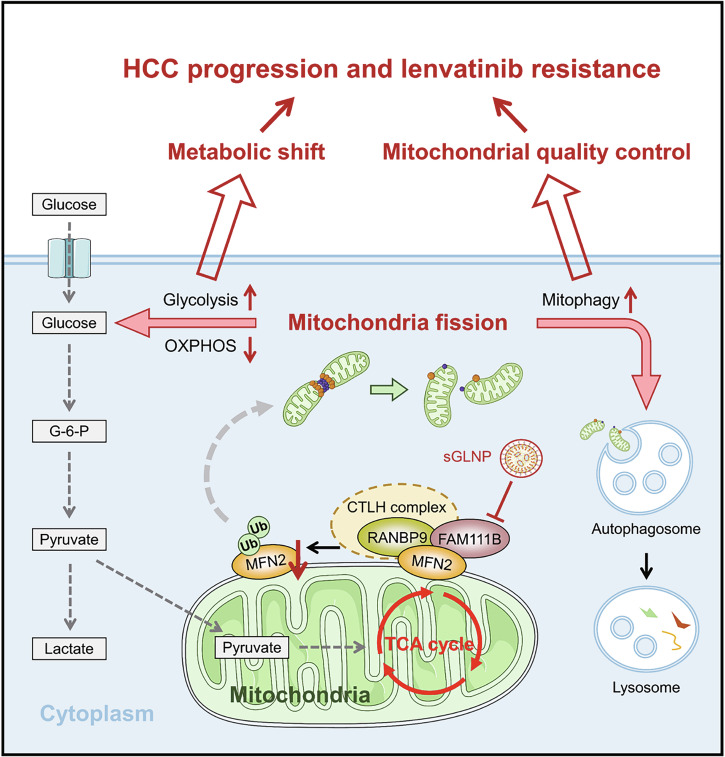
Schematic diagram depicting the mechanism by which FAM111B regulates HCC progression and lenvatinib sensitivity. FAM111B promotes MFN2 ubiquitination and degradation by recruiting RANBP9/CTLH E3 ligase complex. Targeting FAM111B generates hyperfused mitochondria, driving a metabolic shift from glycolysis to OXPHOS and antagonizing cytoprotective mitophagy. Moreover, GPC3-targeted LNPs demonstrates the efficient delivery ability of siFAM111B and exhibits strong efficacy on HCC in combination with lenvatinib treatment.

HCC is one of the most common human malignancies and the third leading cause of cancer mortality worldwide [32]. There is an urgent need for new HCC treatment strategies because of the unsatisfactory prognosis of patients. Herein, we show that FAM111B acts as an oncogenic effector in HCC progression. Mechanistically, FAM111B combines with RANBP9 and strengthens the binding affinity between the CTLH E3 ligase complex and MFN2, resulting in the Parkin-independent ubiquitination of MFN2. The degradation of MFN2 mediated by FAM111B/RANBP9 leads to fragmented mitochondria and the activation of mitophagy (Fig. 9). More importantly, we show that targeting FAM111B with either adeno-associated virus (AAV) or sGLNP containing siFAM111B inhibits HCC progression in vivo, indicating a new therapeutic strategy for treating HCC.

Cancer cells constantly adapt to microenvironmental stress and adverse stimuli by remodelling metabolic patterns. Reprogrammed mitochondria play a pivotal role in cancer metabolism by providing energy for cell survival and a carbon source for nonessential amino acid and fatty acid synthesis and by generating ROS from the electron transport chain [33, 34]. Fragmented mitochondria are observed in various cancer cells. Posttranslational modifications of mitochondrial dynamic effectors contribute to mitochondrial turnover. For example, mTOR signalling pathway-induced MFN2 phosphorylation modulates the effects of the PKM2‒MFN2 interaction on glycolysis, mitochondrial fusion and OXPHOS [35]. Herein, we found that FAM111B serves as a bridge to mediate the interaction of RANBP9 and MFN2, thereby inducing the ubiquitin-mediated degradation of MFN2; however, RANBP9 does not directly interact with MFN2. Silencing FAM111B increased the expression of the MFN2 protein, reprogrammed the metabolic pattern of HCC from glycolysis to OXPHOS, and inhibited HCC progression. However, whether the downregulation of MFN2 expression causes a switch in the glycolytic/OXPHOS ratio due to OXPHOS disruption or the active enhancement of glycolysis requires further exploration.

As an evolutionarily conserved dynamic process of autophagy, mitophagy enables cancer cells to control ROS generation and resist apoptosis by selectively removing dysfunctional or superfluous mitochondria [36]. The pathways composed of PTEN-induced kinase 1 (PINK1) and Parkin, an E3 ubiquitin ligase, are important key regulators of mitophagy in mammals. In damaged mitochondria, IMM depolarisation prevents the degradation of PINK1, leading to the accumulation of full-length PINK1 in the OMM and the recruitment of Parkin. The ubiquitin ligase activity of Parkin is stimulated to facilitate the degradation of multiple OMM proteins, including MFN2. Moreover, autophagy receptors such as p62 and optineurin are recruited, causing the formation of autophagosomes to eliminate damaged mitochondria [37]. Other E3 ubiquitin proteins, such as mitochondrial E3 ubiquitin protein ligase 1 (Mul1), MARCH5, SMAD-specific E3 ubiquitin protein ligase 1 (SMURF1), and autocrine motility factor receptor (Gp78/AMFR), are involved in mitophagy regulation [38–40]. Our study revealed that the peptide of amino acids 384–432 of FAM111B interacted with MFN2, whereas the TPD domain of FAM111B interacted with RANBP9. FAM111B forms a complex with MFN2 and RANBP9 to mediate the RANBP9-induced ubiquitin-mediated degradation of MFN2. Targeting FAM111B in HCC could inhibit tumour progression and increase lenvatinib treatment sensitivity by inducing damaged mitophagy.

In summary, we determined that targeting FAM111B favours the inhibition of HCC progression by promoting RANBP9-induced MFN2 ubiquitinated degeneration and that sGLNP successfully delivered shFAM111B to inhibit HCC progression and enhance the effect of lenvatinib for HCC treatment. The results of our study reveal a promising therapeutic approach for combination therapy with lenvatinib.

## Materials and methods

### Cells and cell culture

Huh7 and Hep3B cells were gifts from Zhongshan Hospital, Fudan University. HEK293FT cells were purchased from Pricella Co., Ltd., China. All the cell lines were confirmed via STR genotyping. Huh7 cells were maintained in high-glucose DMEM supplemented with 10% foetal bovine serum, penicillin (100 IU/ml) and streptomycin (100 μg/ml). Hep3B cells were maintained in MEM-alpha supplemented with 10% foetal bovine serum, penicillin (100 IU/ml) and streptomycin (100 μg/ml). HEK293FT cells were maintained in high-glucose DMEM supplemented with 10% foetal bovine serum, G418 (500 μg/ml), penicillin (100 IU/ml) and streptomycin (100 μg/ml). All the cells were cultured in 25 cm^2^ polystyrene culture flasks in a humidified atmosphere of 5% CO_2_ at 37 °C.

### Clinical samples, patient follow-up and tissue microarray (TMA)

These experiments were approved by the Ethics Committee of Qilu Hospital of Shandong University (Jinan, China) and were performed in accordance with approved guidelines. Fresh human HCC and adjacent nontumour liver tissue samples were randomly collected from consecutive HCC patients undergoing curative resection at Qilu Hospital of Shandong University. Before the operation, informed consent was obtained from the patients to harvest residual resected tissues after pathological diagnosis. The TMA was constructed from specimens from 200 HCC patients. Clinicopathological information was collected, and follow-up procedures were performed as described previously.

### Lentivirus, plasmid and transfection experiments

The lentiviral plasmids shFAM111B, shMFN2, shPRKN, shRANBP9, RANBP9-OE, and DCP1A-OE and the control sequences were purchased from Shanghai Genechem Co., Ltd. Lentiviral constructs expressing shRNAs and CMV promoter sequences were purchased from Shanghai Genechem Co., Ltd. and were used for stable knockdown and overexpression in HCC cells, respectively. After 48 h, infected cells were cultured under selection in the presence of 5 μM puromycin. HA-FAM111B, myc-MFN2, flag-RANBP9, and flag-ubiquitin plasmids, as well as the FAM111B-Δ382-422 and FAM111B-ΔTPD mutant plasmids, were purchased from Shanghai GenePharma Co., Ltd. For plasmid transfections, cells were grown to 60% confluence in 6-well dishes and transfected with 2 μg plasmid/well in 4 μl of transfection reagent (jetPRIME, Polyplus) according to the manufacturer’s instructions. For experiments using FAM111B truncation mutant plasmids, 10 μM MG132 was added 6 h prior to harvesting the cells. The oligo sequences of the shRNAs used are provided in Supplementary Table S3.

### Preparation and characterisation of sGLNP

The acidic aqueous phase (50 mM citrate, pH 4.0) contained siFAM111B, while the organic phase consisted of a co-dissolution of 1,2-dioleoyl-3-dimethylammonium-propane (DODAP), 1,2-dioleoyl-sn-glycero-3-phosphoethanolamine (DOPE), cholesterol, Glypican-3 targeting peptide-PEG modified 1,2-distearoyl-sn-glycero-3-phosphate-ethanolamine (DSPE-PEG-GPC3), and DSPE-PEG at a specific molar ratio in alcohol. The GPC3 targeting peptides (peptide sequence: DYEMHLWWGTEL) were acquired from Chongqing Yusi Pharmaceutical Technology Co., Ltd. These phases were mixed in a microfluidic chip device using syringe pumps, with a suitable RNA to DODAP weight ratio. The resulting sGLNPs were dialysed against PBS (pH 7.4) for 12 h to remove ethanol. A similar procedure was used to prepare sLNP without DSPE-PEG-GPC3. The prepared sGLNP were then diluted 20-fold, and their particle size, polydispersity index (PDI), and zeta potential were measured using a Zetasizer Nano ZS90 (Malvern, UK).

### Loading capacity analysis of siFAM111B by sGLNP

siFAM111B was complexed with DODAP at varying weight ratios (1:2, 1:4, 1:8, 1:15, 1:20, and 1:25) in phosphate buffer at pH 7.4. The resulting sGLNP complexes were electrophoresed on a 1% (w/v) agarose gel for 40 min at 60 V and room temperature. Fluorescent imaging of the gel was performed using a fluorescence imager (Tanon, China).

### Gel retardation assay

To assess the protective effect of GLNP on siFAM111B, samples were incubated with RNase or PBS at 37 °C for 1 h. Following treatment, gel electrophoresis was performed, and the resulting gels were examined under a UV illuminator (Tanon, China) to visualise the localisation of siFAM111B.

### Hemolysis test

Whole blood was collected from rabbits, and erythrocytes were isolated via centrifugation. After two washes with PBS, the erythrocytes were co-cultured with normal saline, GLNP, sGLNP, or H₂O for 3 h. The supernatants were subsequently collected, and hemolysis rates were evaluated by measuring UV-visible absorbance at 570 nm using a microplate reader.

### Examination of cellular uptake in vitro

Hep3B and Huh7 cells were seeded at 10^5^ cells per well in a 24-well plate and allowed to adhere overnight. Subsequently, siFAM111B^Cy3^, siFAM111B^Cy3^-LNP, and siFAM111B^Cy3^-GLNP were incubated with the cells for 4 h. Following incubation, the cells were washed three times with PBS, and the nuclei were stained with DAPI (blue). The cells were then analysed using confocal laser scanning microscopy (CLSM, Dragonfly 200, Andor, UK). For qualitative assessment, flow cytometry was employed to further examine the cells.

### Endolysosomal escape

Hep3B and Huh7 cells were treated with siFAM111B^Cy3^-GLNP at 37 °C for 1, 3, and 6 h. After treatment, the cells were incubated with LysoTracker Green at 37 °C for 30 min and then fixed with 4% paraformaldehyde. Following fixation, the cells were stained with DAPI and analysed using CLSM. To quantitatively assess co-localisation between sGLNP and LysoTracker Green, Manders’ co-localisation coefficients were calculated using ImageJ.

### Animal experiments

All the mice were housed in a specific pathogen-free facility at the Laboratory Animal Center of Shandong University. In vivo studies were approved by the Animal Ethics Committee of Qilu Hospital of Shandong University. For patient-derived xenograft (PDX) models, fresh HCC tissues were rapidly cut into 2 × 2 × 2 mm^3^ fragments on ice and subcutaneously implanted in the right axilla of NOD-*Prkdc*^*scid*^*Il2rg*^*em1*^/Smoc (M-NSG, purchased from Shanghai Model Organisms Co., Ltd.) mice to amplify the tumour cells (first-passage PDX model). Then, the tumours were collected and cut into small pieces when they reached 800–1000 mm^3^ in size and were subcutaneously inoculated into the right axilla of new M-NSG mice (second-passage PDX model). When the tumours reached 200 mm^3^, the mice were randomly divided into several groups for antitumour experiments and survival assessments. Euthanasia was performed after 15 days of administration, and tumour tissue samples were collected for further analysis. For in vivo biodistribution of sGLNP, Huh7 subcutaneous tumour models were intravenously injected with siFAM111B^Cy5.5^, siFAM111B^Cy5.5^LNP, and siFAM111B^Cy5.5^GLNP. Fluorescence signals throughout the body were detected at designated time points (0, 3, 6, 12, and 24 h). After 48 h, all animals from each group were euthanised, and their organs were excised for ex vivo imaging. The biodistribution of the injected particles was assessed by imaging whole organs using an IVIS system, and the data were analysed with Living Image software.

### RNA isolation and quantitative reverse transcription–PCR (qRT–PCR)

Total RNA was extracted from tissues and HCC cell lines using an RNA Rapid Extraction Kit (RNAfast200, Fastagen, China) according to the manufacturer’s instructions. mRNA was reverse transcribed to cDNA using a HiScript III 1st Strand cDNA Synthesis Kit (Vazyme, China) according to the manufacturer’s instructions. Quantitative PCR (qPCR) was subsequently performed with ChamQ SYBR qPCR Master Mix (Vazyme, China). All the sequences of primers used for qPCR are listed in Supplementary Table S1.

### Immunohistochemical (IHC), immunofluorescence (IF) and immunoblot analyses

Immunohistochemical staining was carried out using formalin-fixed sections. After the sections were dewaxed and endogenous peroxidase activity was blocked, the sections were incubated in 10% normal goat serum. The sections were subsequently incubated overnight at 4 °C with primary antibodies at the appropriate concentrations. Then, the sections were incubated with secondary antibodies and stained with diaminobenzidine and haematoxylin. The immunoreactivity was reviewed and evaluated by two pathologists who were blinded to the clinical data. For immunofluorescence, cells cultured on glass slides or glass-bottom dishes were gently washed with ice-cold phosphate-buffered saline (PBS) and fixed with methanol. After being treated with 1% Triton X-100, the fixed cells were blocked with bovine serum albumin and incubated overnight directly with a diluted primary antibody. Then, the fixed cells were incubated with an Alexa Fluor-conjugated secondary antibody at the appropriate concentration and counterstained with DAPI (Solarbio, China). Finally, fluorescence was examined via fluorescence or confocal microscopy. For protein extraction, the cells were lysed with lysis buffer (Thermo Fisher Scientific) in the presence of a protease inhibitor and phosphatase inhibitor cocktail (Thermo Fisher Scientific). The extracted proteins were separated via sodium dodecyl sulfate–polyacrylamide gel electrophoresis (SDS–PAGE). The primary antibodies used are listed in Supplementary Table S2.

### Mitochondrial morphology and fusion analysis

Living mitochondria were labelled with MitoTracker Deep Red (Biotime) dye and photographed via confocal microscopy. For the mitochondrial fusion assay, cells expressing pAcGFP1-Mito were seeded into 96-well glass-bottom plates with the same number of cells expressing pDsRed2-Mito. The cells were fused for 60 s with 50% PEG1500 (Proteintech) the next morning, washed thoroughly with 1× PBS, and cultured for 7 h in medium containing 20 μg/mL cycloheximide before image acquisition.

### Oxidative phosphorylation and glycolysis assays

The intact cellular oxygen consumption rate (OCR) and extracellular acidification rate (ECAR) were measured in real time using a Seahorse XF96 Extracellular Flux Analyser (Agilent). Briefly, 2.0 × 10^4^ Huh7 or Hep3B cells were seeded into 96-well microplates and incubated at 37 °C in 5% CO_2_, and the calibrator plate was equilibrated in a non-CO_2_ incubator. After 24 h, the cells were washed twice with detection media (DMEM, 25 mmol/L glucose, 1 mmol/L glutamine, 1 mmol/L sodium pyruvate for the OCR; DMEM, 1 mmol/L glutamine for the ECAR) and equilibrated in a non-CO_2_ incubator for 2 h. Once probe calibration was completed, the probe plate was replaced with a cell plate. For the OCR, the analyser plotted the values as the cells were subjected to sequential injections of oligomycin, carbonyl cyanide-4 (trifluoromethoxy) phenylhydrazone (FCCP), antimycin A and rotenone. For the ECAR, the analyser plotted the values as the cells were subjected to sequential injections of glucose, oligomycin and 2-deoxy-glucose (2-DG).

### Metabolic flux analysis

Glycolytic and OXPHOS flux was measured, using LC‒MS, on the basis of the rate of glucose consumption and the ratio of ^13^C incorporated into metabolites. Briefly, Huh7 control and FAM111B knockdown cells were cultured to 50% confluence and then cultured in DMEM supplemented with 4 mmol/L [U-^13^C_6_]-glucose. After 4 h, the medium was collected, and the cells were fixed with cold 80% methanol. Metabolites were extracted and analysed via LC‒MS at Shanghai Biotree Co., Ltd. The measured mass isotopomer distributions were corrected according to their natural abundances.

### Evaluation of mitochondrial ROS and the mitochondrial membrane potential

MitoSOX Red staining was performed to detect mitochondrial ROS. In brief, Huh7 and Hep3B cells were cultured in 6-well glass-bottom culture plates. Then, the cells were washed with ice-cold 1× PBS and incubated with MitoSOX Red, MitoTracker Green, and Hoechst for 20 min at 37 °C. The fluorescence signals were captured by using both a flow cytometer (BD) and superresolution fluorescence microscopy (Nikon). JC-1 staining was performed to evaluate the mitochondrial membrane potential. Huh7 and Hep3B cells were cultured in 6-well glass-bottom culture plates. The cells were washed with ice-cold 1× PBS and incubated with JC-1 dye for 20 min at 37 °C. Immediately after incubation, the cells were washed with 1× PBS, after which complete medium was added. Fluorescence images were captured via fluorescence microscopy. Three separate sets of experiments were performed for staining.

### RNA sequencing (RNA-seq) and analysis of RNA-seq data

Total mRNA was extracted from HCC cells using a spin column kit (Fastagen Biotech) and evaluated using an RNA 6000 kit (Agilent Technologies). Then, RNA libraries were established with a TruSeq RNA library preparation kit (Illumina), quantified via quantitative real-time PCR, normalised, pooled, and sequenced using an Illumina HiSeq 4000 instrument. Statistical analysis and ggplot2 (v3.3.2) were performed using the R program v4.0.3, and a *P*-value < 0.05 was considered statistically significant.

### Statistical analysis

All the statistical analyses were performed using SPSS software (IBM SPSS Statistics, version 20.0) or GraphPad Prism software (version 8.3.1; www.graphpad.com). Unpaired Student’s *t*-test was used to compare data from two groups. The experimental results are shown as means ± SDs. A chi-square test was performed to compare the categorical variables. Survival analysis was performed using the Kaplan‒Meier method. Pearson’s correlation analysis was used to assess correlations. The independent prognostic factors for HCC were determined using univariate proportional hazards (Cox) regression and multivariate Cox regression. All experiments were repeated three times, unless otherwise specified, and P values of 0.05 were considered to indicate statistically significant differences.

## Supplementary information

Supplementary materials

Western blot data

## Data Availability

All data generated or analysed in this study are included in the published article or supplementary materials. The accession number of RNA-seq raw data in the GEO database is GSE297369. Other raw data are available from the corresponding author upon request.
