# Supplementary material for: Targeting FAM111B attenuates mitophagy and increases the sensitivity to lenvatinib treatment by increasing MFN2 stability in hepatocellular carcinoma
Source: Cell Death Dis. 2025 Aug 25;16(1):645. doi: 10.1038/s41419-025-07941-1 (PMC12379274; doi:10.1038/s41419-025-07941-1)
Supplement: Supplementary file 1 — Supplementary materials [file 41419_2025_7941_MOESM1_ESM.docx]

**Supplementary Figures**


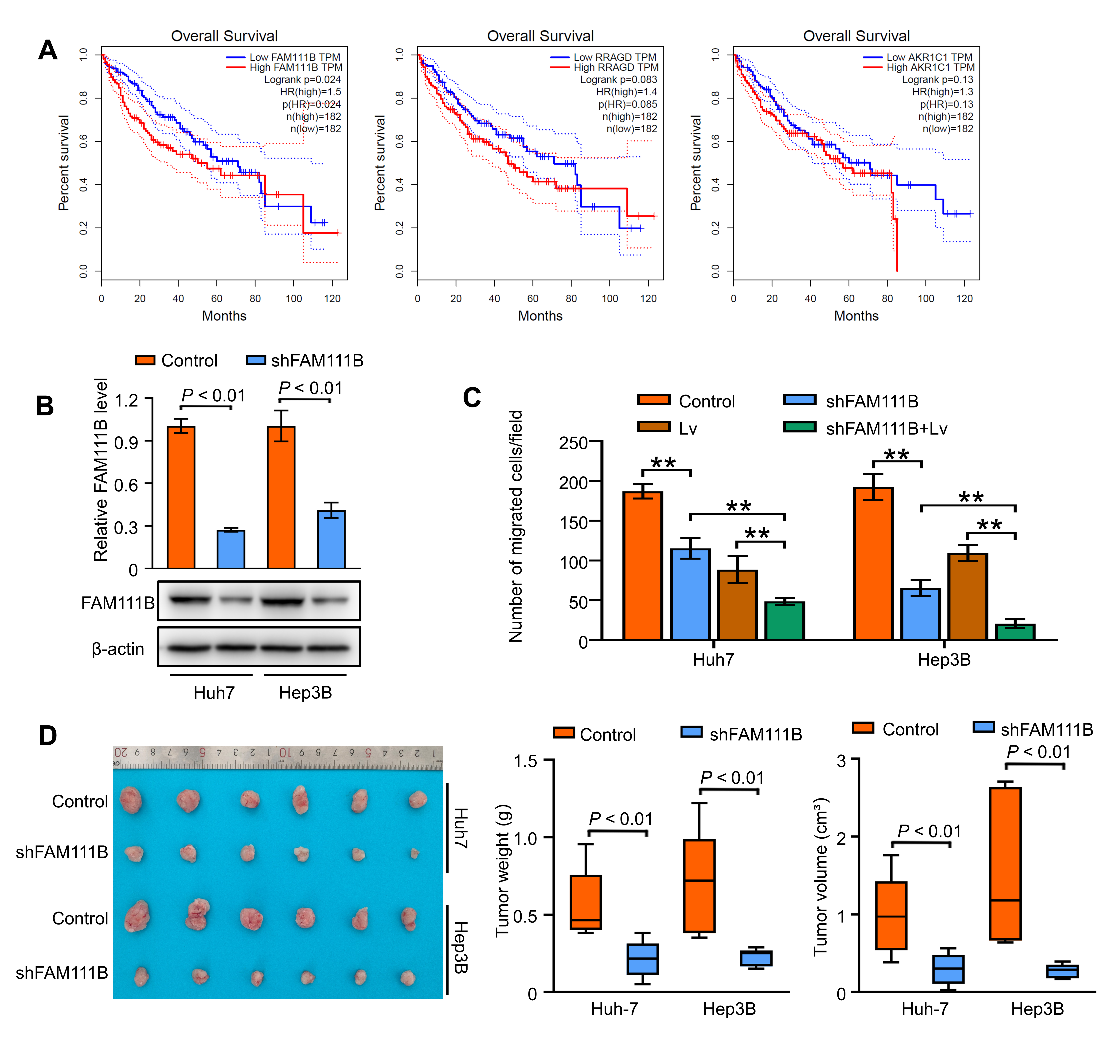


**Figure S1.** (A) Kaplan–Meier analysis showed the overall survival of three common genes in the TCGA-LIHC database. (B) The knockdown efficiency of the shFAM111B sequence was assessed by RT‒PCR and western blotting. (C) Transwell assays showed that knockdown FAM111B inhibited the migration ability of HCC cells. (D) Endpoint tumour images of the CDX models derived from shFAM111B or control HCC cells, as well as the statistics analysis of tumour weight and volume (n = 6 per group).


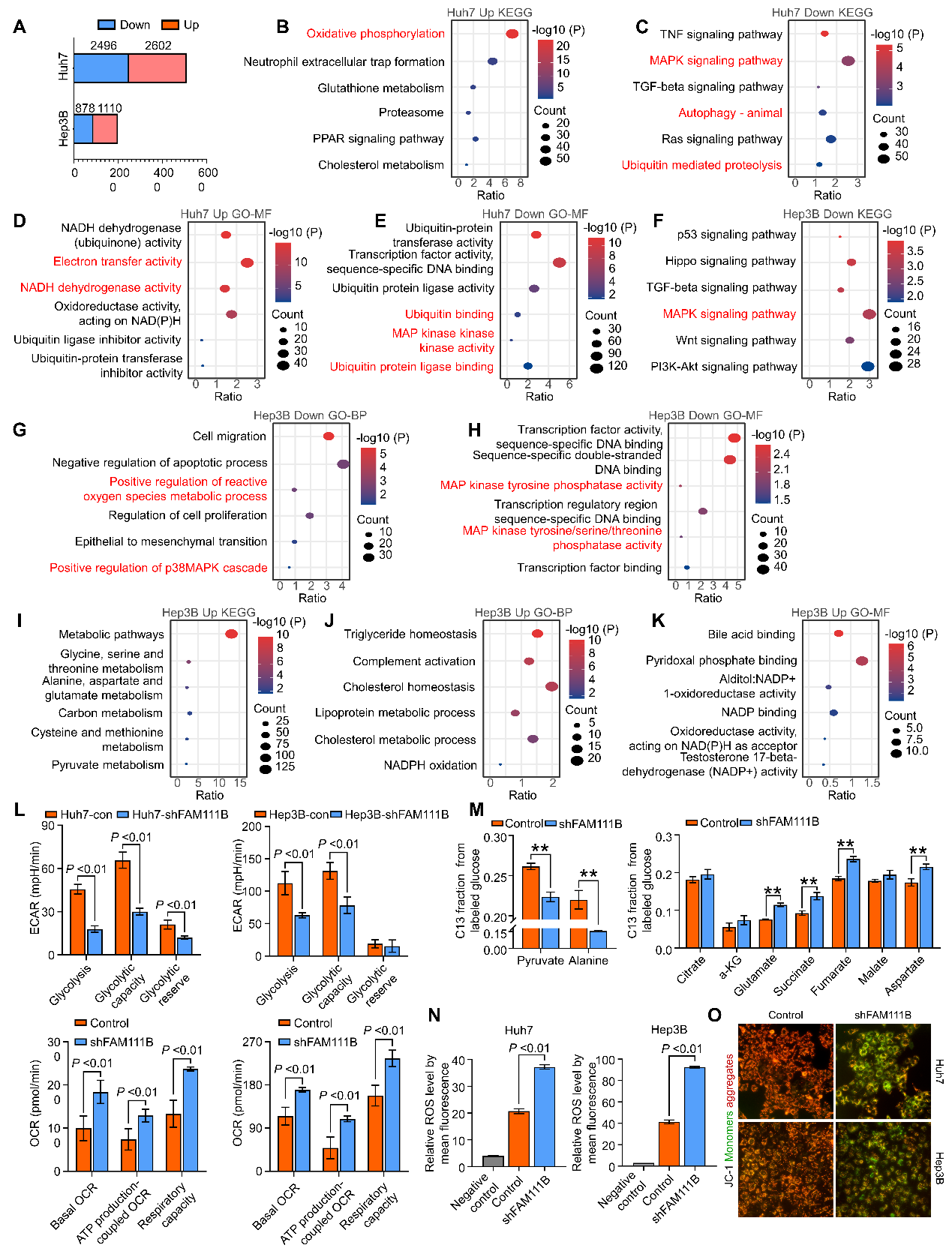


**Figure S2.** (A) DEGs were identified via RNA-seq analysis comparing control and Huh7/Hep3B shFAM111B cells. (B-K) Upregulated and downregulated gene set enrichment analysis showing the signalling pathways and biological processes that were correlated with FAM111B knockdown. (L) Statistical analysis of the ECAR and OCR in control and shFAM111B HCC cells. (M) Statistical analysis of the C^13^ incorporation ratio of metabolites in glycolysis and OXPHOS, ***p* < 0.01. (N) Statistical analysis of flow cytometry data for ROS detection. (O) A representative image showing the level of the mitochondrial membrane potential as measured with the JC-1 probe.


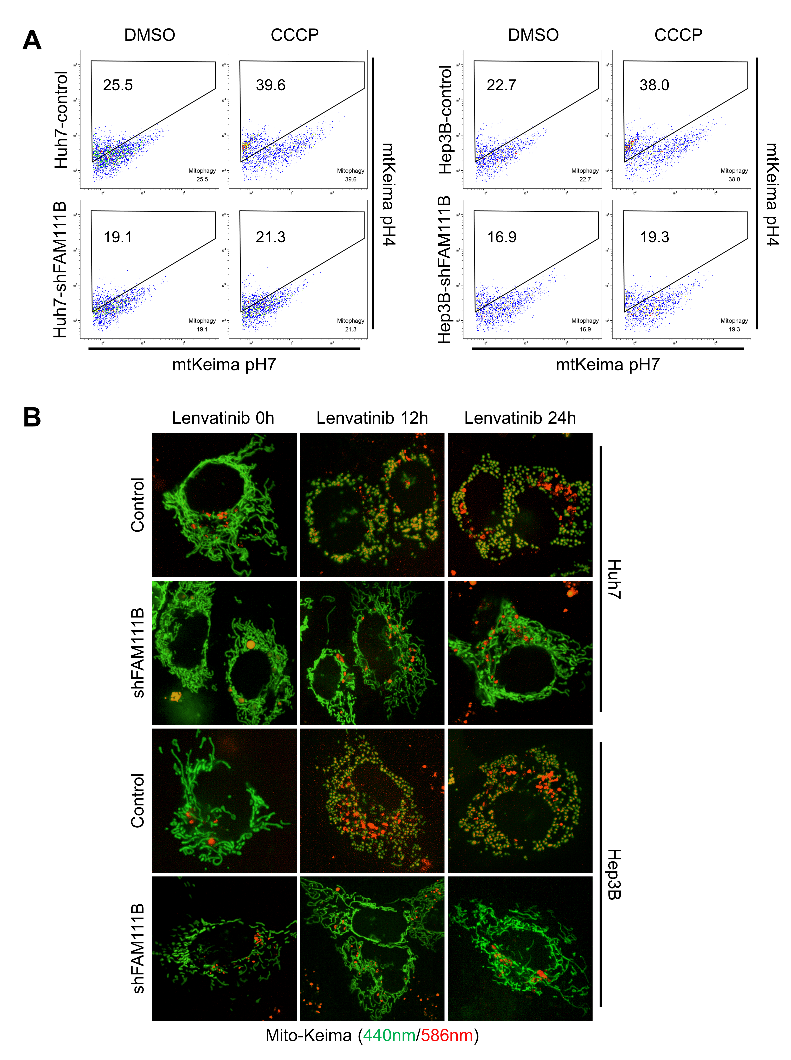


**Figure S3.** (A) Flow cytometry was used to determine the ratio of acidified mitochondria in control and shFAM111B HCC cells. (B) Representative confocal images showing acidified (red) and unacidified (green) mitochondria in control and shFAM111B HCC cells treated with lenvatinib.


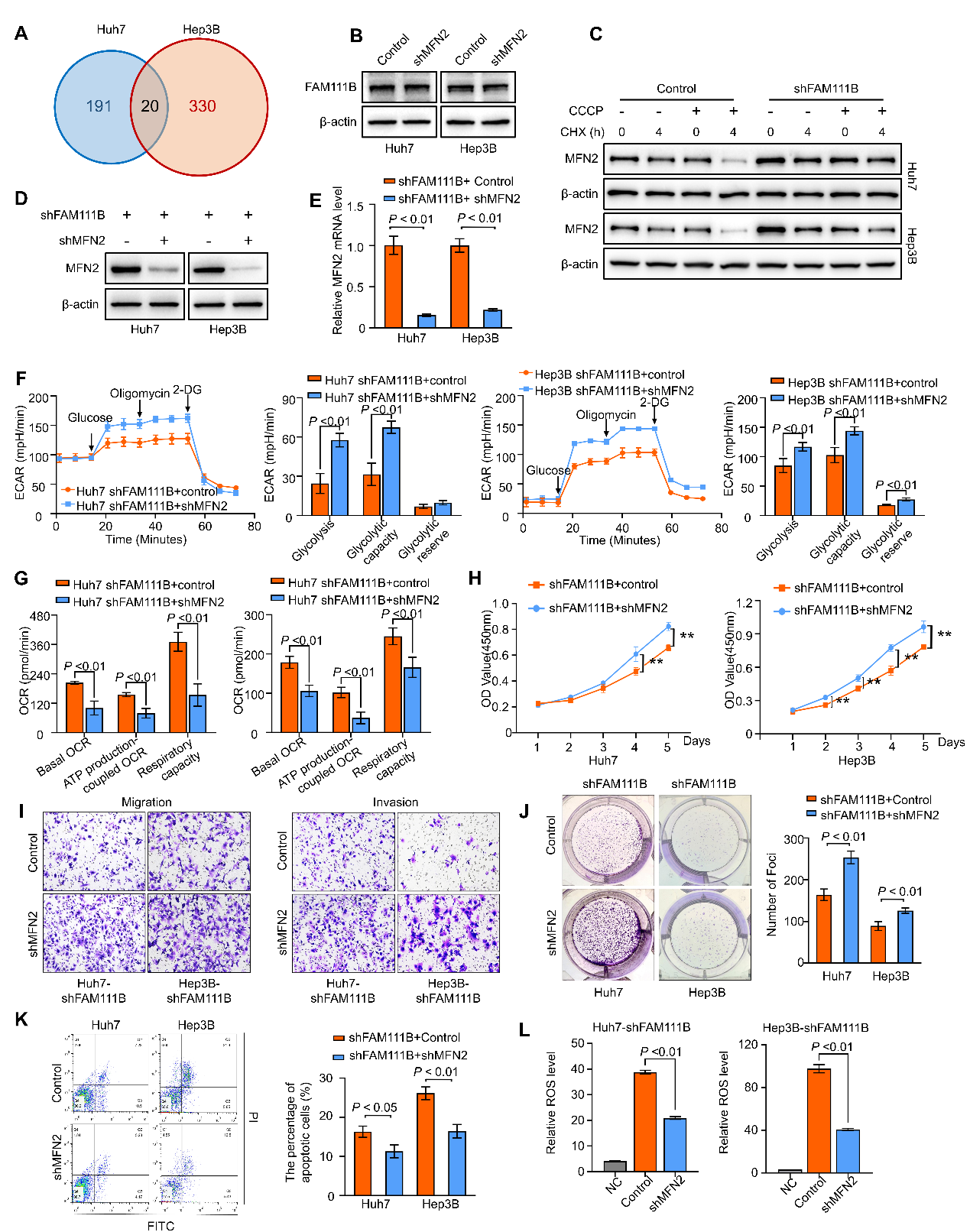


**Figure S4.** (A) Venn diagram showing the results of the co-IP assay and 2D-LS/MS analysis. (B) Western blot showing that MFN2 knockdown had no effect on FAM111B expression. (C) CHX chase analysis revealed that FAM111B knockdown significantly suppressed MFN2 degradation under CCCP (10 μM) stimulation. (D, E) Western blot and qPCR results confirmed the efficiency of MFN2 knockdown. (F, G) Seahorse assays and statistical analysis of the ECAR and OCR in FAM111B-KD+MFN2-KD HCC cells. (H) MFN2 knockdown notably reversed the effects of FAM111B knockdown on cell proliferation, migration, invasion (I), colony formation (J) and apoptosis (K) in Huh7 and Hep3B cells. (L) Statistical analysis of the mitochondrial ROS levels.


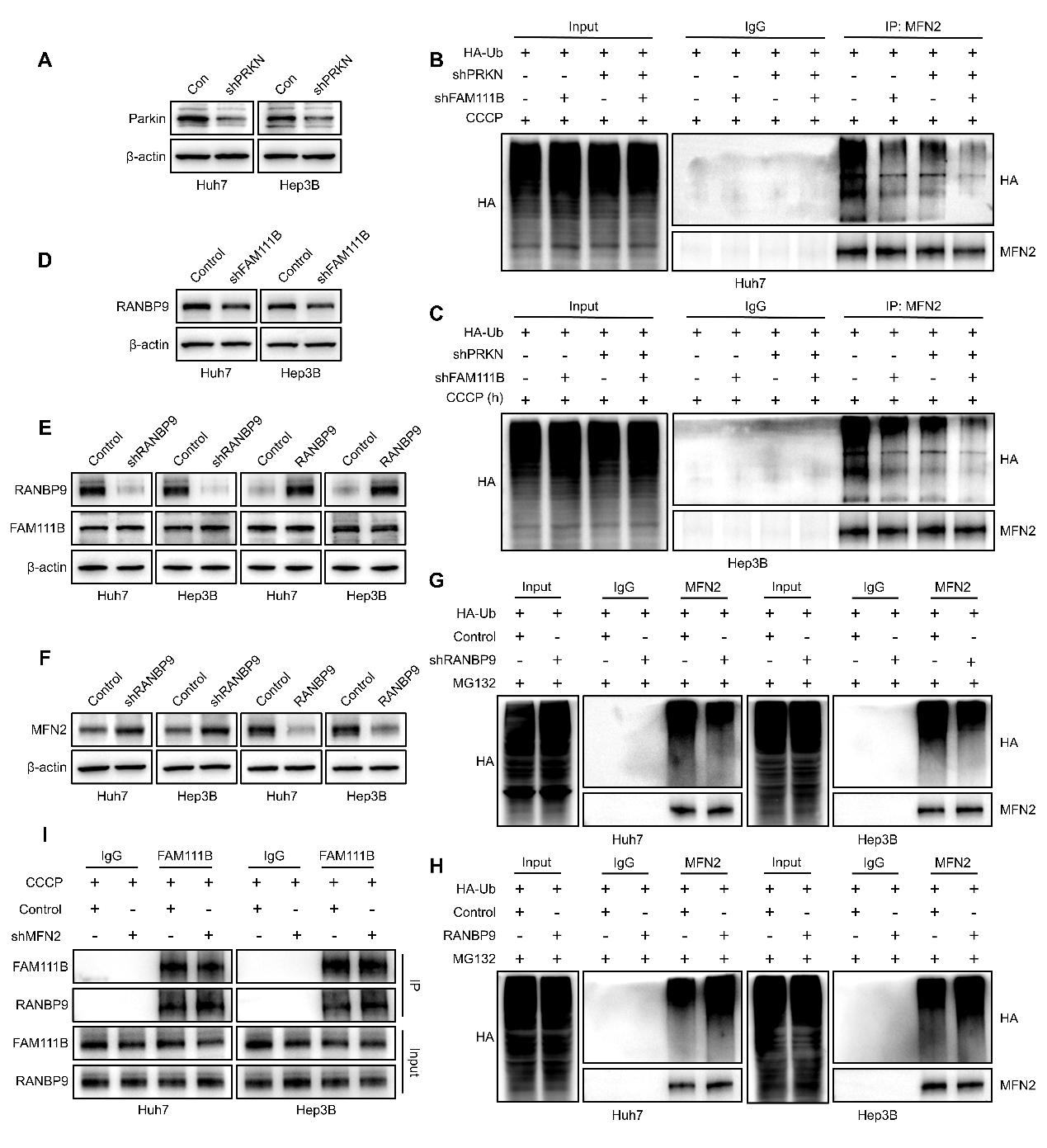


**Figure S5.** (A) Western blot assays were used to assess the Parkin-knockdown efficiency. (B, C) Co-IP results revealed that FAM111B knockdown further decreased MFN2 ubiquitination after Parkin knockdown in Huh7 and Hep3B cells. (D) Western blot results showing the effect of FAM111B knockdown on RANBP9 expression. (E) Western blot assays were used to assess the RANBP9 knockdown and overexpression efficiency and the effect of RANBP9 knockdown and overexpression on FAM111B. (F-H) Western blot and co-IP assay results indicated that RANBP9 knockdown and overexpression suppressed and enhanced the ubiquitin-mediated degradation of MFN2, respectively. (I) Co-IP results indicated that knocking down MFN2 did not interfere with the FAM111B-RANBP9 interaction.


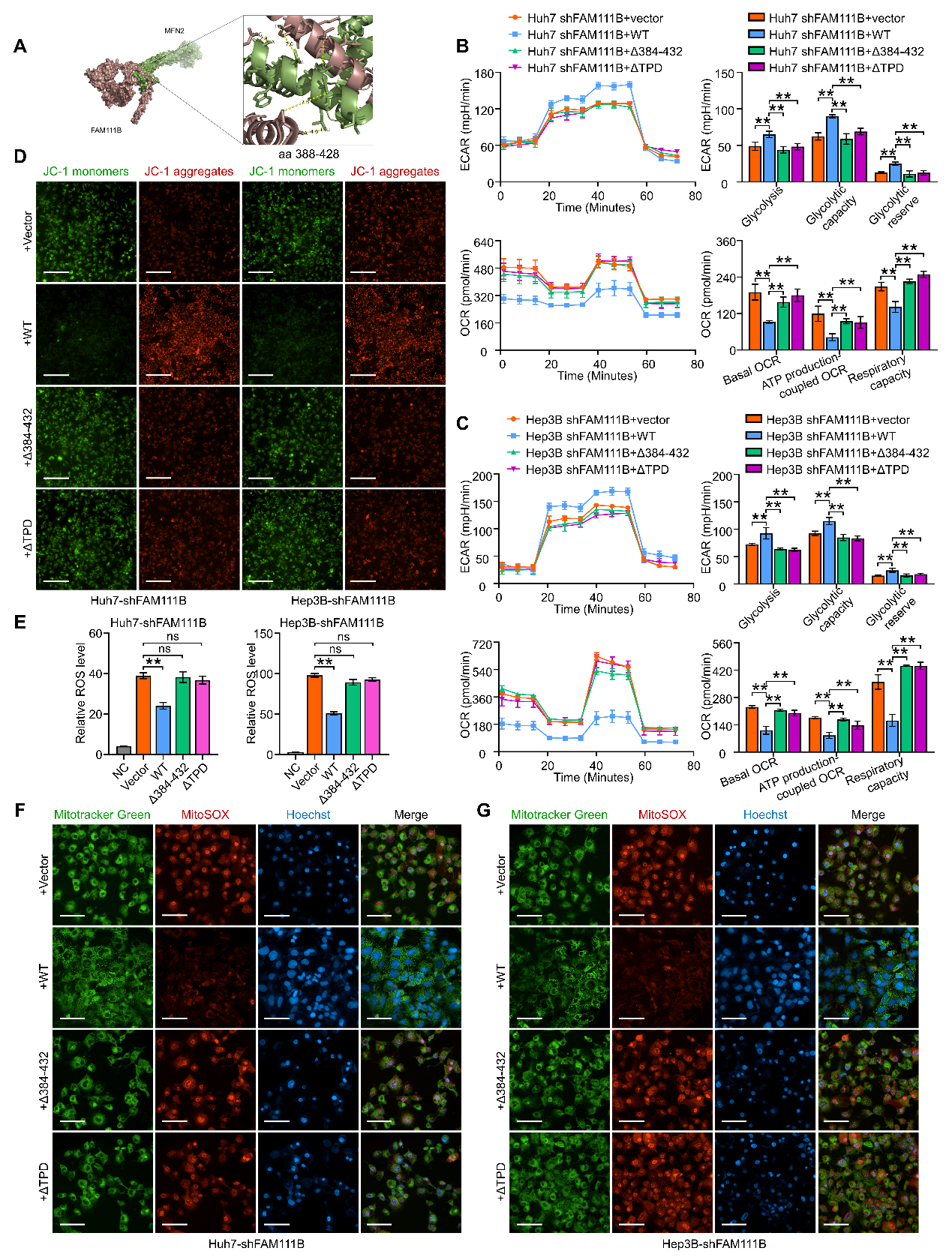


**Figure S6.** (A) Diagram showing the molecular docking results. (B, C) Seahorse assays were used to assess the ECAR and OCR in the abovementioned groups. (D) Representative images showing the effect of exogenous wild-type or mutant FAM111B on the mitochondrial membrane potential in FAM111B-KD cells. (E) Statistical analysis of the mitochondrial ROS levels, as determined by flow cytometry. (F, G) Representative images showing the level of mitochondrial ROS in the indicated groups.


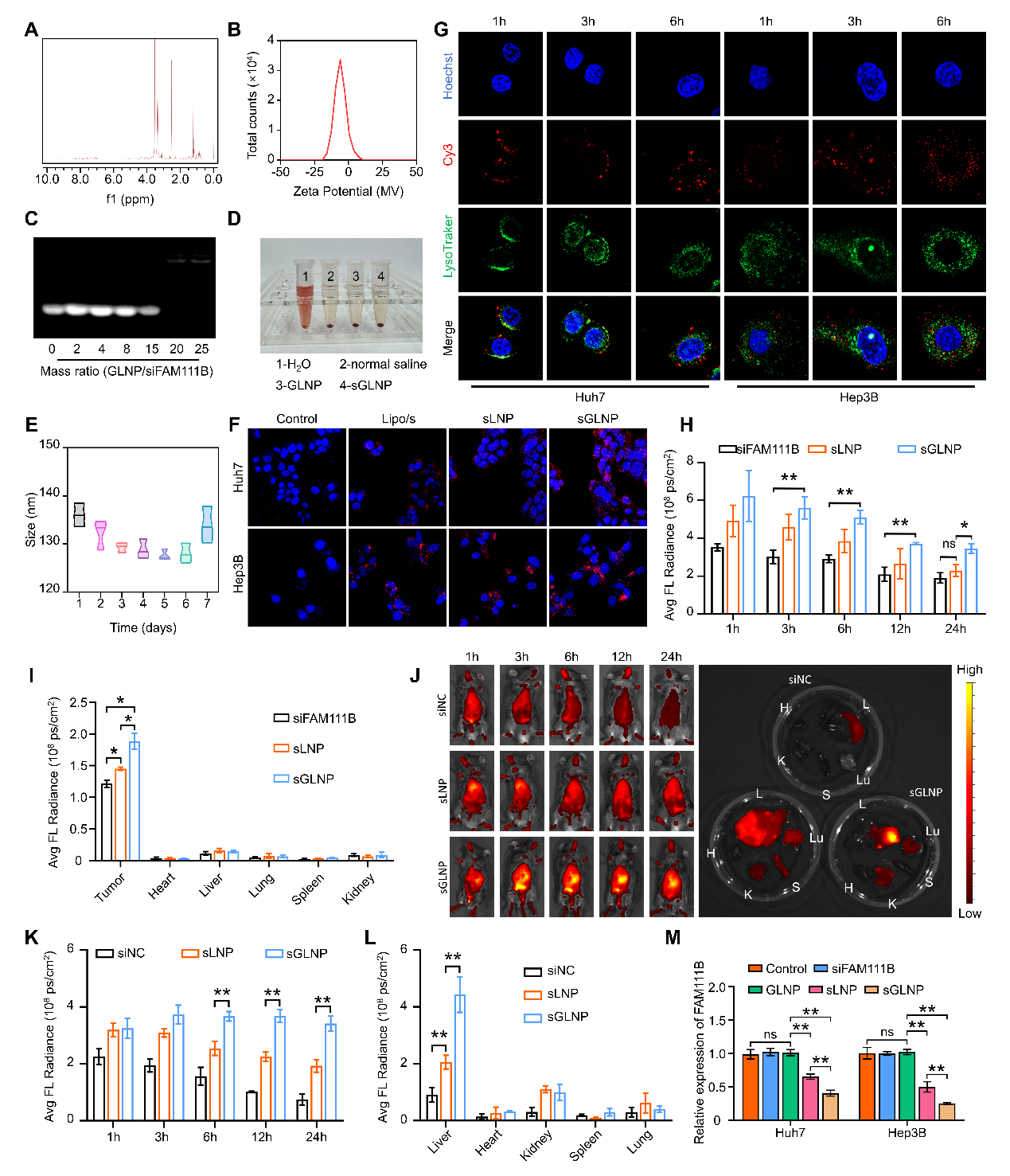


**Figure S7. GPC3-targeted LNP carrying siFAM111B sequence precisely blocked HCC progression**

(A) ^1^H NMR spectrum confirming the successful synthesis of DSPE-PEG-GPC3. (B) Zeta potential of sGLNP. (C) Agarose gel retardation assays demonstrating complete siRNA encapsulation by GLNP at mass ratio ≥ 20:1. (D) Representative image and statistical analysis of red blood cell haemolysis assay. (E) Stability of sGLNP in PBS. (F) Confocal images showing intracellular uptake of free siRNA, Lipo3000 reagent, sLNP or sGLNP in Huh7 and Hep3B cells. (G) Time-dependent subcellular localization of sGLNP Huh7 and Hep3B cells at 1, 3, and 6 h. (H) Statistical analysis of the in vivo fluorescence distribution, **p* < 0.05, ***p* < 0.01. (I) Quantification of Cy5.5-tagged free siRNA, sLNP and sGLNP in main organs isolated from BALB/c nude mice, **p* < 0.05. (J) In vivo fluorescence distribution of Cy5.5-tagged free siRNA, sLNP and sGLNP in orthotopic HCC models after i.v. injection. Abbreviation: L for liver, H for heart, K for kidney, S for spleen, Lu for lung. (K, L) Statistical analysis of the biodistribution of Cy5.5-tagged free siRNA, sLNP and sGLNP in orthotopic models, ***p* < 0.01. (M) RT‒PCR analysis of FAM111B knockdown efficiency in HCC cells treated with sGLNP and sLNP, ***p* < 0.01.

**Supplementary Table S1 Univariate and multivariate analysis of OS in HCC patients**

| Variable | Univariate analysis | | Multivariate analysis | |
| --- | --- | --- | --- | --- |
|  | X^2^ P-value | | (95%Cl) P-value | |
| FAM111B level (high vs low) | 22.488 | <0.001 | (1.363~11.295) | <0.001 |
| Gender (male vs female)) | 2.240 | 0.136 | (0.398~1.639) | 0.553 |
| Age (years) (≤50 vs >50) | 0.937 | 0.334 | (0.910~2.259) | 0.121 |
| Tumour diameter (cm) (≤5  vs >5) | 0.496 | 0.482 | (0.551~1.370) | 0.545 |
| Vascular invasion (yes vs no) | 0.111 | 0.739 | (0.944~11.203) | 0.062 |
| Tumour capsule (none vs yes) | 0.060 | 0.807 | (0.567~1.387) | 0.598 |
| Tumour number (≥2 vs <2cm) | 21.572 | <0.001 | (2.152~5.718) | <0.001 |
| AFP (ng/ml) (≤400 vs >400) | 2.021 | 0.157 | (0.922~2.368) | 0.104 |
| Tumour differentiation (III vs I-II) | 2.595 | 0.109 | (0.410~1.074) | 0.095 |
| Liver cirrhosis (yes vs no) | 8.107 | 0.005 | (1.033~4.246) | 0.040 |
| HBsAg (positive v negative) | 2.148 | 0.144 | (0.761~2.671) | 0.268 |

**Supplementary Table S2 Univariate and multivariate analysis of RFS in HCC patients**

| Variable | Univariate analysis | | Multivariate analysis | |
| --- | --- | --- | --- | --- |
|  | X^2^ P-value | | (95%Cl) P-value | |
| FAM111B level (high vs low) | 20. 550 | <0.001 | (1.82~8.823) | <0.001 |
| Gender (male vs female)) | 0.326 | 0.569 | (0.554~1.834) | 0.978 |
| Age (years) (≤50 vs >50) | 0.005 | 0.941 | (0.631~1.530) | 0.937 |
| Tumour diameter (cm) (≤5  vs >5) | 1.241 | 0.267 | (0.478~1.137) | 0.167 |
| Vascular invasion (yes vs no) | 1.876 | 0.172 | (1.470~13.225) | 0.008 |
| Tumour capsule (none vs yes) | 0.040 | 0.842 | (0.622~1.439) | 0.795 |
| Tumour number (≥2 vs <2cm) | 8.611 | 0.004 | (1.661~4.462) | <0.001 |
| AFP (ng/ml) (≤400 vs >400) | 0.145 | 0.703 | (0.709~1.666) | 0.701 |
| Tumour differentiation (III vs I-II) | 1.679 | 0.197 | (0.451~1.103) | 0.126 |
| Liver cirrhosis (yes vs no) | 2.375 | 0.125 | (0.788~2.425) | 0.258 |
| HBsAg (positive v negative) | 0.020 | 0.886 | (0.483~1.440) | 0.515 |

**Supplementary Table S3 Statistics for FAM111B and clinicopathologic features in HCC patients**

| Clinicopathological  feature | FAM111B Expression | | |  |  |
| --- | --- | --- | --- | --- | --- |
|  | Total Low High | | | X^2^ *P*-Value | |
|  | 100 | 49 | 51 |  |  |
| Age (years) |  |  |  | 0.749 0.387 | |
| ≤50 | 61 | 32 | 29 |  |  |
| >50 | 39 | 17 | 22 |  |  |
| Gender |  |  |  | 0.538 0.463 | |
| Male | 90 | 43 | 47 |  |  |
| Female | 10 | 6 | 4 |  |  |
| Tumour diameter |  |  |  | 11.76 <0.001 | |
| ≤5cm | 60 | 21 | 39 |  |  |
| >5cm | 40 | 28 | 12 |  |  |
| Tumour invasion |  |  |  | 21.13 <0.001 | |
| No | 48 | 35 | 13 |  |  |
| Yes | 52 | 14 | 38 |  |  |
| Tumour capsule |  |  |  | 5.841 0.016 | |
| Yes | 47 | 17 | 30 |  |  |
| None | 53 | 32 | 21 |  |  |
| Tumour number |  |  |  | 27.57 <0.001 | |
| 1 | 41 | 33 | 8 |  |  |
| ≥2 | 69 | 16 | 43 |  |  |
| AFP |  |  |  | 0.132 0.717 | |
| ≤400ng/ml | 47 | 20 | 27 |  |  |
| >400ng/ml | 63 | 29 | 34 |  |  |
| Tumour diffierentitation |  |  |  | 6.732 0.01 | |
| I-II | 48 | 30 | 18 |  |  |
| III | 52 | 19 | 33 |  |  |
| Liver cirrhosis |  |  |  | 0.036 0.847 | |
| None | 46 | 20 | 26 |  |  |
| Yes | 54 | 29 | 35 |  |  |
| HBsAg |  |  |  | 4.123 0.042 | |
| Negative | 45 | 17 | 28 |  |  |
| Positive | 55 | 32 | 23 |  |  |

**Supplementary Table S4 List of primer sequences**

| Gene | Forward primer sequence | Reverse primer sequence |
| --- | --- | --- |
| β-actin | GAAGAGCTACGAGCTGCCTGA | CAGACAGCACTGTGTTGGCG |
| FAM111B | AACTCGCCAGACAATTCCCA | GCATACCGCCTACCCAGATG |
| MFN2 | GCGGTTCGACTCATCATGGA | TCACGCATTTCCTCGCAGTA |

**Supplementary Table S5 List of primary antibodies**

| Antibody name | Corporation | Application |
| --- | --- | --- |
| Rabbit anti-FAM111B | Thermo Fisher Technology | WB: 1:1000, IP: 1:50, IHC/IF: 1:100 |
| Rabbit anti-MFN2 | Cell Signaling Technology | WB: 1:1000, IP: 1:50, IHC: 1:200 |
| Mouse anti-MFN2 | Abcam | WB: 1:1000, IF: 1:200 |
| Rabbit anti-LC3 | Abcam | WB: 1:2000, IF: 1:200 |
| Rabbit anti-SQSTM1 | Abcam | WB: 1:10000 |
| Mouse anti-COX IV | Cell Signaling Technology | IF: 1:200 |
| Rabbit anti-LAMP1 | Cell Signaling Technology | IF: 1:200 |
| Rabbit anti-RANBP9 | Abcam | WB: 1:1000, IHC: 1:100 |
| Rabbit anti-PRKN | Cell Signaling Technology | WB: 1:1000 |
| Rabbit anti-HA-Tag | Cell Signaling Technology | IP: 1:50 |
| Mouse anti-HA-Tag | Cell Signaling Technology | WB: 1:1000 |
| Mouse anti-MYC-Tag | Cell Signaling Technology | WB: 1:1000 |
| Mouse anti-Flag-Tag | Cell Signaling Technology | WB: 1:1000 |
| Mouse anti-β-actin | HUABIO | WB:1: 10000 |

**Supplementary Table S6 List of transfection sequences**

| Gene | Transfection sequences |
| --- | --- |
| shFAM111B | CTGCCTAGTGATTCTCATT |
| shMFN2 | AGGTTTACTGCGAGGAAAT |
| shPRKN | GAGCTCCATCACTTCAGGATT |
| shRANBP9 | GAGCTCCATCACTTCAGGATT |
