# Supplementary material for: Targeting FAM111B attenuates mitophagy and increases the sensitivity to lenvatinib treatment by increasing MFN2 stability in hepatocellular carcinoma
Source: Cell Death Dis. 2025 Aug 25;16(1):645. doi: 10.1038/s41419-025-07941-1 (PMC12379274; doi:10.1038/s41419-025-07941-1)
Supplement: Supplementary file 2 — Western blot data [file 41419_2025_7941_MOESM2_ESM.pdf]

Fig. S1A

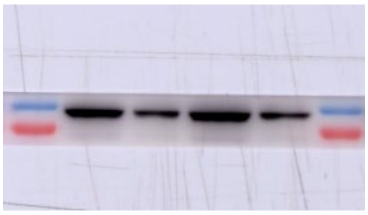

FAM111B 90kd

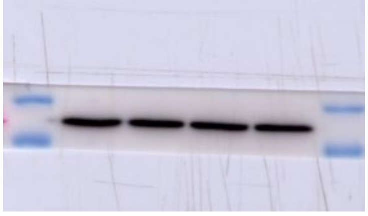

Actin 42kd

Fig. 3C

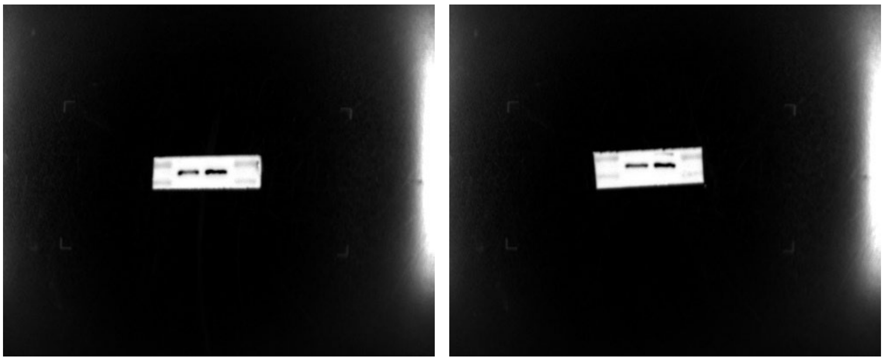

p62 62kd

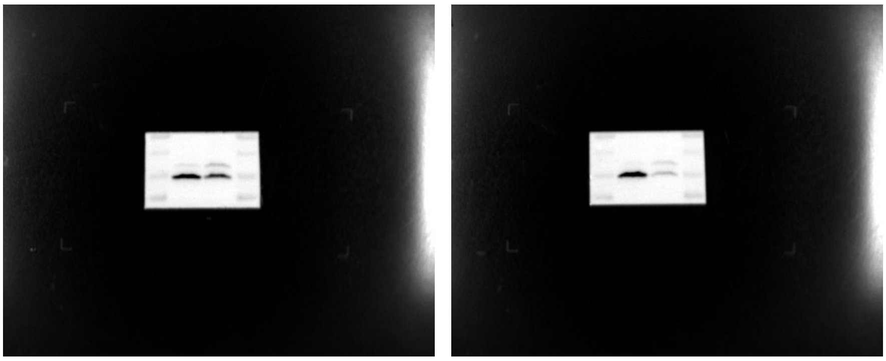

LC3 15kd

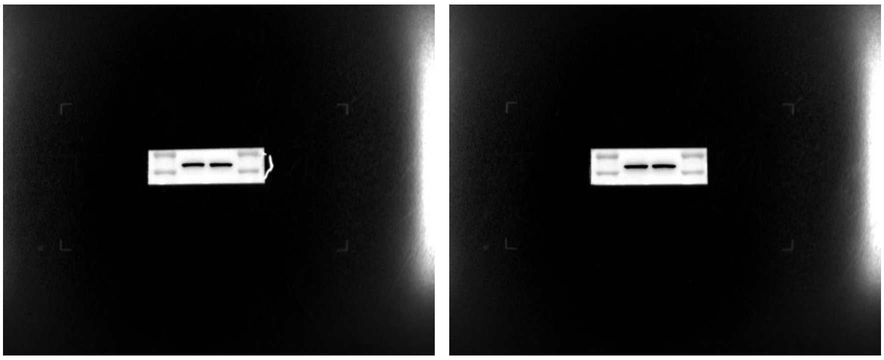

Actin 42kd

Fig. 4C left

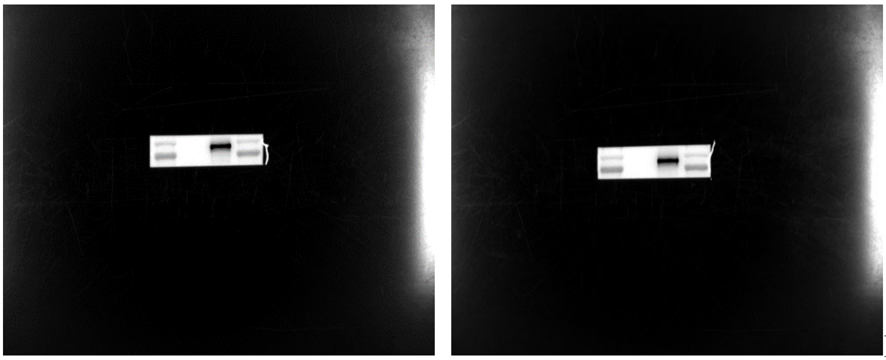
MFN2 80kd

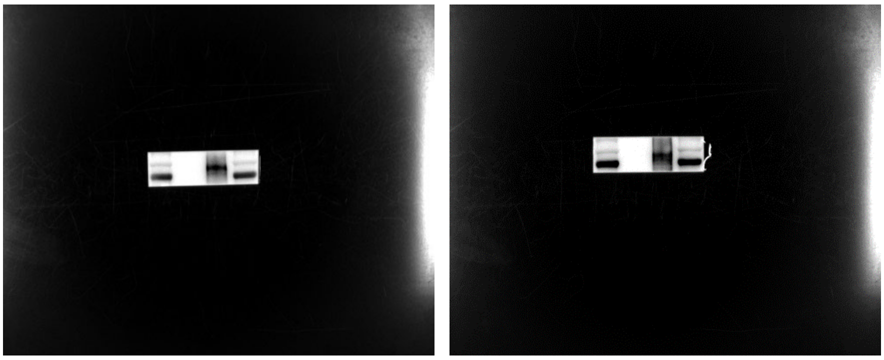
FAM111B 90kd

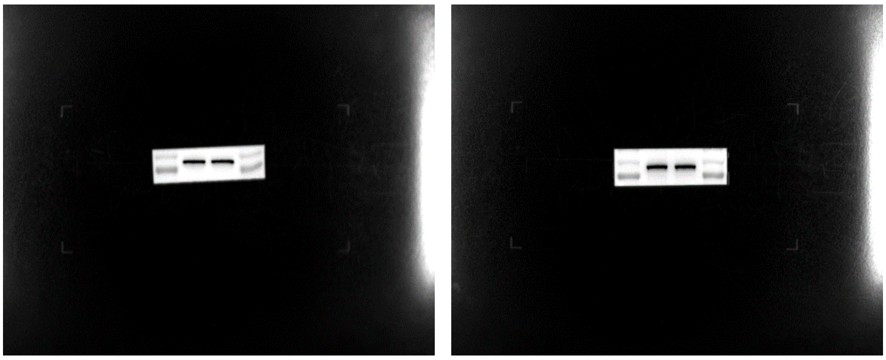
MFN2 80kd

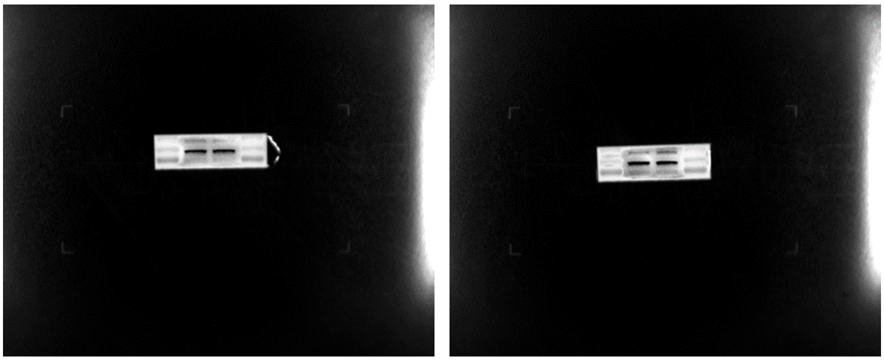
FAM111B 90kd

Fig. 4C right

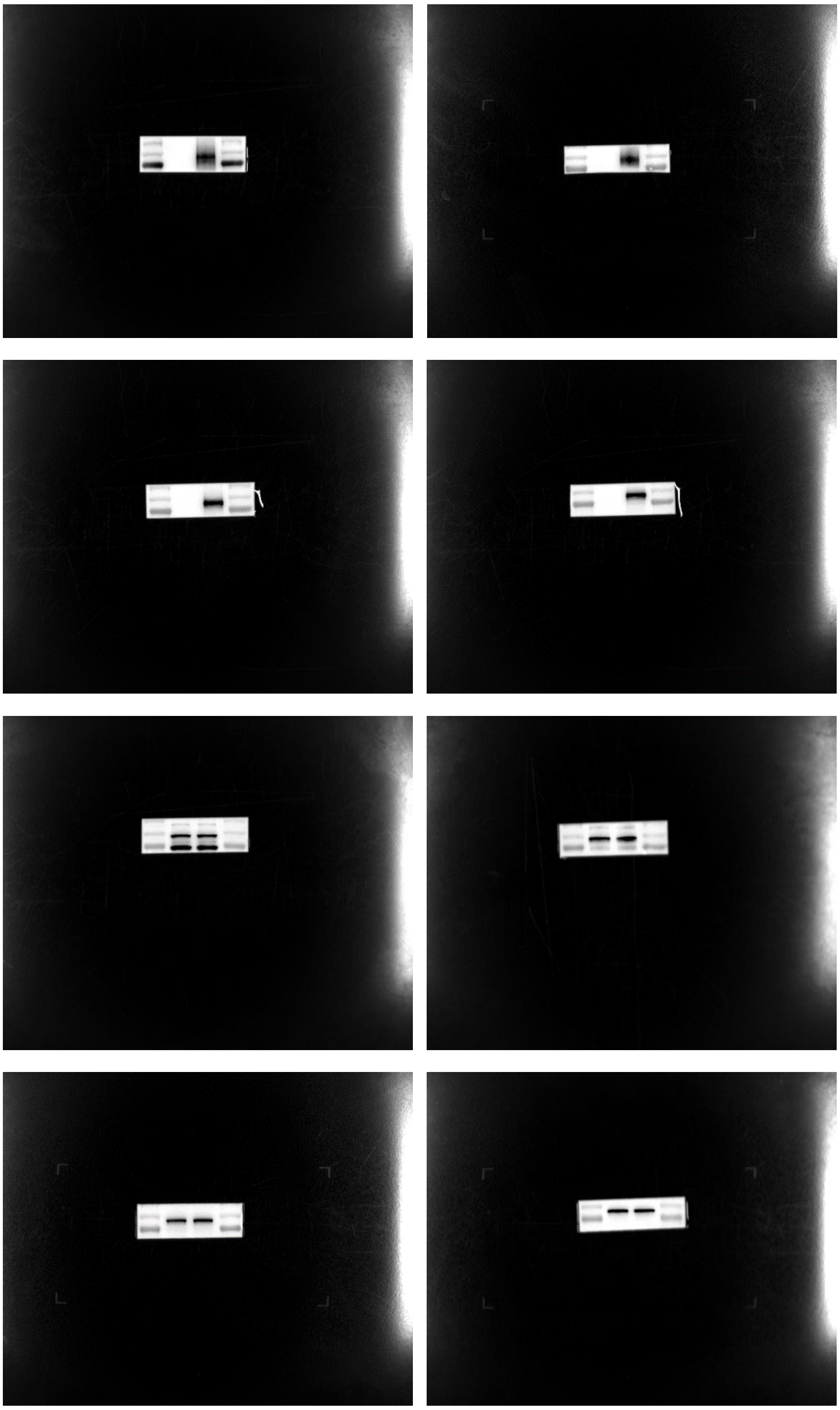

FAM111B 90kd

MFN2 80kd

FAM111B 90kd

MFN2 80kd

Fig. 4D

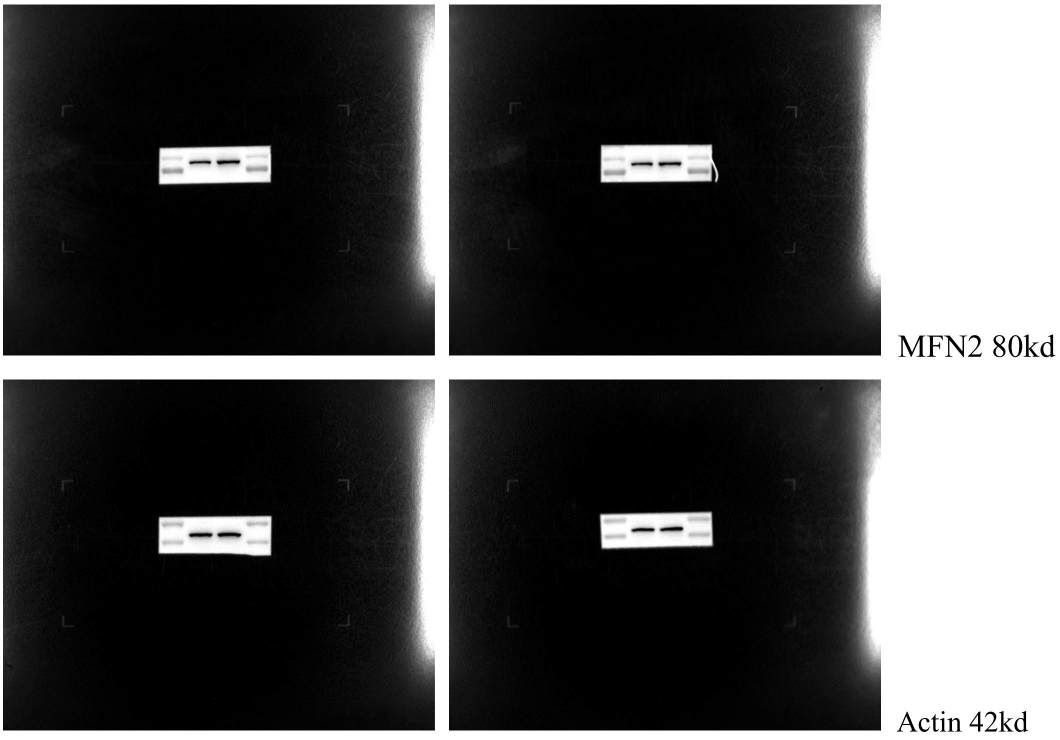

Fig. 4F

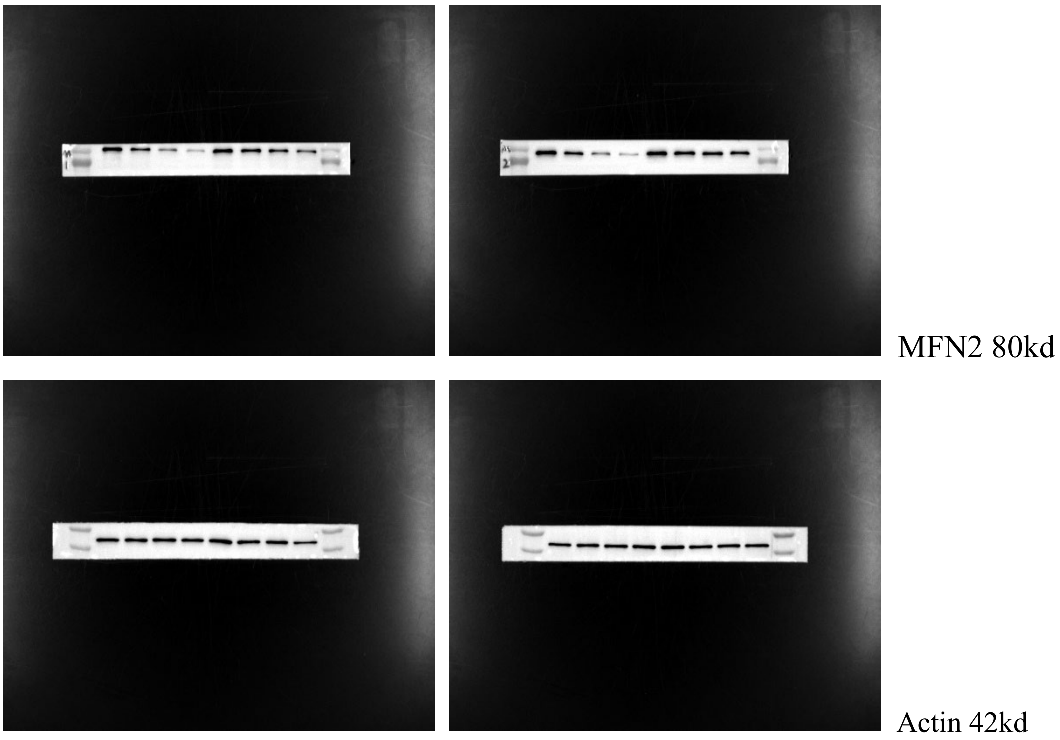

Fig. 4I

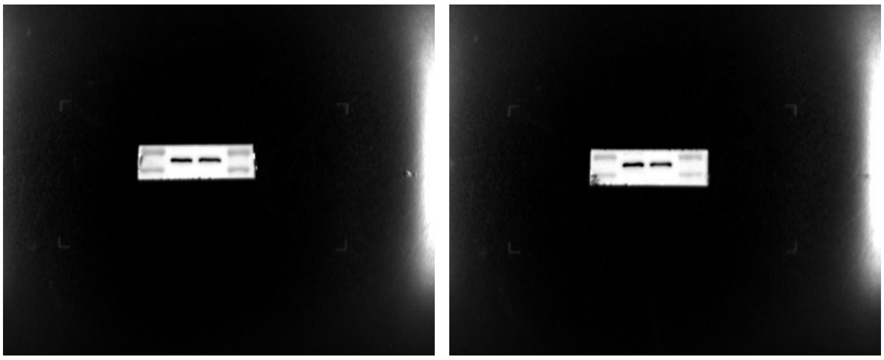

p62 62kd

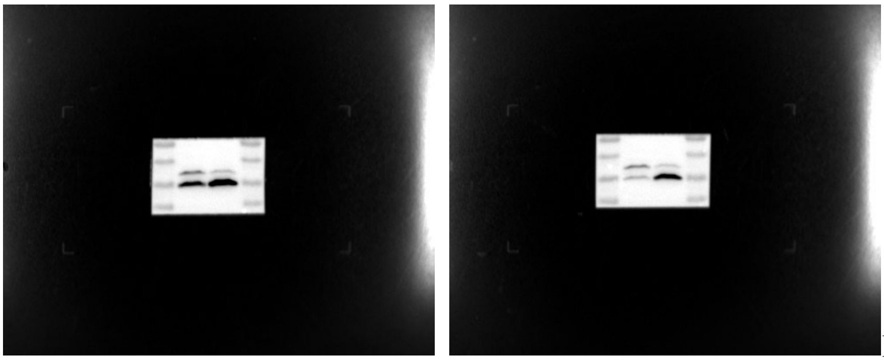

LC3 15kd

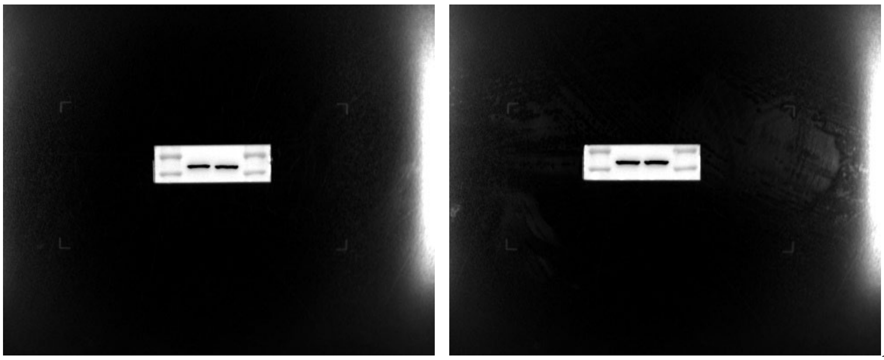

Actin 42kd

Fig. S4B

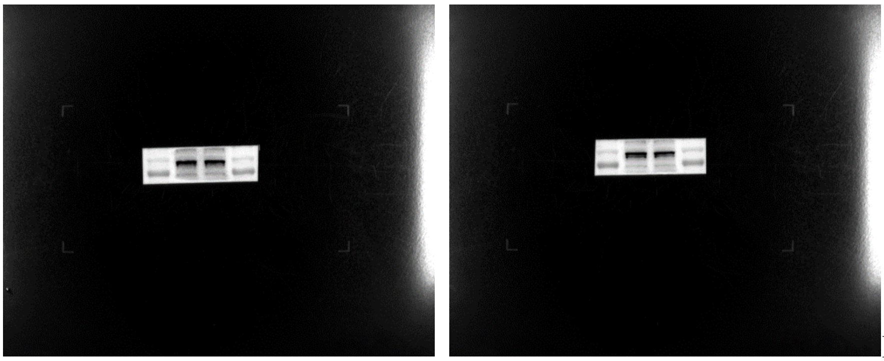

FAM111B 90kd

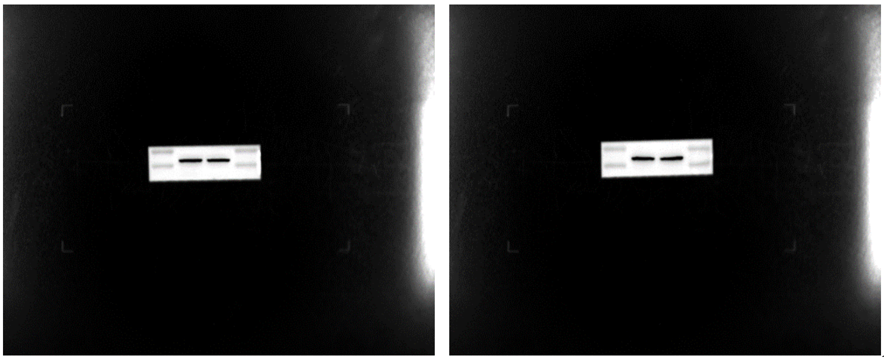

Actin 42kd

Fig. S4C

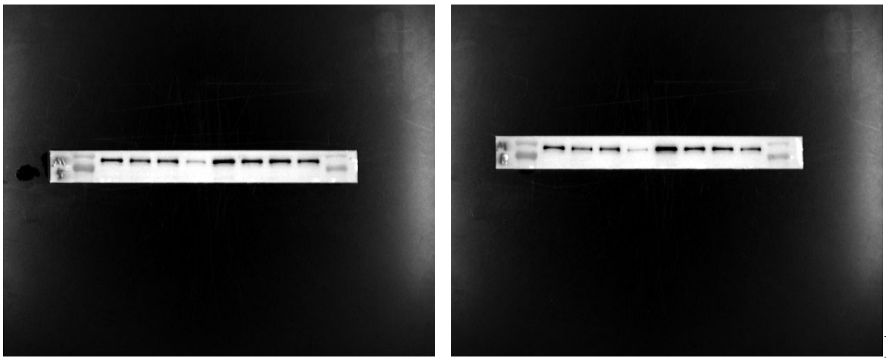

MFN2 80kd

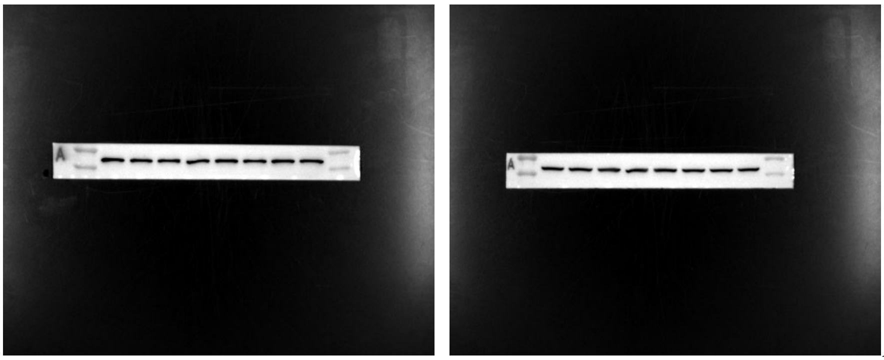

Actin 42kd

Fig. S4D

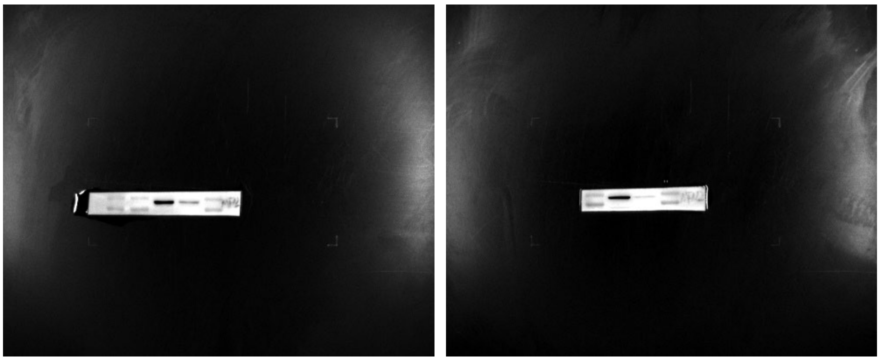

MFN2 80kd

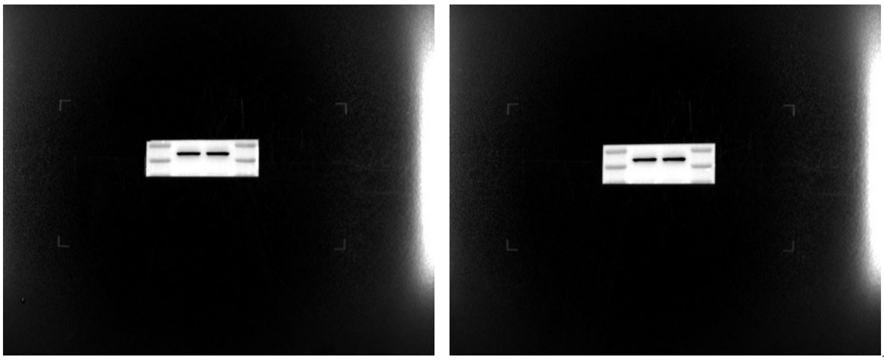

Actin 42kd

Fig. 5A

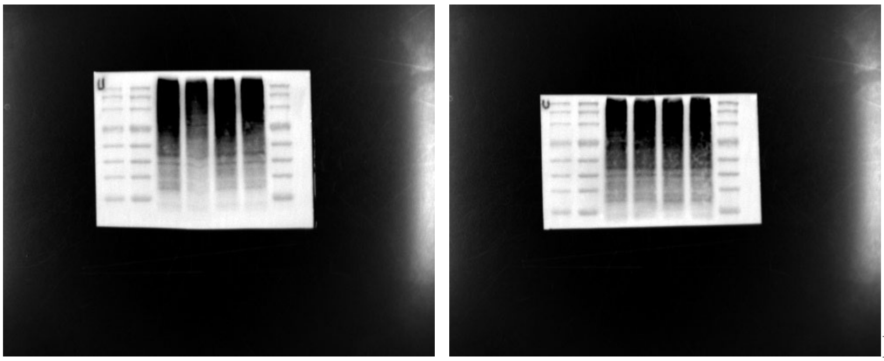

Input-Ub

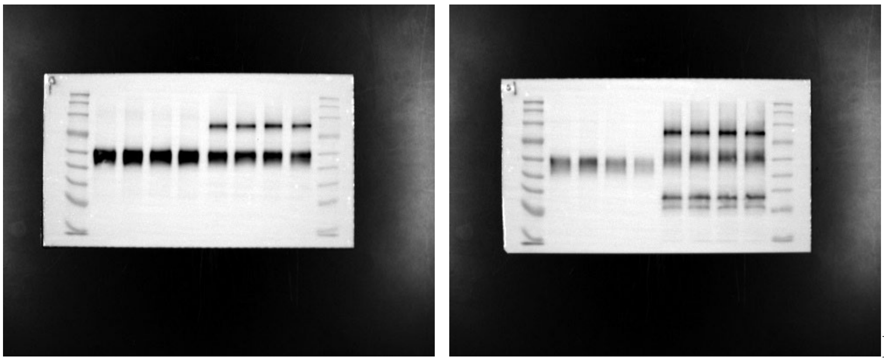

MFN2 80kd

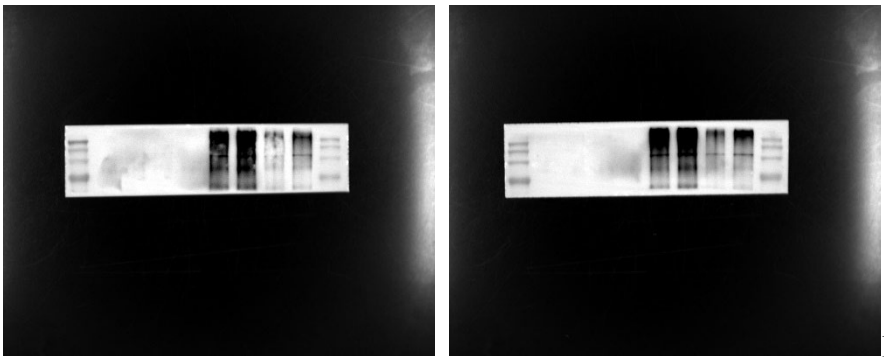

IP-Ub

Fig. 5B

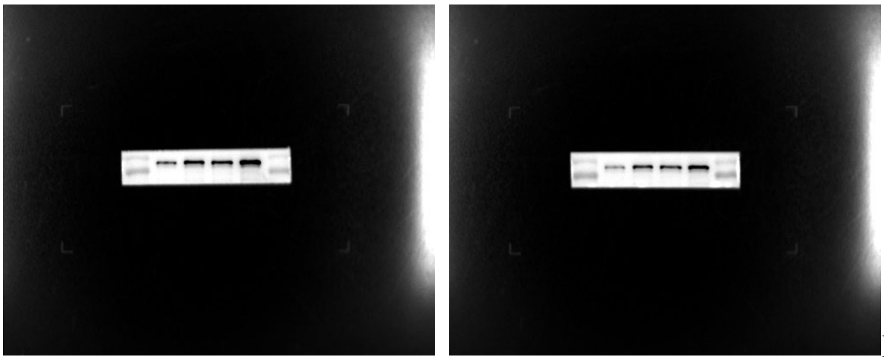

MFN2 80kd

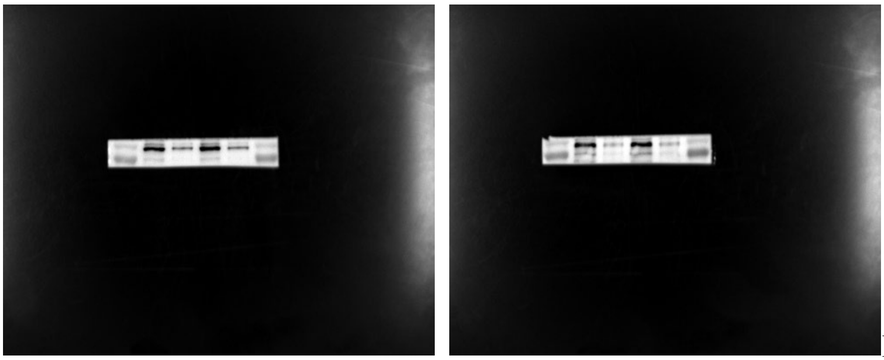

FAM111B 90kd

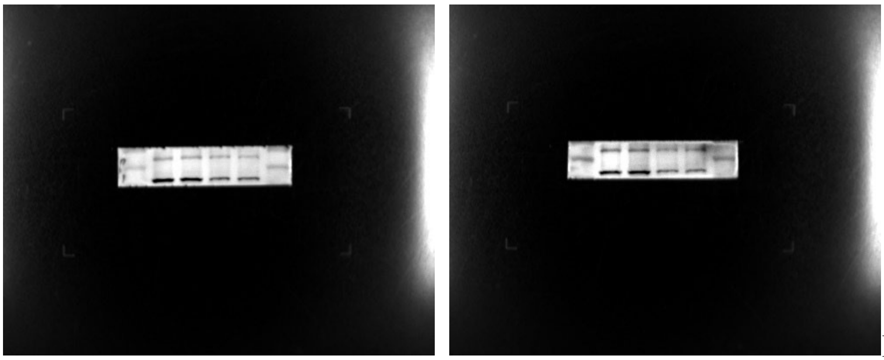

Parkin 50kd

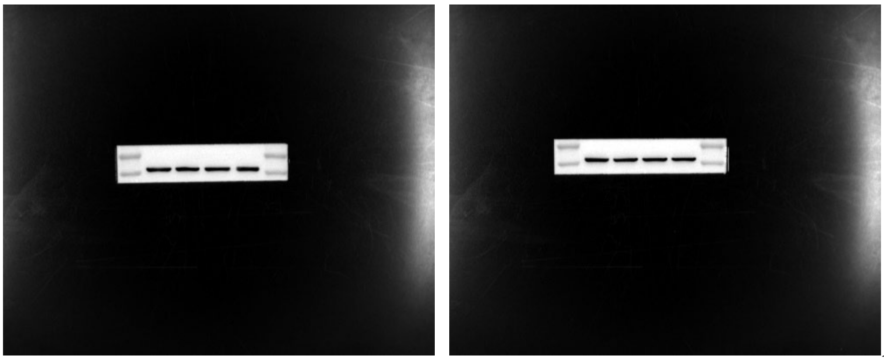

Actin 42kd

Fig. 5C left

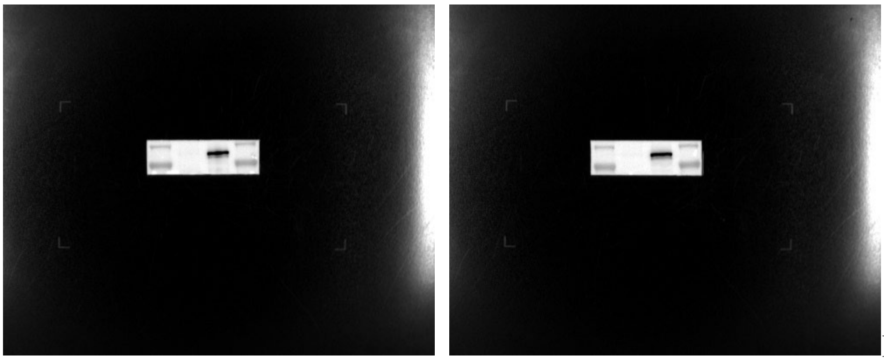
MFN2 80kd

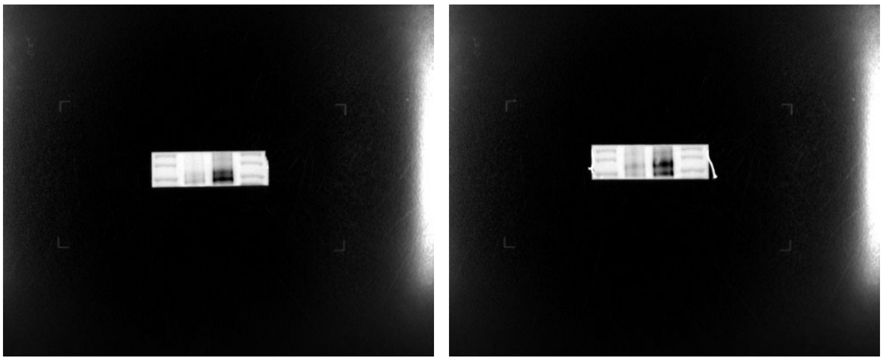
RANBP9 116kd

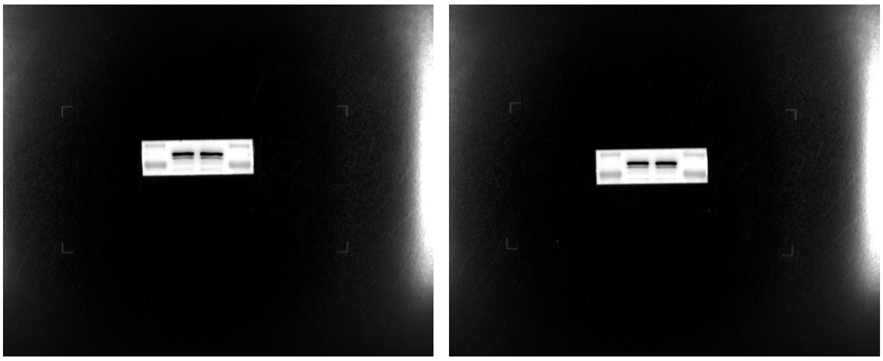
MFN2 80kd

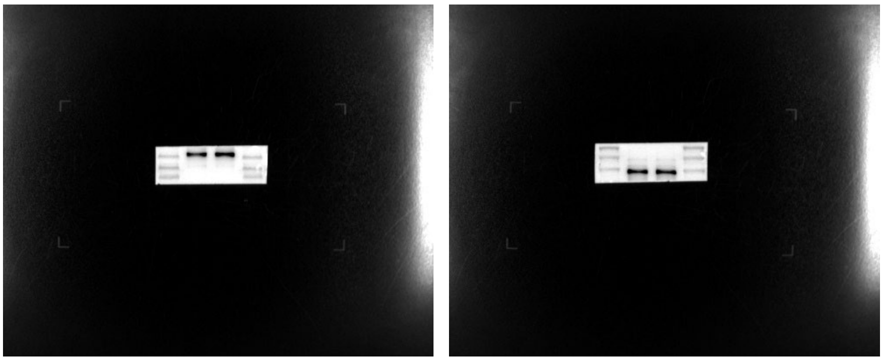
RANBP9 116kd

Fig. 5C right

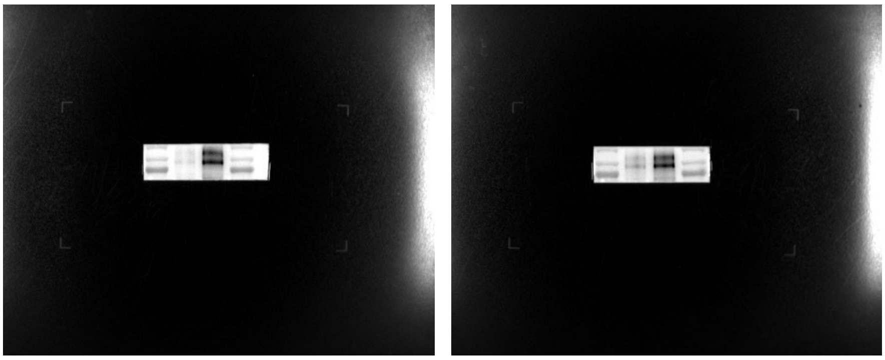

FAM111B 90kd

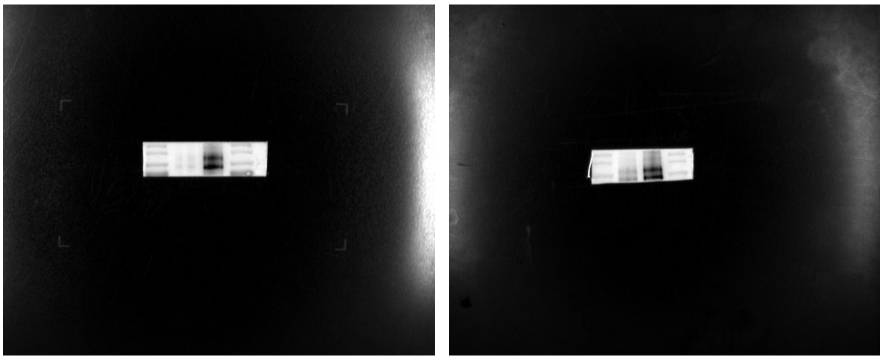

RANBP9 116kd

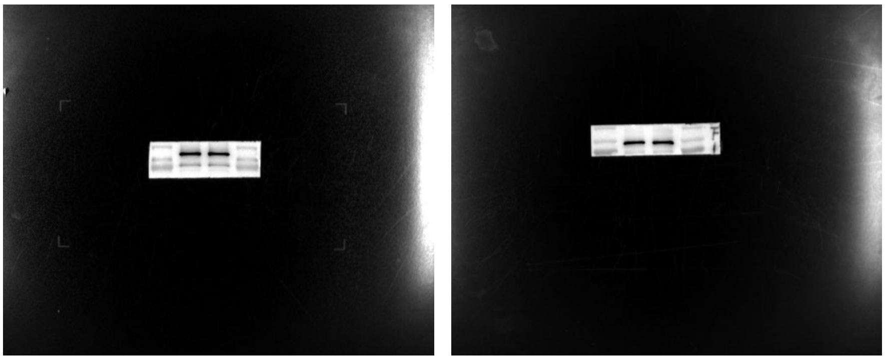

FAM111B 90kd

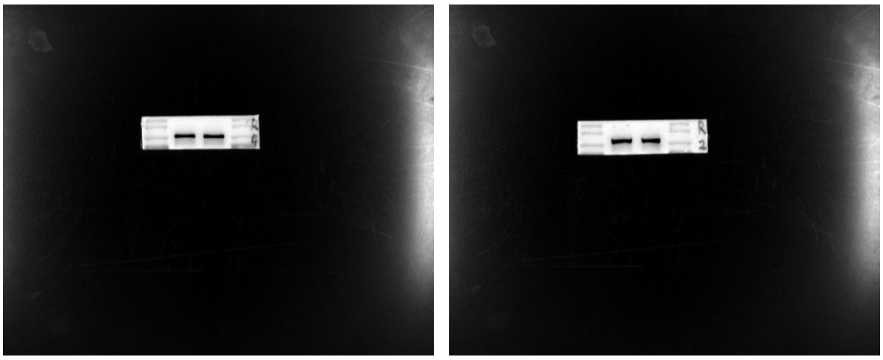

RANBP9 116kd

Fig. 5D

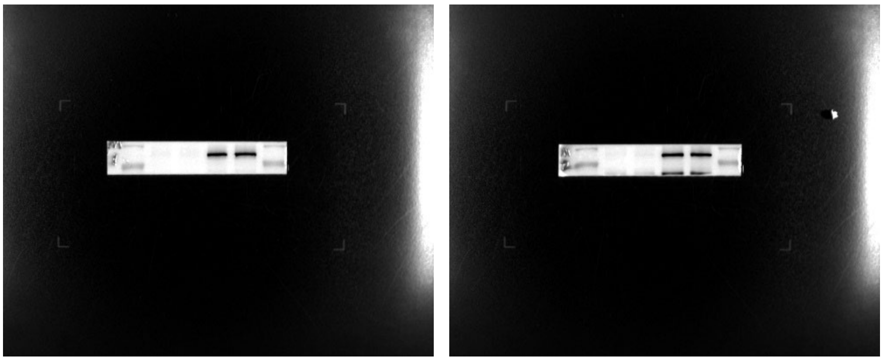

MFN2 80kd

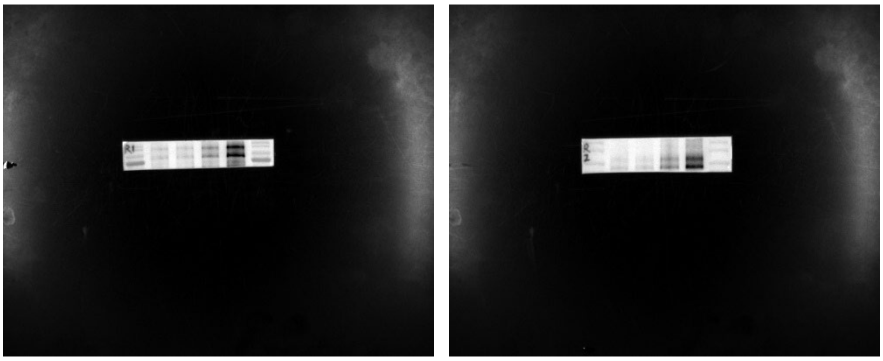

RANBP9 116kd

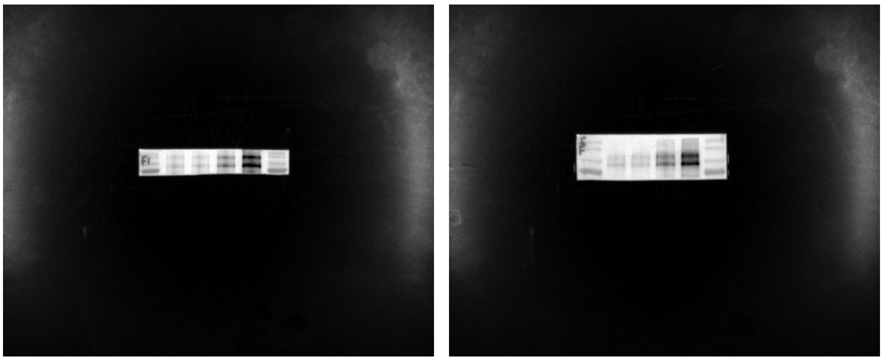

FAM111B 90kd

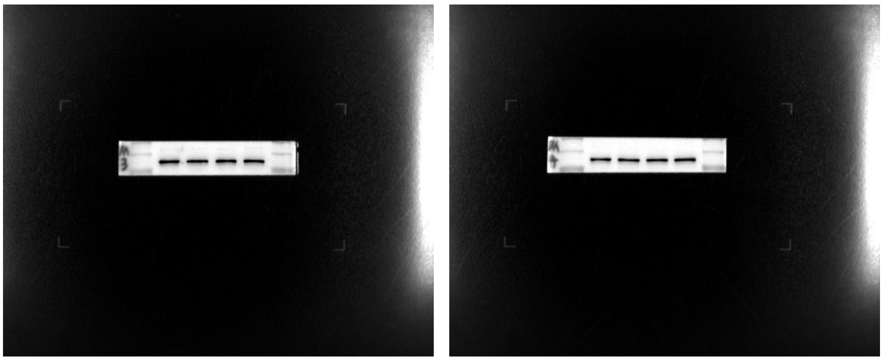

MFN2 80kd

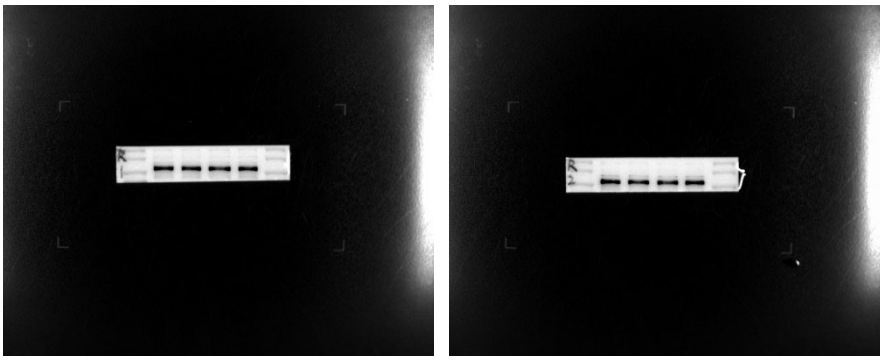

RANBP9 116kd

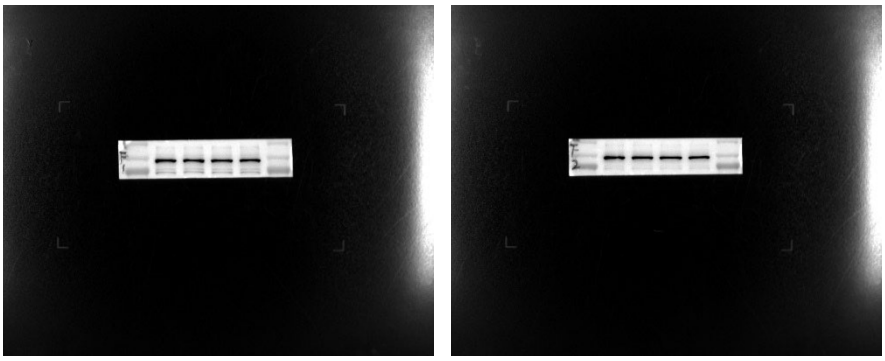

FAM111B 90kd

Fig. 5E

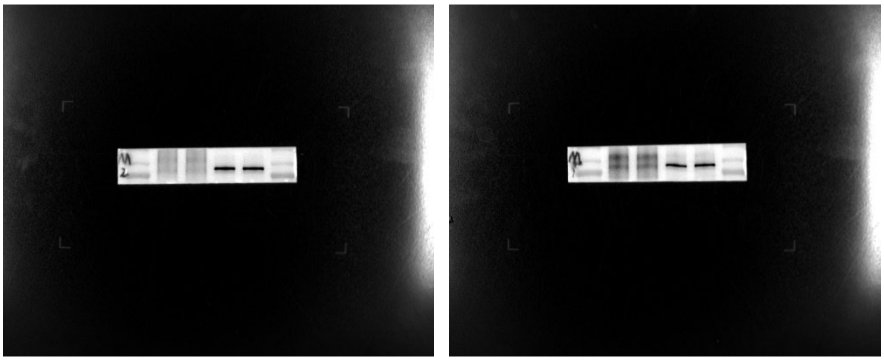

MFN2 80kd

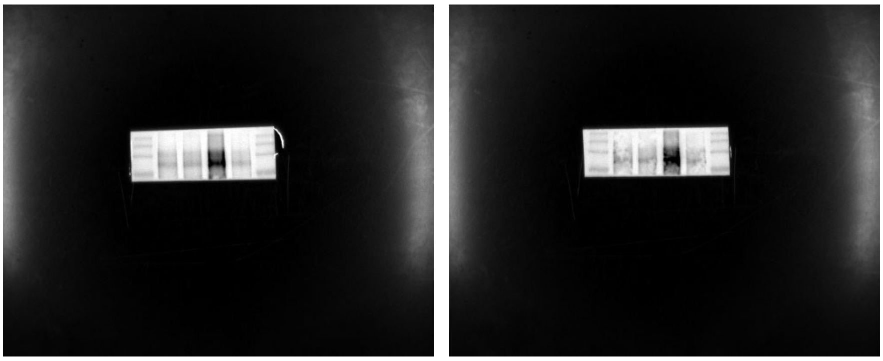

RANBP9 116kd

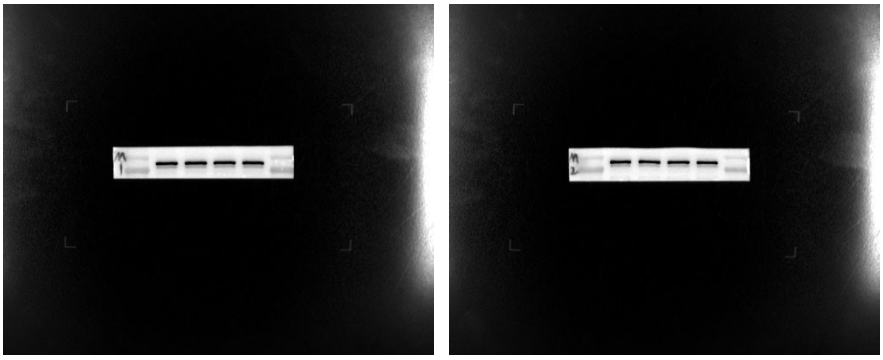

MFN2 80kd

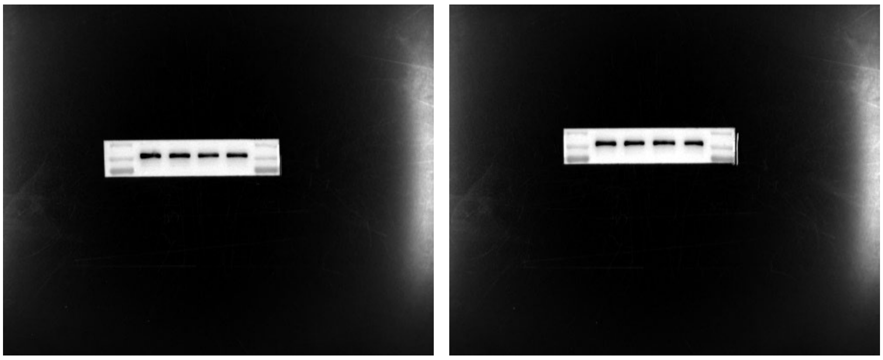

RANBP9 116kd

Fig. 5F

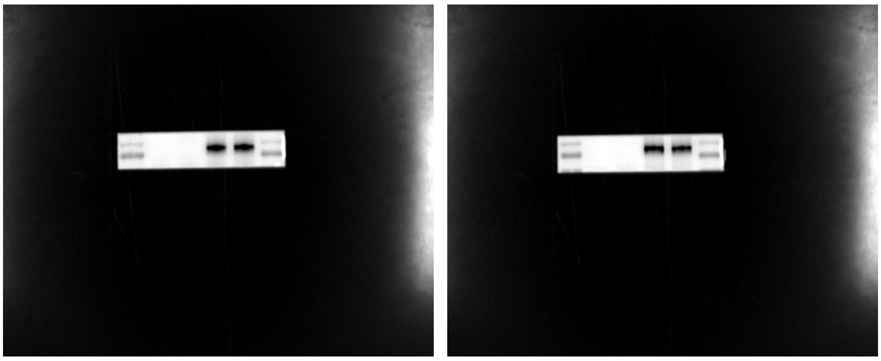

MFN2 80kd

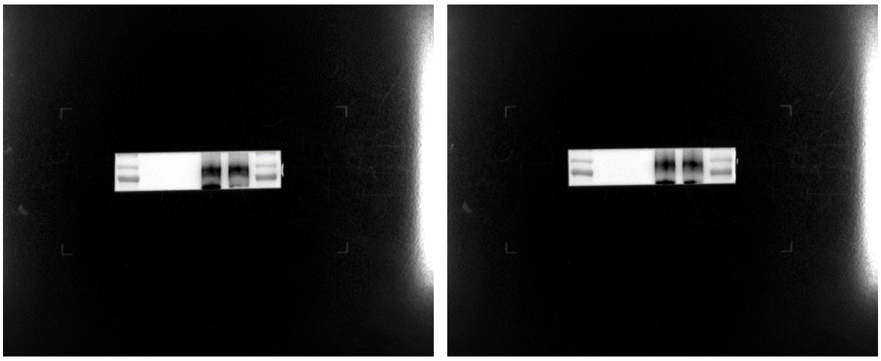

FAM111B 90kd

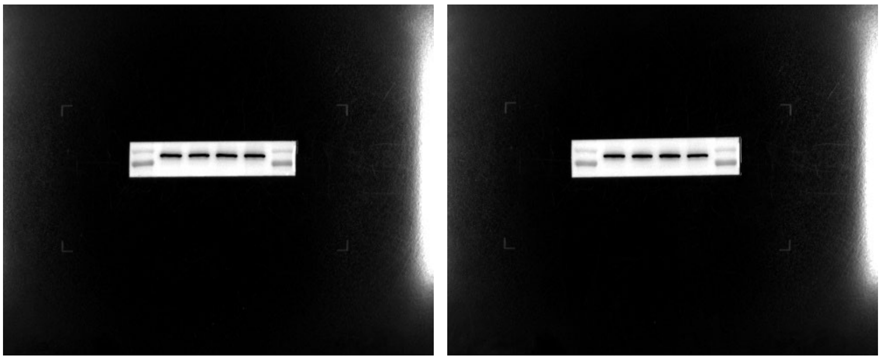

MFN2 80kd

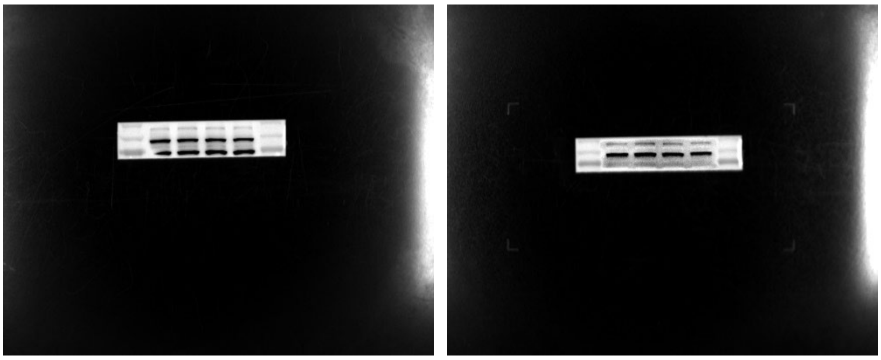

FAM111B 90kd

Fig. 5G

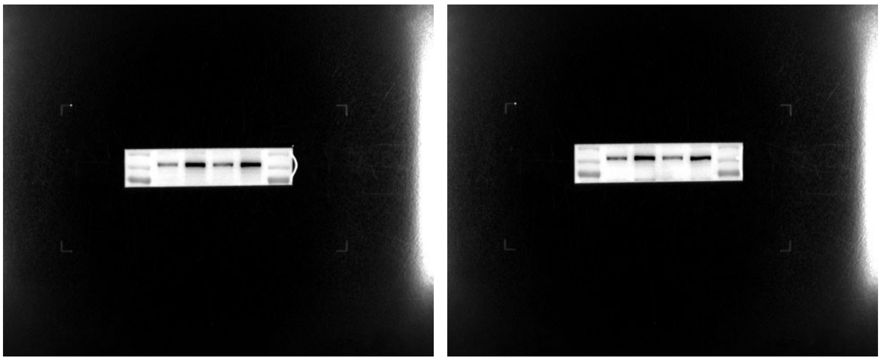

RANBP9 116kd

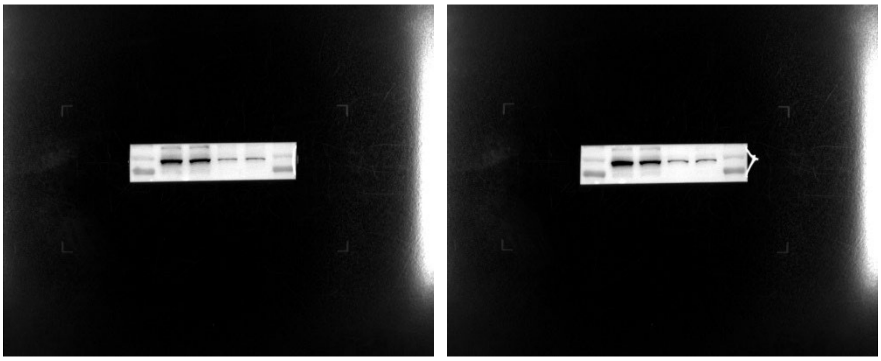

FAM111B 90kd

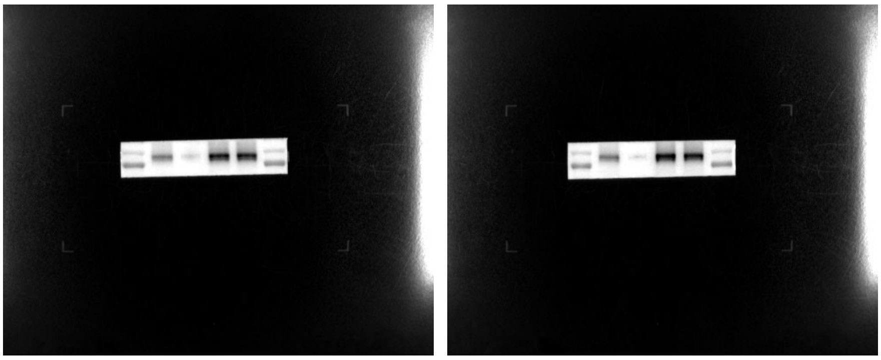

MFN2 80kd

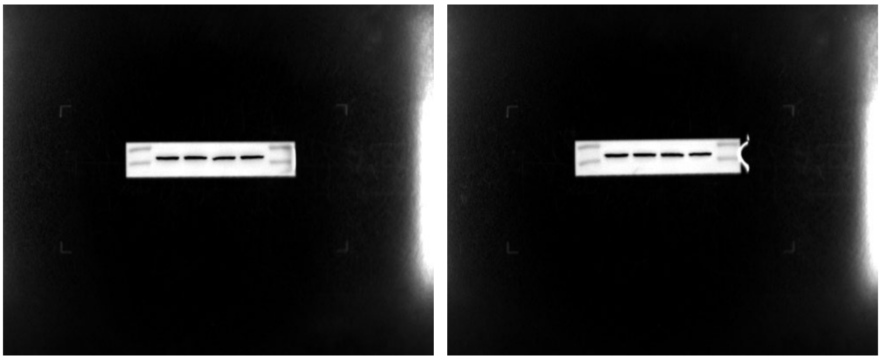

Actin 42kd

Fig. 5H

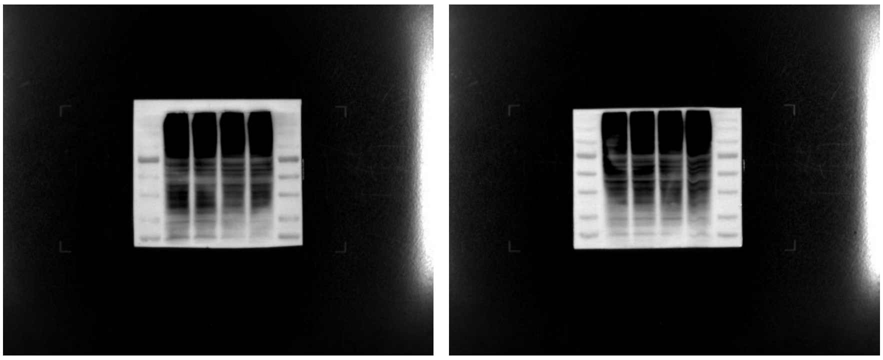

Input-Ub

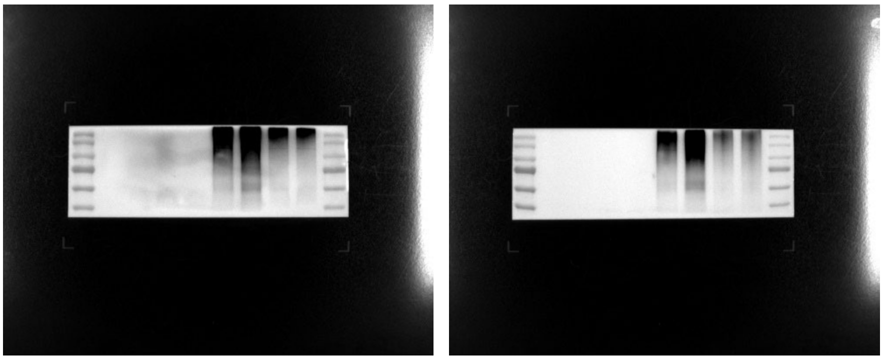

IP-Ub

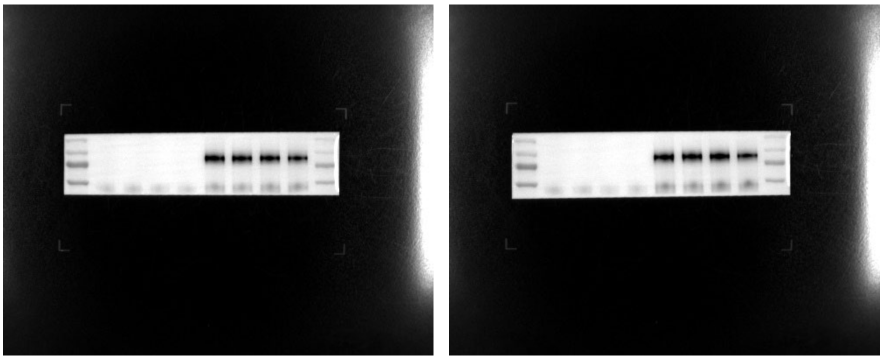

MFN2 80kd

Fig. S5A

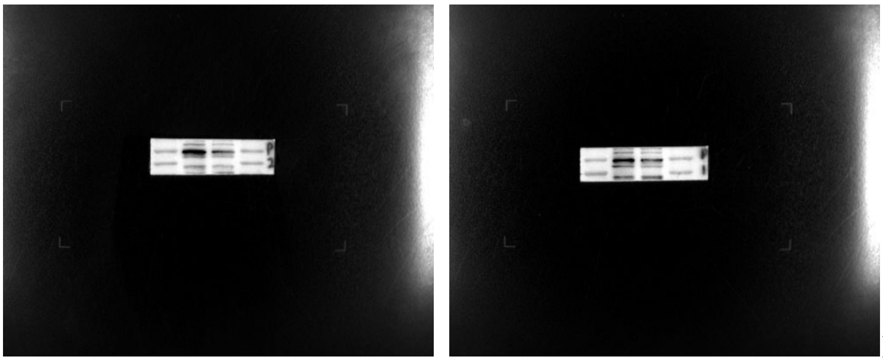

Parkin 52kd

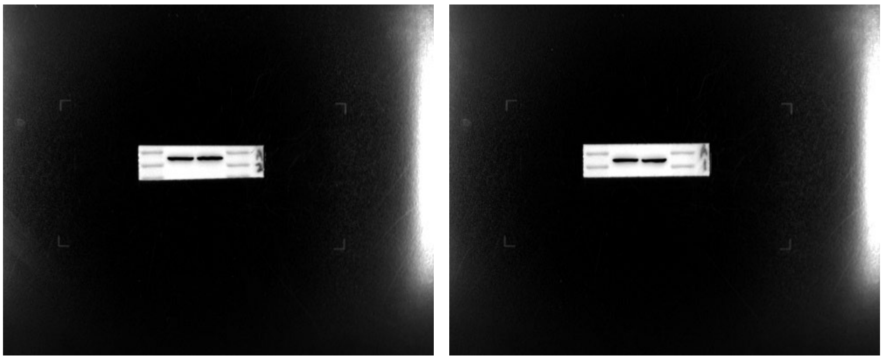

Actin 42kd

Fig. S5B, C

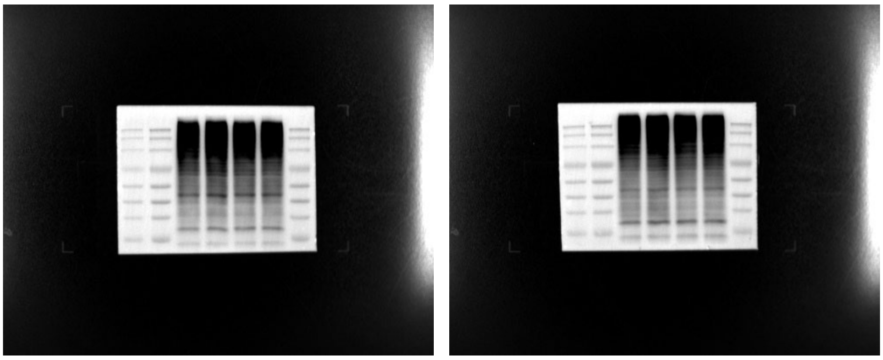

Input-Ub

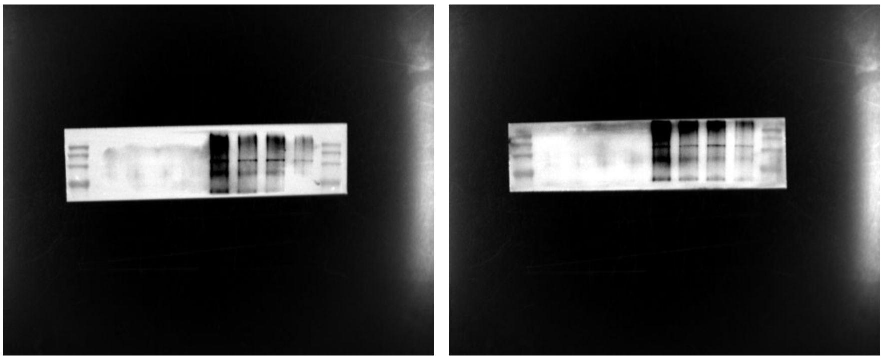

IP-Ub

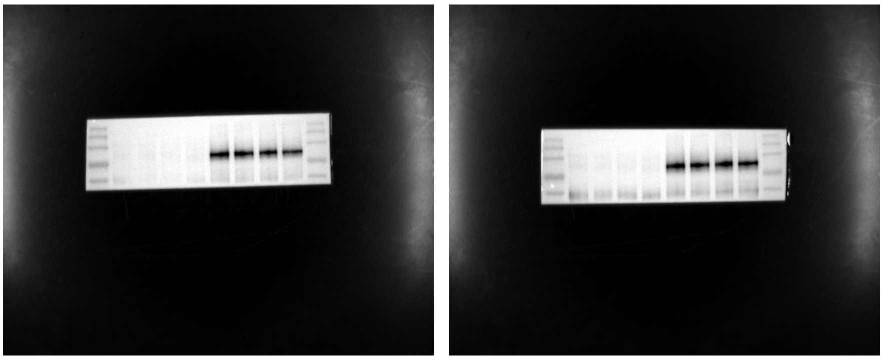

MFN2 80kd

Fig. S5D

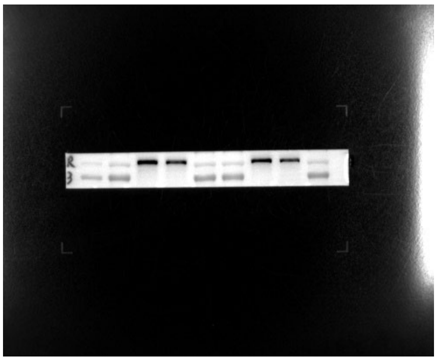

RANBP9 116kd

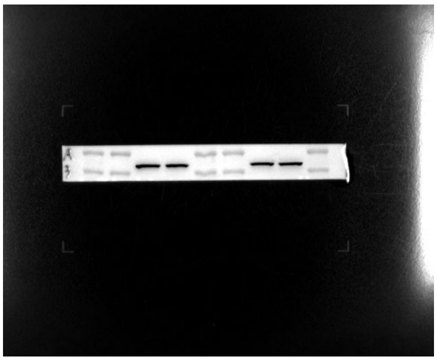

Actin 42kd

Fig. S5E

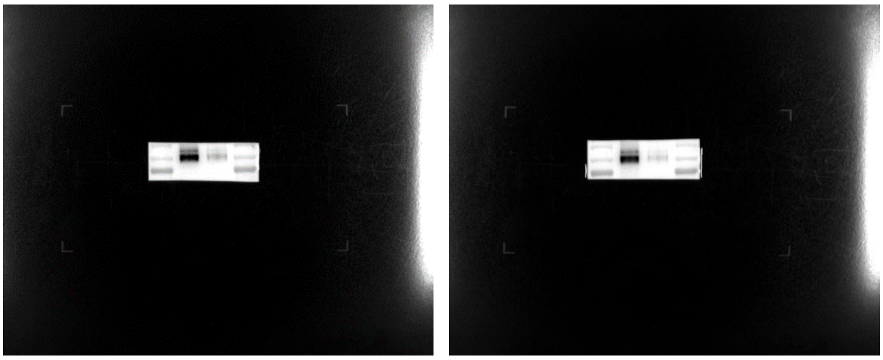

RANBP9 116kd

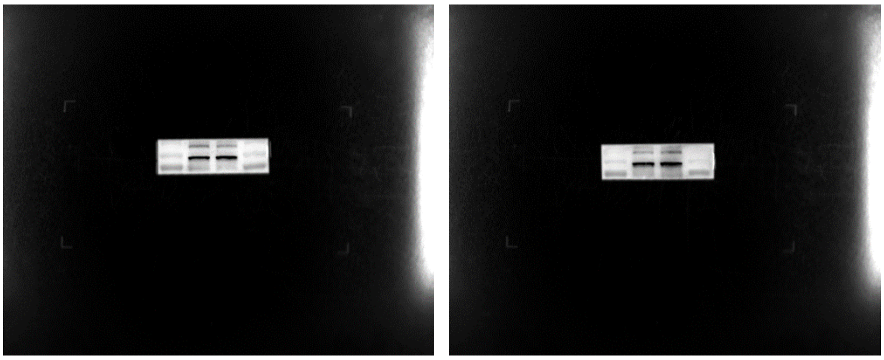

FAM111B 90kd

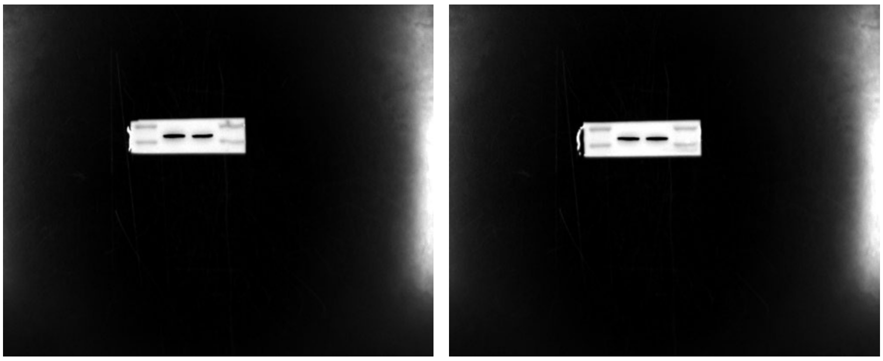

Actin 42kd

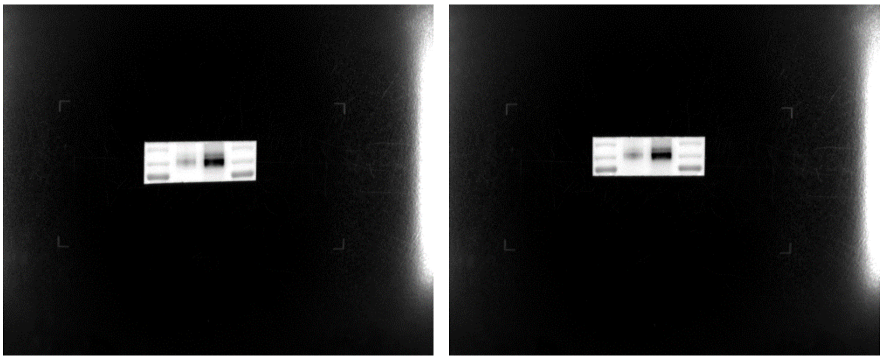

RANBP9 116kd

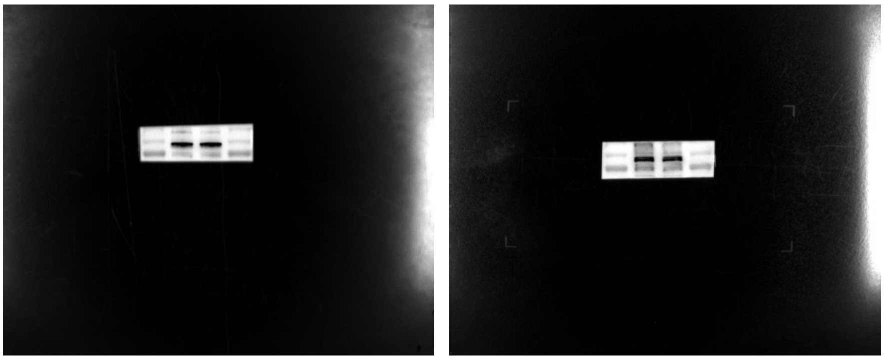

FAM111B 90kd

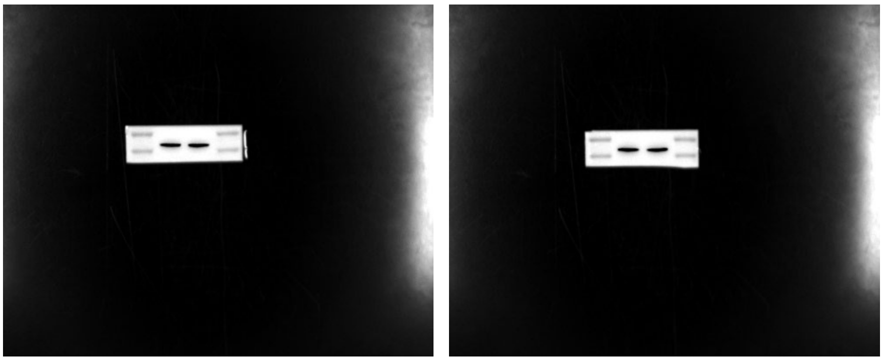

Actin 42kd

Fig. S5F

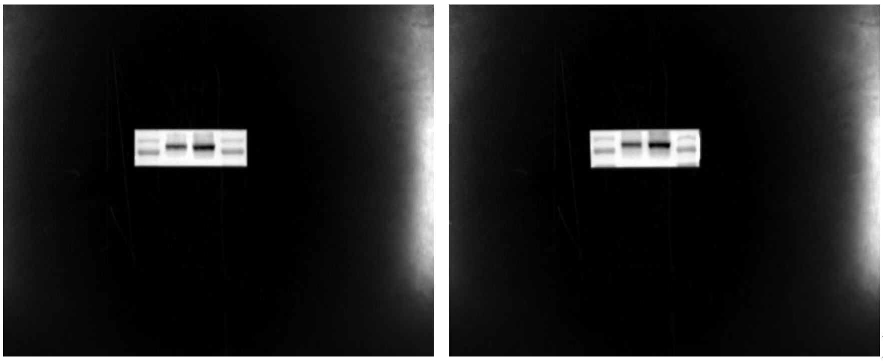

MFN2 80kd

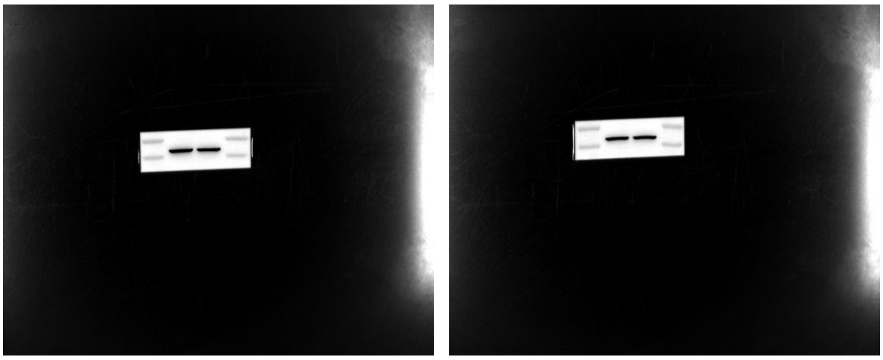

Actin 42kd

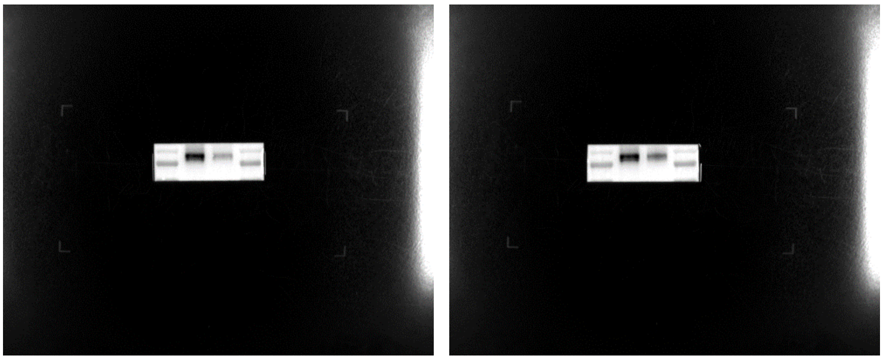

MFN2 80kd

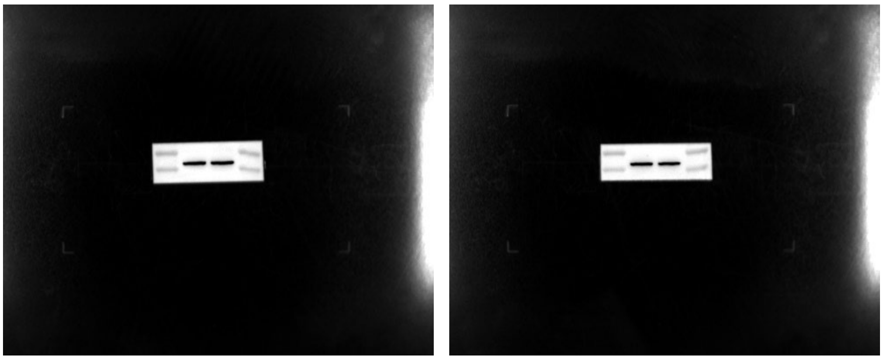

Actin 42kd

Fig. S5G

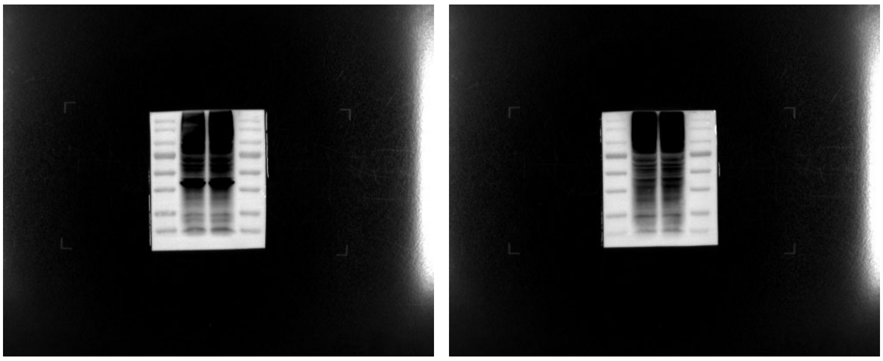

Input-Ub

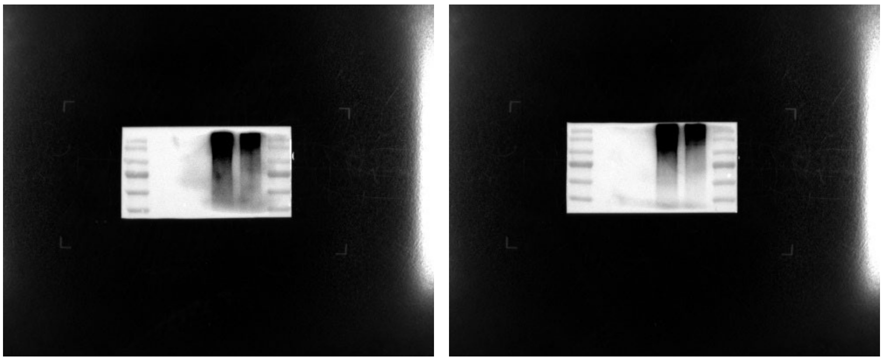

IP-Ub

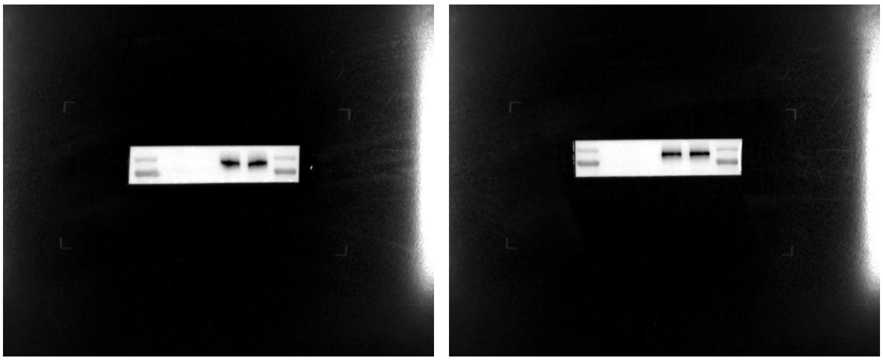

MFN2 80kd

Fig. S5H

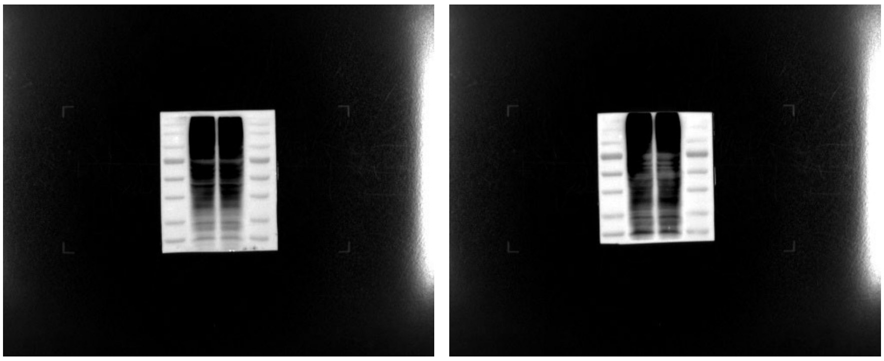

Input-Ub

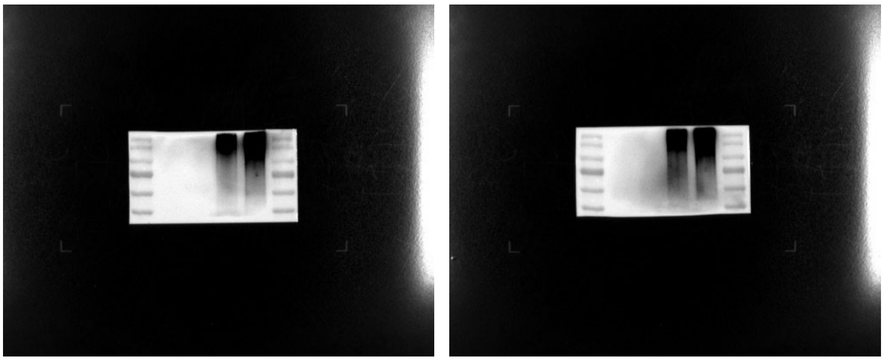

IP-Ub

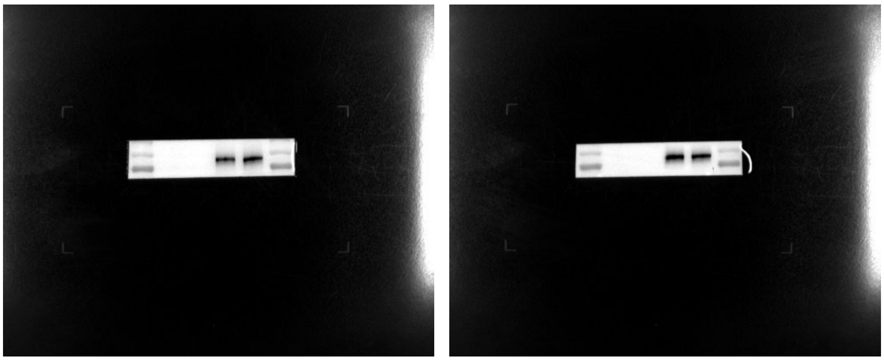

MFN2 80kd

Fig. S5I

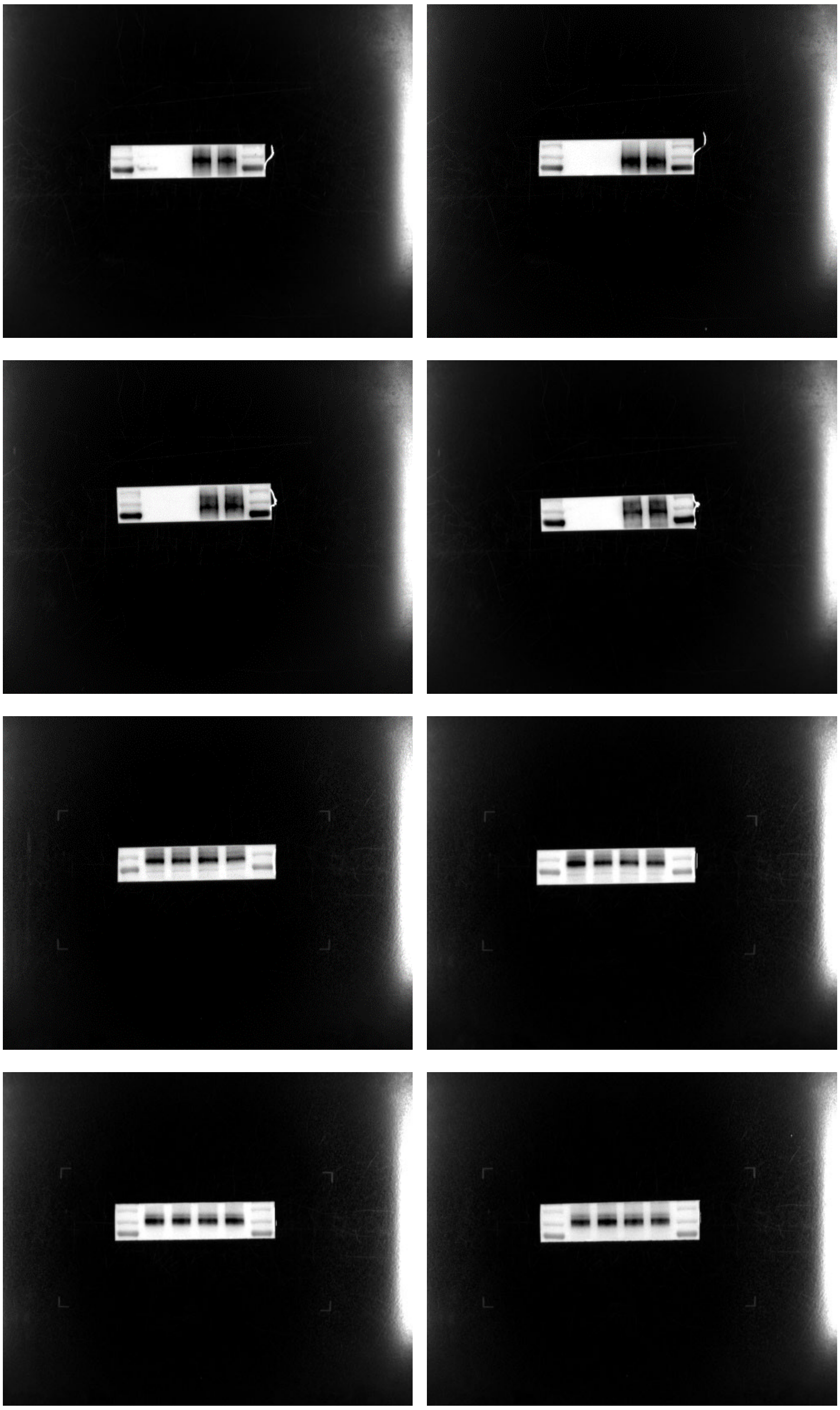

Fig. 6B

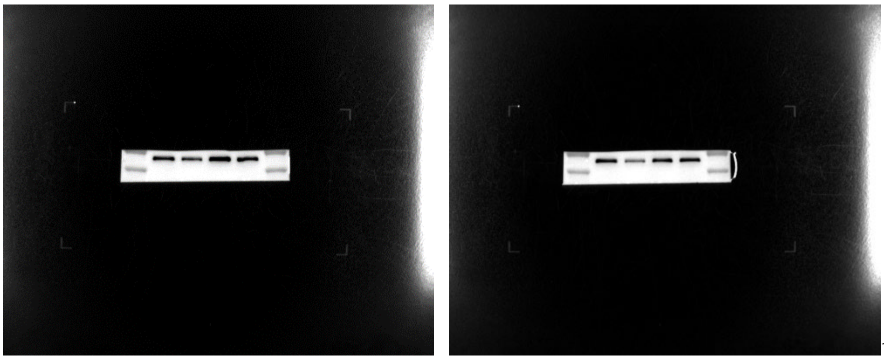

p62 62kd

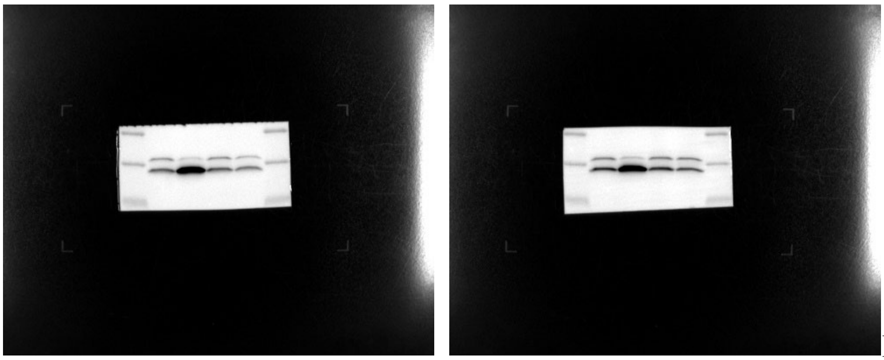

LC3 15kd

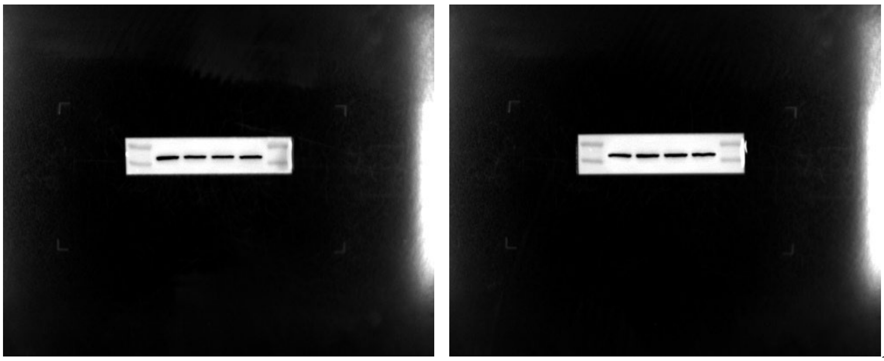

Actin 42kd

Fig. 6E

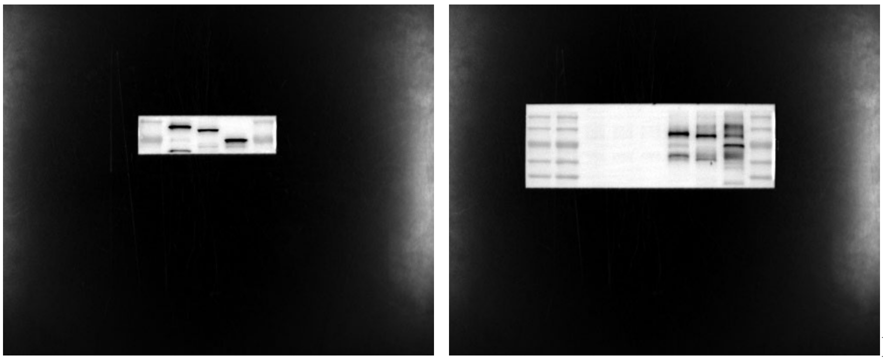

HA-FAM111B

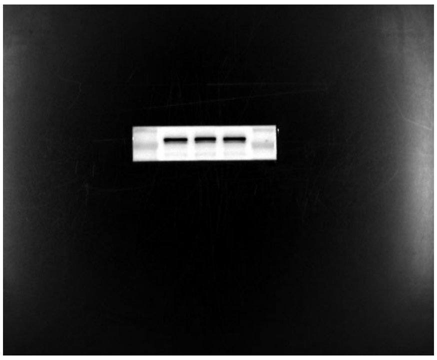
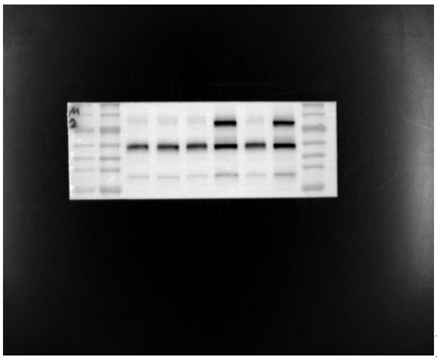

Myc-MFN2

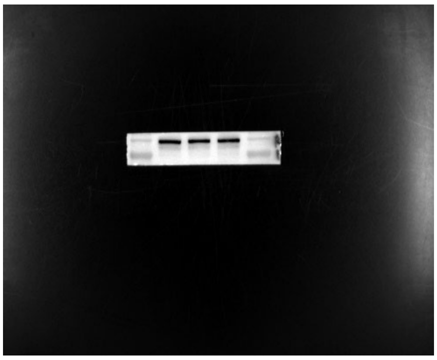
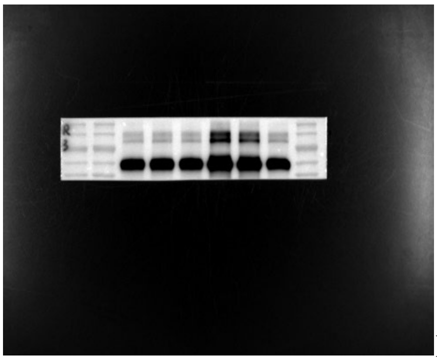

Flag-RANBP9

Fig. 6F

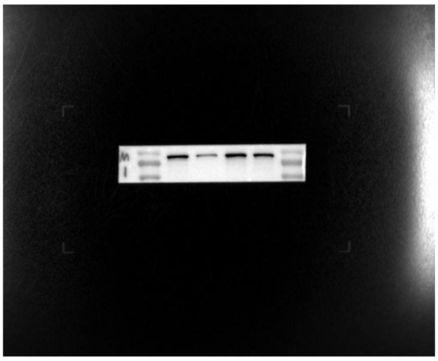
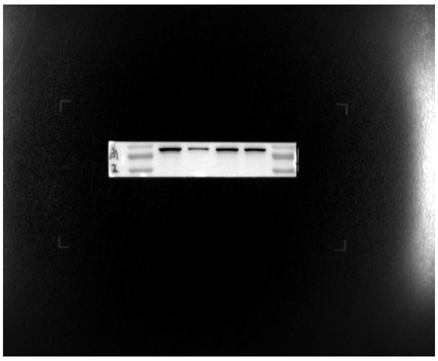

MFN2 80kd

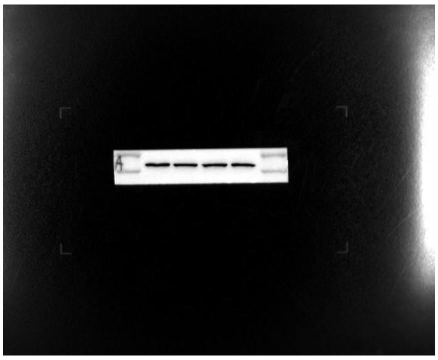
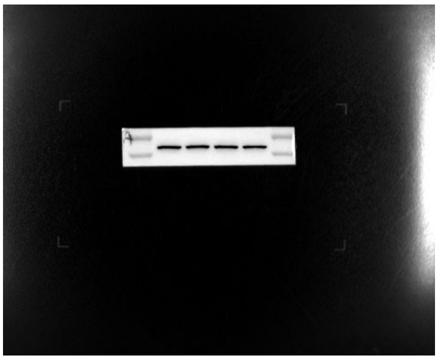

Actin 42kd

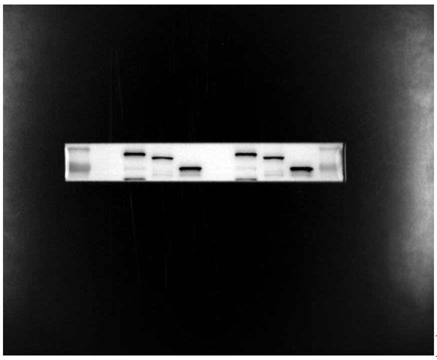

HA-FAM111B

Fig. 6G

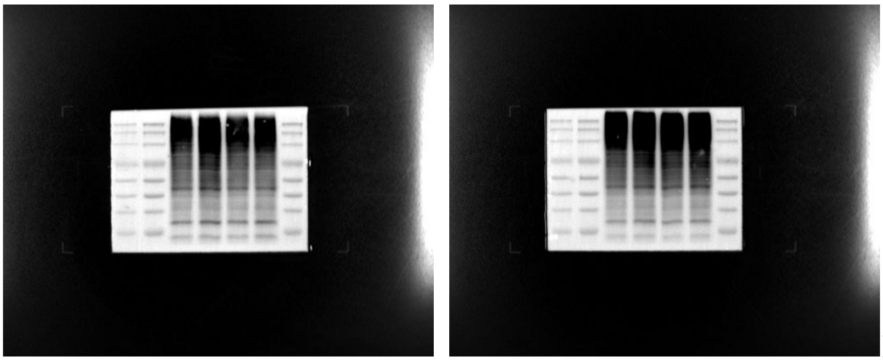

Input-Flag-Ub

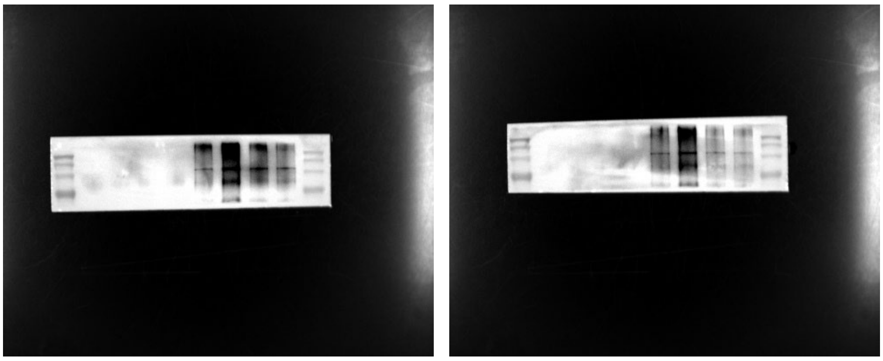

IP-Flag-Ub

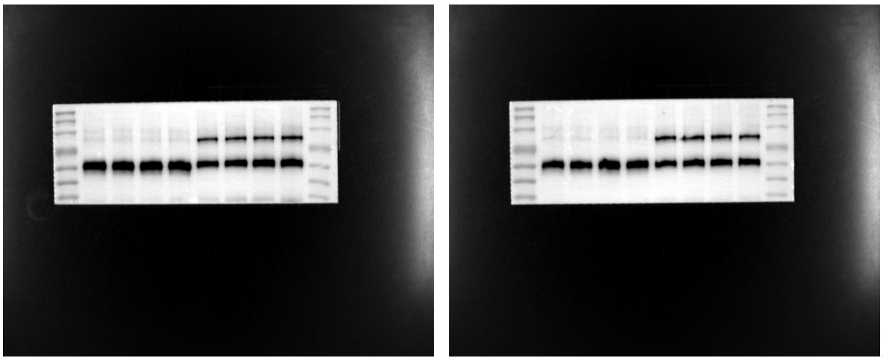

IP-MFN2 80kd

Fig. 7A

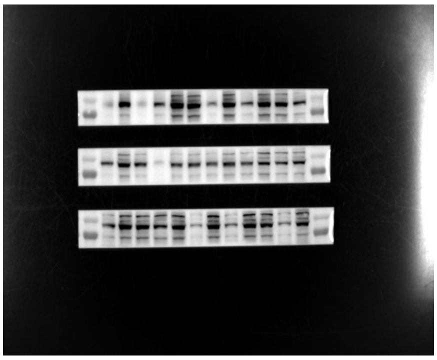

FAM111B 90kd

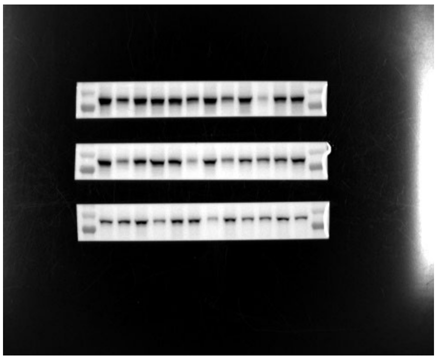

MFN2 80kd

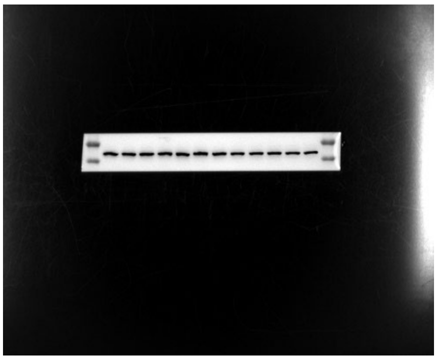

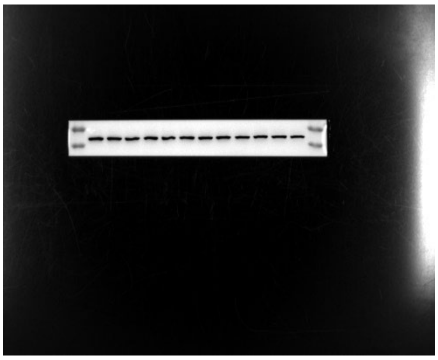

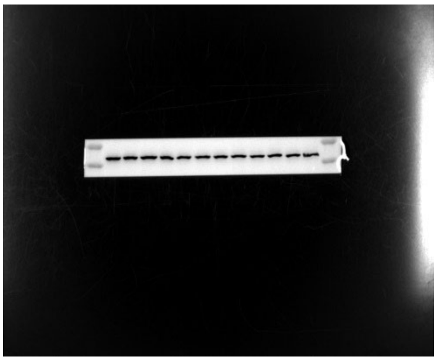

Actin 42kd

Fig. 8K

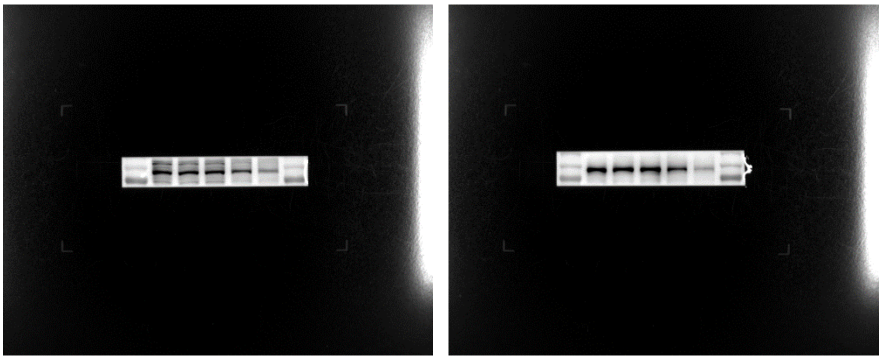

FAM111B 90kd

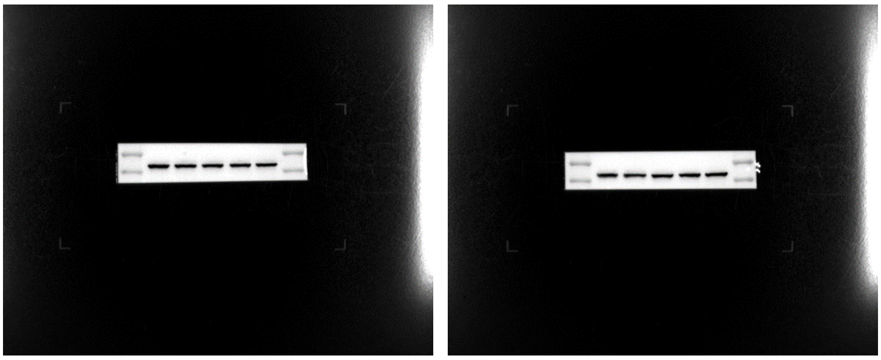

Actin 42kd
